# Hydrogenated borophene enabled synthesis of multielement intermetallic catalysts

**DOI:** 10.1038/s41467-023-43294-z

**Published:** 2023-11-16

**Authors:** Xiaoxiao Zeng, Yudan Jing, Saisai Gao, Wencong Zhang, Yang Zhang, Hanwen Liu, Chao Liang, Chenchen Ji, Yi Rao, Jianbo Wu, Bin Wang, Yonggang Yao, Shengchun Yang

**Affiliations:** 1 MOE Key Laboratory for Non-equilibrium Synthesis and Modulation of Condensed Matter, School of Physics, Xi’an Jiaotong University, Xi’an, 710049 PR China; 2 National Innovation Platform (Center) for Industry-Education Integration of Energy Storage Technology, Xi’an Jiaotong University, Xi’an, 710049 PR China; 3 Shaanxi Coal Chemical Industry Technology Research Institute Co., Ltd, Xi’an, 710100 PR China; 4 State Key Laboratory of Metal Matrix Composites, School of Materials Science and Engineering, Shanghai Jiao Tong University, Shanghai, 200240 PR China; 5 Hydrogen Science Research Center, Zhangjiang Institute for Advanced Study, Shanghai Jiao Tong University, Shanghai, 200240 PR China; 6 State Key Laboratory of Materials Processing and Die & Mould Technology, School of Materials Science and Engineering, Huazhong University of Science and Technology, Wuhan, 430074 PR China; 7 State Key Laboratory of Chemistry and Utilization of Carbon Based Energy Resources, School of Chemical Engineering and Technology, Xinjiang University, Urumqi, 830017 PR China

**Keywords:** Fuel cells, Fuel cells, Electrocatalysis

## Abstract

Supported metal catalysts often suffer from rapid degradation under harsh conditions due to material failure and weak metal-support interaction. Here we propose using reductive hydrogenated borophene to in-situ synthesize Pt/B/C catalysts with small sizes (~2.5 nm), high-density dispersion (up to 80 wt%_Pt_), and promising stability, originating from forming Pt-B bond which are theoretically ~5× stronger than Pt-C. Based on the Pt/B/C module, a series (~18 kinds) of carbon supported binary, ternary, quaternary, and quinary Pt intermetallic compound nanocatalysts with sub-4 nm size are synthesized. Thanks to the stable intermetallics and strong metal-support interaction, annealing at 1000 °C does not cause those nanoparticles sintering. They also show much improved activity and stability in electrocatalytic oxygen reduction reaction. Therefore, by introducing the boron chemistry, the hydrogenated borophene derived multielement catalysts enable the synergy of small size, high loading, stable anchoring, and flexible compositions, thus demonstrating high versatility toward efficient and durable catalysis.

## Abstract (web summary)

Supported metal catalysts suffer from rapid degradation under harsh conditions. Here, the authors report a hydrogenated borophene-triggered synthesis method to prepare carbon supported Pt and multielement Pt based intermetallic catalysts with enhanced catalytic activity and durability for oxygen reduction reaction.

## Introduction

The global consensus on achieving carbon neutrality has sparked growing interest in reducing fossil fuel usage and increasing the share of clean energy in our energy mix in the coming years^1–3^. In this context, well-supported metal nanoparticle catalysts have emerged as crucial components for catalytic energy conversion and storage technologies, including fuel cells, water splitting, and steam reforming reactions^4,5^. Among them, electrocatalysts based on platinum-group metals (PGM) have demonstrated significant potential for enhancing the efficiency of these reactions by overcoming their sluggish kinetics. Substantial progress has been made in improving their catalytic activity^6,7^. However, besides the considerable cost associated with PGM catalysts, their stability is often compromised during prolonged operation in harsh environments characterized by high temperatures, redox atmospheres, and strongly acidic or alkaline chemical solutions. These challenges primarily stem from two underlying factors: material failure, such as metal dissolution, and weak interaction between the metal catalyst and its supporting material, leading to catalyst sintering. Consequently, both the active site density and intrinsic activity are significantly reduced, resulting in a pronounced degradation in performance.

One viable strategy for enhancing the stability of PGM catalysts is the formation of stable intermetallic nanoparticles. The strong enthalpic interaction between PGM and non-noble elements gives rise to stronger bonding, surpassing that of Pt-Pt interactions, such as Pt-Co, resulting in distinct long-range atomic ordering with well-defined stoichiometry. This characteristic demonstrates exceptional stability during complex reactions^8^. Nevertheless, high-temperature annealing is often required to induce long-range ordering and then form intermetallic particles, which is always accompanied by strong atom migration and therefore inevitably causes particle sintering^9,10^.

Another significant approach involves capitalizing on the strong metal-support interaction (SMSI) to effectively anchor PGM nanoparticles onto the support. This anchoring mechanism facilitates the dispersion and stabilization of nano catalysts in challenging environments by impeding their migration and growth over extended operation periods^11,12^. Such SMSI effect was generally realized by bonding coordinatively unsaturated metal atoms with different supports to form coupling pairs, including M-O, M-S, M-P, M-N, and M-F (M = metals)^13,14^. Alternatively, defects on the substrate surface, such as oxygen and metal atom vacancies, can also be utilized to capture and stabilize the PGM nanoparticles^15^. The existence of the SMSI effect can effectively suppress nanoparticle agglomeration even at temperatures up to 1000 °C^16^, facilitating the preparation of Pt intermetallic nanoparticles with high dispersion and small sizes. However, these strategies commonly face complexity in the preparation process and high selectivity towards carriers, leading to increased manufacturing difficulty and application cost of the catalyst^17^. Therefore, developing an easy-to-implement SMSI strategy for the facile preparation of Pt-based intermetallic catalysts with high versatility and robustness remains a significant challenge.

Recently, the free-standing 2-dimensional (2D) hydrogen boride nanosheet, known as hydrogenated borophene or hydrogen borophene (HB), has garnered increasing recognition in the fields of chemistry, material science, nanotechnology, and condensed matter physics. This is attributed to its remarkable properties, including a large surface area and excellent mechanical and electrical characteristics^18^. It is worth noting that the active hydrogen present in HB exhibits a notable reducibility, making it a valuable reducing agent for the synthesis of catalysts and related materials^19–22^.

In this study, we present a hydrogenated borophene (HB)-triggered synthesis method for multielemental Pt intermetallic compounds (IMCs). This innovative approach combines the reducibility of HB with the in-situ formed boron (B) sheets, leading to a strong metal-support interaction (SMSI) effect, which results the catalysts possessing several highly desirable characteristics, including much small size, high-loading capacity, and exceptional stability. Experimental analysis and theoretical calculations provide insights into the crucial role played by the stable Pt-B bond within the Pt/B/C system. It enables high-density dispersion of Pt nanoparticles and prevents sintering, even under high-temperature annealing conditions. In addition, the high specific surface area of 2D structured HB and the subsequent B sheet makes it easy to adsorb on the surfaces of various supports, such as nanocarbon (e.g., carbon black, carbon nanotubes, graphene) and metal oxides (e.g., Al_2_O_3_, TiO_2_, CeO_2_), which promotes the growth and stabilization of Pt nanoparticle on such supports, thus greatly expands the versatility of this strategy. As a result of the Pt-B interaction induced SMSI effect, a series of binary, ternary, quaternary, and quinary Pt-based multielement intermetallic nanoparticle catalysts with a sub-4 nm size and uniform distribution are synthesized even at the annealing temperature up to 1000 °C. Notably, these Pt IMCs catalysts exhibit much enhanced catalytic activity and durability for oxygen reduction reaction (ORR) compared to commercial catalysts. These improvements can be attributed to the comprehensive advantages offered by the hydrogenated borophene-triggered multielement catalysts, which include their much small size, high stability, and composition versatility.

## Result

### The versatility of the HB-induced in situ reduction method

**Fig. 1:**
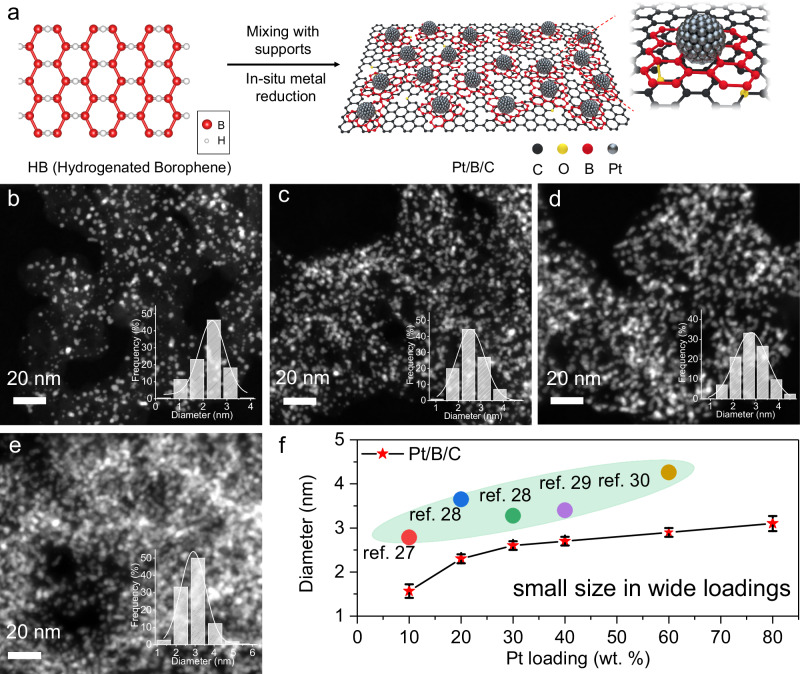
Synthesis of Pt/B/C with different loading. **a** Schematic illustration of the process of borophene-stabilized metal catalysts. **b**–**e** HAADF-STEM images of Pt/B/C with Pt loading amounts of 20, 40, 60 and 80 wt%, respectively. The inserted histograms are the statistics of particle size distribution of corresponding loadings. **f** Size distribution of Pt with increasing loading amount for the Pt/B on KB as well as those from the previous literature shown in shaded area. The error bars in **f** show the standard deviation in at least 200 particle size measurements in different regions for each datum.

HB was produced by exfoliation and ion exchange between protons and magnesium cations in magnesium diboride (MgB_2_) at room temperature, as reported by Nishino et al. and our previous work^21,23^. The structure of HB is hypothesized to consist of *sp*^2^-bonded boron planes, forming a hexagonal boron network that is bridged by hydrogen atoms without long-range order (Fig. 1a and Supplementary Fig. 1)^21,23–25^. The hydrogen in HB exhibited a notable reducibility, as confirmed through a series of control experiments (see Supplementary Fig. 2). This property enables the in-situ reduction of PGM ions to the form the small metal nanoparticles.

In the experiment, we conducted a two-step synthesis procedure to fabricate the catalyst, as illustrated in Fig. 1a. Firstly, the as-prepared HB was mixed with activated carbon to form HB/C support. Subsequently, a solution containing H_2_PtCl_6_ was introduced, where the Pt^4+^ ions was then reduced by the bridging hydrogen atoms in HB, along with the split of hexagonal boron network, due to the breakup of the terminal B-H bond (Supplementary Fig. 3). The following growth of Pt nanoparticles was on the basis of the in-situ formed Pt clusters that has been already anchored on the surface of C through B layer. Generally, increasing the loading amount of Pt would decrease the inter-particle distance, thereby promoting severe sintering through particle coalescence and Ostwald ripening^26^. However, in our case, even with an increased loading percentage of metallic Pt on carbon (specifically Ketjen black, ECP-600JD, KB) from 10% to as high as 80%, the Pt nanoparticles still maintained a uniform and small size (Fig. 1b–f and Supplementary Fig. 4). Compared with other reports and commercial Pt/C catalysts^27–30^, the Pt/B/C catalysts exhibit an exceptionally small size and a uniform, high-density distribution without noticeable aggregation with the same loading amount, which indicates the presence of a robust interaction between the metal and the in-situ formed boron fragments on carbon surface, effectively inhibiting the undesired sintering process between nanoparticles. Our additional experiments have revealed that, in contrast to the encapsulation mechanism often reported in the literature^31^, this stabilization effect is achieved by employing B fragments as a stabilizing medium between activated carbon and Pt nanoparticles (See Supplementary Fig. 5).

Moreover, our synthesis strategy exhibits high versatility by allowing the facile loading of metal nanoparticles onto various supports, such as carbon nanotubes, graphene, Al_2_O_3_, TiO_2_ and CeO_2_ et.al. (See Supplementary Fig. 6). This highlights the broad applicability of our approach for synthesizing various supported noble metal catalysts. Notably, metallic oxides, especially Al_2_O_3_, are widely used in high-temperature catalysts, yet often have a weak metal-support interaction and therefore low loading (usually <20 wt%_noble metal_) to prevent particle aggregation^32^. But in our case, high loading amounts of up to 30 wt% of Pt on commercial Al_2_O_3_ support are realized with an average particle size of less than 5 nm, which is otherwise impossible.

### The anti-sintering property of Pt/B/C

High-temperature heating would accelerate the coalescence and Ostwald ripening of metallic nanoparticles on the support surface, thus it is used to verify the stability and existence of strong interaction between metal and supports. To further explore the anti-sintering of Pt/B/C, in-situ transmission electron microscopy (TEM) was used to record the evolution of Pt/B/C with high Pt loading amount (60 wt%) annealing at a wide temperature range. As shown in Supplementary Fig. 7, the TEM images of Pt/B/C acquired from 25 °C to 1000 °C and corresponding size analysis show the well-maintained dispersion and limited growth of nanoparticles. Especially, the average size of nanoparticles remains consistently below 6 nm, even with a temperature elevation to 1000 °C. Moreover, there is no significant coalescence or Ostwald ripening of Pt nanoparticles observed in Pt/B/C nanocatalysts during the isothermal process at 600 °C, as demonstrated by the TEM images presented in Supplementary Fig. 8. Notably, the distance between two nanoparticles marked within red dotted circles exhibits negligible change, indicating the superior anti-sintering property of the Pt/B/C sample. Additionally, two distinctive single nanoparticles, highlighted by blue and green circles respectively, maintain their size and morphology without any noticeable changes, thereby demonstrating remarkable stability. In comparison, the commercial JM 60 wt%_Pt_ Pt/C exhibit severe sintering during the annealing process, resulting in a rapid increase in the average size to approximately 8 nm (Supplementary Figs. 9 and 10). To avoid the influence of transmission electron beam on the morphology of the catalyst during the in-situ observation and evaluate the stability of Pt/B/C under reducing conditions, the catalyst was subjected to annealing at different temperatures for 2 h in a tube furnace under a 5% H_2_/Ar atmosphere. The resulting morphology was characterized by TEM, and the corresponding images are presented in Supplementary Fig. 11. Similarly, the morphology and size of Pt nanoparticles in Pt/B/C catalyst only show a slight Ostwald ripening even the temperature up to 1000 °C, while the commercial JM Pt/C catalyst demonstrates noticeable coalescence starting at 400 °C (Supplementary Figs. 12 and 13).

### DFT calculation of Pt/B/C

The stability of Pt nanoparticles on a carbon support using the boron sheet in Pt/B/C against temperature can also be elucidated through DFT calculations^21^. To investigate the potential size dependence, models of Pt_*n* (*n*=10, 13, 15)_ /borophene and Pt_*n* (*n*=10, 13, 15)_ /graphene were constructed to approximate the Pt nanoparticle adsorption on the boron layer in Pt/B/C and on graphite carbon in Pt/C, respectively (Supplementary Fig. 14). As shown in Supplementary Fig. 15, although the Gibbs adsorption free energy (ΔG) fluctuates slightly with the number of Pt atoms, the absolute ΔG value of Pt_*n*(*n*=10,13,15)_ /borophene (Pt-B interaction) shows, in general, five times more than that of the Pt_*n*(*n*=10,13,15)_ /graphene model (Pt-C interaction). This strong interaction was attributed to the natural electron-deficient properties of the B which leads to the electrons transferring between B and metal^33^. Additionally, the anti-sintering ability of Pt/B was also demonstrated by simulating the Ostwald ripening processes which involves the migration of individual atom driven by surface energy of nanoparticles. We calculated the escape energy of the atom in Pt_13_ which is furthest from the substrate (graphene or borophene). Notably, the escape energy of individual atom from Pt_13_ cluster on borophene (4.68 eV) was obviously larger than that on graphene (3.16 eV) (Supplementary Fig. 16). These calculation results confirmed that the strong interaction between metal and B sheet is the key reason for improving the thermal stability of Pt nanoparticles^34^. Furthermore, the bonding energy between graphene and borophene layers at the equilibrium position was reported higher than that between graphene layer (−0.035 eV/atom vs. −0.02 eV/atom), indicating a stronger adsorption of carbon toward borophene fragments, which is also conductive to the stabilization of Pt nanoaprticles on support^35^.

### Experimental analysis of Pt/B/C

**Fig. 2:**
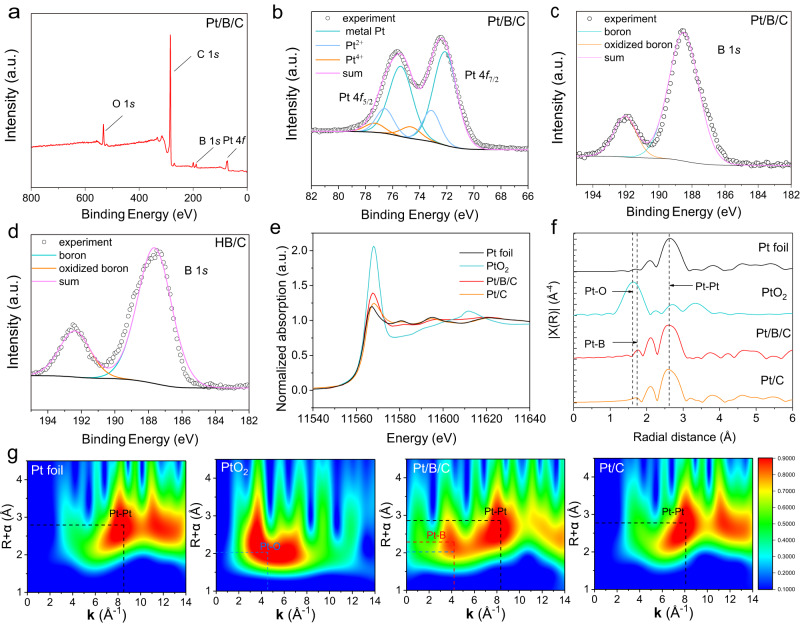
Interaction of Pt-B analyzed in the experiment. **a** Survey spetra XPS of Pt/B/C. **b** Pt 4*f* XPS of Pt/B/C. **c** B 1*s* XPS of Pt/B/C. **d** B 1*s* XPS of HB/C. **e** Pt L_3_-edge XANES spectra of Pt/B/C, along with PtO_2_, Pt foil and commercial Pt/C. **f** Pt L_3_-edge EXAFS spectra of Pt/B/C, with PtO_2_, Pt foil and commercial Pt/C. **g** WT-EXAFS of the Pt L_3_-edge signal of the Pt foil, PtO_2_, Pt/B/C and commercial Pt/C. Normalized WT intensity was described by colorbar.

X-ray photoelectron spectroscopy (XPS) was used to analyze the interaction between Pt and B. Fig. 2a presents the XPS survey spectra of the Pt/B/C sample, where distinct Pt 4*f* and B 1*s* signals can be resolved from the spectrum. The high-resolution XPS of Pt 4*f* show that the binding energy (BE) of Pt 4*f* shifts to higher value relative to the reference metal Pt^36^, demonstrating the potential electron transfer from Pt to B. The B 1*s* obtaining from the sample of Pt/B/C and spectra of HB/C are shown in Fig. 2c, d and Supplementary Fig. 17, respectively. The binding energy higher than 190 eV is attributed to B-O bonds, and the bonds present between 187.5 eV and 189 eV are attributed to B-B bonds^19,23,37^. Compared to the B1*s* peak position in HB/C, the B 1*s* peak in Pt/B/C samples exhibits a positive shift of approximately 1.1 eV. This trend is consistent with the results reported by Kawamura et al.^19^, which demonstrated a similar upward shift in the B 1*s* peak position after UV-irradiation-induced H_2_ release from HB. It suggests that the consumption of active H in HB by Pt^4+^ also leads to a decrease in electron density around the B atom, causing the observed positive peak shift.

To further validate the Pt-B interaction in Pt/B/C, we investigated the electronic structure and local coordination environment of Pt atoms through Pt L_3_-edge X-ray absorption near-edge structure (XANES) and Fourier-transform extended X-ray absorption fine structure (EXAFS) on Pt/B/C and compared it with reference materials (PtO_2_ powder, JM Pt/C and Pt foil). Fig. 2e shows the normalized spectra of Pt L_3_-edge XANES of different samples. The white-line intensities in the spectra, as a function of the unoccupied Pt 5d states, reflect the oxidation states of the Pt species in the samples^13,38^. It can be seen that the white-line intensity of Pt/B/C is slightly higher than Pt foil which suggests a decrease in electron density of Pt, implying an electron transfer from Pt to B.

Fourier transform of EXAFS data in *R* space is shown in Fig. 2f. Compared to Pt-O bond (located at 1.63 Å) in PtO_2_, the apparent peak located at 1.78 Å can be assigned to Pt-B scattering. Subsequent fitting results further confirmed the existence of Pt-B bond in Pt/B/C with a bond length of 2.22 Å (Supplementary Fig. 18 and Table 2), consistent with the computation model after relaxation in DFT calculation (Supplementary Fig. 14). Furthermore, the corresponding wavelet transformed (WT)-EXAFS analysis was performed, which is a precise approach to distinguish the backscattering atoms even though they overlap substantially in R space. As shown in Fig. 2g, the Pt/B/C sample exhibits two maximums on y axis, one is located at *ca*. 2.8 Å which is ascribed to the Pt-Pt coordination, consistent with Pt foil reference, and the other (R*+α*: *ca*. 2.2 Å) which is different from Pt-O (R*+α*: *ca*. 2 Å) in PtO_2_ reference can be assigned to the Pt-B bond. The combination of these analyses conclusively demonstrates that the Pt is loaded on borophene, enhancing the adhesive energy between nanoparticles and the B/C support while suppressing aggregation and sintering of Pt nanoparticles at elevated temperatures.

### Synthesis of Pt-based IMCs

**Fig. 3:**
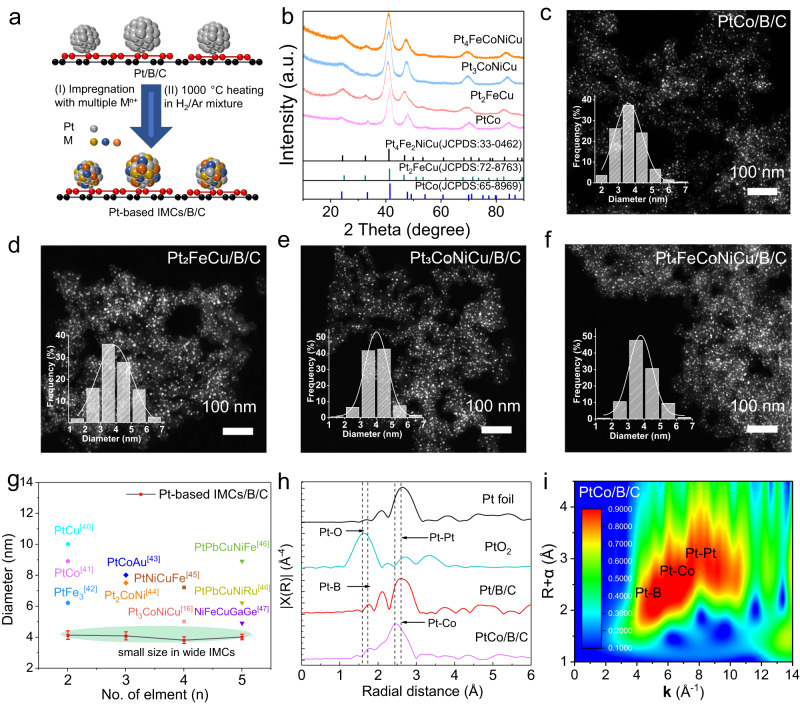
Synthesis of small Pt-based IMCs based on Pt/B/C. **a** Schematic illustration of the process of Pt-based multielement intermetallic catalysts. **b** Powder XRD patterns of Pt-based intermetallic alloy. **c**–**f** HAADF-STEM images of intermetallic alloy PtCo/B/C, Pt_2_FeCu/B/C, Pt_3_CoNiCu/B/C, Pt_4_FeCoNiCu/B/C. The inserted histograms are the statistics of particle size distribution of corresponding IMCs. **g** Comparison of intermetallic alloy size between our work with the state-of-art in the literature. The shaded areas represent the size distribution range of Pt-based IMCs with different alloying element compositions prepared in our work. **h** Pt L_3_-edge EXAFS spectra of PtCo/B/C, along with Pt/B/C, PtO_2_ and Pt foil in *R* space. **i** WT-EXAFS of the Pt L_3_-edge signal of PtCo/B/C. Normalized WT intensity was described by colorbar.

The strong interaction between Pt and B by Pt-B bonds reveals the origin of the outstanding anti-sintering ability. This allowed us to prepare a series of multicomponent IMCs nanoparticles through high-temperature annealing. As shown in Fig. 3a, the metal precursors with specific molar ratios were first impregnated into the as-prepared 20 wt%_Pt_ Pt/B/C. After drying, the powder precursors underwent an annealing treatment in a 5 vol% H_2_/Ar mixed gas at different temperatures to trigger the structural evolution from Pt to Pt-based multielement IMCs. High-temperature annealing provides the necessary activation energy for alloying and structural ordering, but it often leads to serious particle aggregation in the conventional scenario^16^. In our case, the particles are stably anchored on the B/C support and can maintain a small size after high-temperature annealing. The temperature-dependent ex-situ powder X-ray diffraction (XRD) experiments revealed the crystal structure evolution during the annealing process. As shown in Supplementary Figs. 19–21, after annealing at 1000 °C, superlattice peaks appeared at *ca*. 24° and 33°, corresponding to the (001) and (110) plane of an ordered face-centered tetragonal (FCT) structure. Additionally, the diffraction peaks shifted to higher angles, indicating successful diffusion of transition metals into the Pt lattice to form IMCs alloys^39^.

Accordingly, a series of carbon-supported Pt-based IMCs were synthesized, i.e., binary (PtCo/B/C, PtFe/B/C, PtCu/B/C, PtCo_3_/B/C, PtFe_3_/B/C, PtCu_3_/B/C), ternary (Pt_2_FeCo/B/C, Pt_2_FeNi/B/C, Pt_2_FeCu/B/C, Pt_2_CoNi/B/C, Pt_2_CoCu/B/C, Pt_2_NiCu/B/C), quaternary (Pt_3_FeCoNi/B/C, Pt_3_FeCoCu/B/C, Pt_3_CoNiCu/B/C), and quinary (Pt_4_FeCoNiCu/B/C). The XRD patterns in Fig. 3b and Supplementary Figs. 22–26 indicate that all the multicomponent IMCs appear the superlattice diffraction peaks of ordered FCT structure. HAADF-STEM images obtained from the aforementioned samples confirm the uniform distribution of both binary, ternary, quaternary, and quinary IMCs nanoparticles on the carbon support with narrow size distributions (Fig. 3c–f and Supplementary Fig. 27). The average size, as counted by nano measure, is concentrated around 4 nm, which is smaller than most carbon-supported intermetallic PtM reported in the literature (Fig. 3g)^16,40–47^. For comparison, a control experiment was conducted where PtCo and Pt_4_FeCoNiCu nanoparticles on KB support were synthesized via co-reduction at 1000 °C in a 5% H_2_/Ar atmosphere without the assistance of HB. TEM images reveal that the particles experienced severe coalescence, resulting in an average size exceeding 9 nm (Supplementary Figs. 28, 29).

The successful preparation of multielement IMCs with small sizes can still be attributed to the formation of Pt-B bond, effectively anchor and stabilize the nanoparticles even at high temperatures. This was also confirmed by Pt L_3_-edge EXAFS results, which indicated the presence of Pt-B bonds in PtCo/B/C samples annealed at 1000 °C in a 5% H_2_/Ar atmosphere, demonstrating the robustness of the Pt-B bond (Fig. 3h). Furthermore, the Pt EXAFS analysis confirmed a lattice contraction in PtCo/B/C. This compressive strain is known to enhance activity of Pt through tailoring the electronic state of Pt surface through a downshift of the *d*-band center^48^. The fitting results of the EXAFS revealed three scattering paths in PtCo/B/C: Pt-Pt, Pt-Co, and Pt-B (Supplementary Fig. 30 and Table 2). Notably, the Pt-Pt bond length in Pt/B/C (2.70 Å) was found to be shorter than that of Pt foil (2.76 Å), due to the incorporation of smaller Co atoms into the Pt crystal lattice. Moreover, the WT-EXAFS of the Pt L_3_-edge signal of PtCo/B/C indicates their intensity maximum consists of three intensity maxima (Fig. 3i) which is consistent with fitting results.

**Fig. 4:**
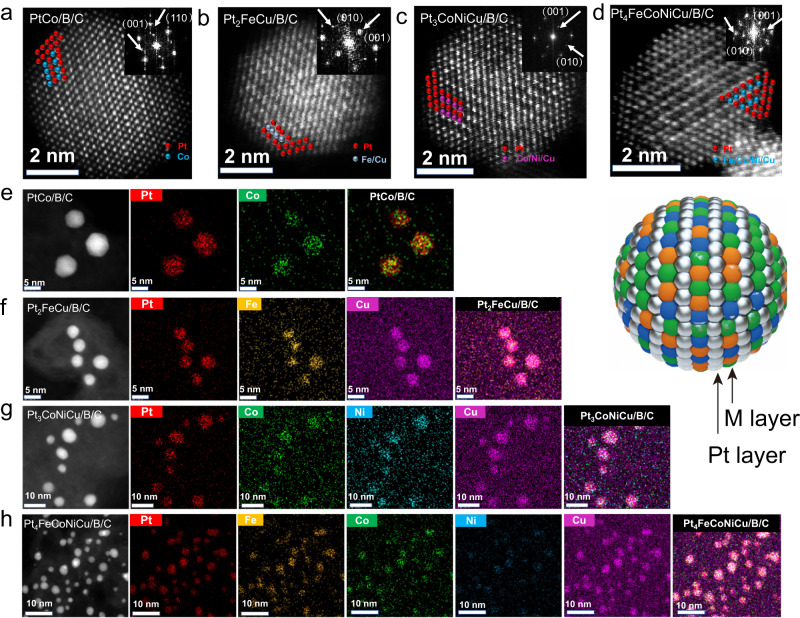
Atomic-resolution HAADF-STEM images and EDS elemental mappings of representative Pt-based IMCs. **a**–**d** Atomic-resolution HAADF-STEM images and FFT (inset) of PtCo/B/C, Pt_2_FeCu/B/C, Pt_3_CoNiCu/B/C, Pt_4_FeCoNiCu/B/C. **e**–**h** HAADF-STEM images and corresponding EDS elemental mappings of PtCo/B/C, Pt_2_FeCu/B/C, Pt_3_CoNiCu/B/C, Pt_4_FeCoNiCu/B/C.

The atomic-resolution HAADF-STEM images (Fig. 4) are shown to analyze the IMCs at an atomic scale. Since the intensity of the HAADF-STEM image is proportional to the atomic number Z, Pt atom columns are brighter than transition metal atom columns. Periodic square array structures are observed in all IMCs. Taking PtCo as an example, the lattice fringe of the core is 0.37 nm, corresponding to the superlattice (001) plane of L1_0_ ordered structure PtCo (0.370 nm, PDF card: no. 65-8969). The lattice fringe of the shell was 0.23 nm, corresponding to the (111) plane of Pt (0.230 nm, PDF card: No. 04-0802). The superlattice could also be monitored by the fast Fourier transform (FFT) which is derived from the atomic images (inset in Fig. 4a). Similar ordered tetragonal structures belonging to space group P4/mmm were observed in other samples, as shown in Fig. 4b–d and their respective insets. We can see that all samples are formed with a core containing an alternative layer of Pt and transition metal, surrounded by a thin Pt shell consisting of 2-3 atomic layers. The formation of this thin Pt shell can be attributed to acid treatment and post-annealing, where Pt with lower surface energy emerges as the shell. In addition, EDS mapping confirmed the homogeneity of Pt and other metallic elements in individual multimetallic particles (Fig. 4e–h), which matches well with the results obtained from the Inductively Coupled Plasma Mass Spectrometer (ICP-MS) (Supplementary Table 3).

#### Electrochemical ORR on different Pt-based IMCs

To assess the performance of the Pt-based multicomponent intermetallic PtM/B/C electrocatalysts, their electrocatalytic oxygen reduction reaction (ORR) capabilities were examined using the cyclic voltammetry (CV) and linear sweep voltammetry (LSV) in a 0.1 M perchloric acid solution (HClO_4_) at room temperature (around 25 °C). The measurements were conducted with a rotating disk electrode (RDE). For comparison, commercial Pt/C (JM, 20 wt%_Pt_) electrocatalysts were tested under identical conditions. The CV curves of all catalysts recorded in an N_2_-saturated electrolyte are presented in Supplementary Fig. 31a. The hydrogen underpotential deposition (HUPD) region in the CV curve was used to determine the electrochemically active surface area (ECSA). The ECSAs of PtCo, Pt_2_FeCu, Pt_3_CoNiCu, and Pt_4_FeCoNiCu were calculated to be 41.2, 47.1, 43.1, and 37.1 m^2^ g_Pt_^−1^, respectively. Although these values are smaller than that of commercial Pt/C (68.5 m^2^ g_Pt_^−1^), they are still larger than most of the reported Pt-based IMCs nanoparticles^39,41,49^, due to the high anti-sintering property, which enables the small NPs to be well retained during the high-temperature annealing.

**Fig. 5:**
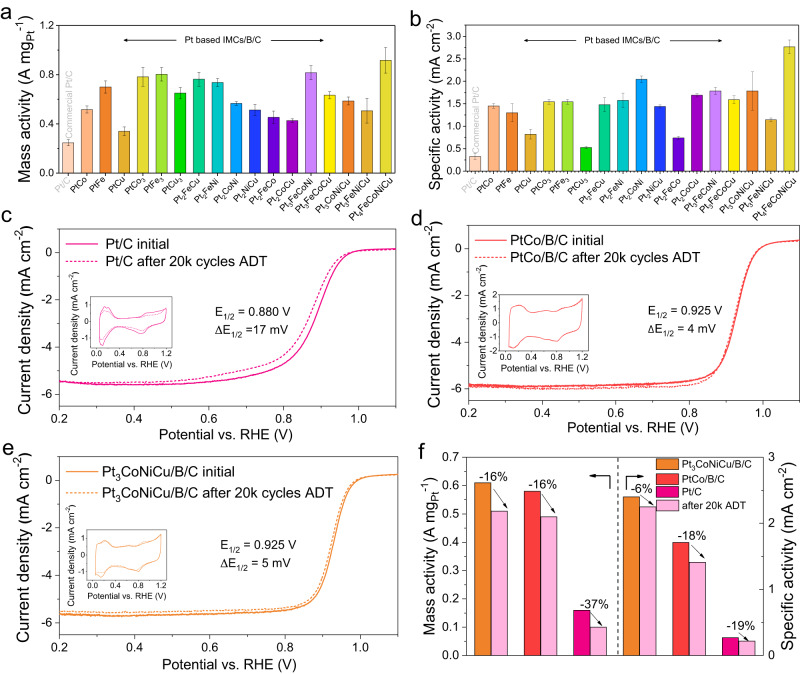
Electrocatalytic performance of Pt-based IMCs. **a**, **b** MA and SA at 0.9 V of the Pt-based IMCs/B/C catalysts, **c** CV and LSV curves of commercial Pt/C before and after 20k cycles ADT. **d** MAs and SAs of PtCo/B/C before and after 20k cycles ADT. **e** MAs and SAs of Pt_3_CoNiCu/B/C before and after 20k cycles ADT. **f** MAs and SAs of Pt_3_CoNiCu/B/C, PtCo/B/C, and Pt/C before and after 20k cycles ADT. RDE tests were measured by the three-electrode electrochemical cell in 0.1 M HClO_4_ diluted from 70% HClO_4_ (Sigma-Aldrich) by 18.25 MΩ de-ionized water. Commercial Pt/C was also tested for comparison. The error bars in (**a** to **b**) show the standard deviation in three independent electrochemical measurements for each sample.

Supplementary Fig. 31b show the LSV curves obtained in an O_2_-saturated electrolyte. It can be seen that all samples exhibit a positive shift of half-wave potentials (E_1/2_) compared to the commercial Pt/C (0.86 V), such as 0.920 V for PtCo, 0.925 V for Pt_2_FeCu, 0.921 V for Pt_3_CoNiCu, and 0.930 V for Pt_4_FeCoNiCu, indicating improved ORR activity. To get a direct insight into the enhancement in ORR activity of IMCs, their mass activity (MA) and specific activity (SA) at 0.9 V versus reversible hydrogen electrode (RHE) was obtained from the kinetic currents (calculating from the Koutecky -Levich equation) normalizing with ECSA and the mass loading of Pt, respectively. As shown in Fig. 5a, b, both the MA and SA values of the Pt-based IMCs catalysts significantly surpass those of commercial Pt/C (0.22 A mg _Pt_^−1^/0.27 mA cm _Pt_^−2^). Notably, the quinary Pt_4_FeCoNiCu exhibits remarkable MA and SA values of 1.0 A mg _Pt_^−1^ and 2.8 mA cm _Pt_^−2^, respectively, which are 4.5 and 10.4 times higher than those of commercial Pt/C, showing a large application potential in proton exchange membrane fuel cells (PEMFC).

In addition to electrocatalytic activity, the electrochemical stability during the ORR process is also significantly critical from the perspective of practical applications. All samples were subjected to continuous cycling by using the accelerated durability test (ADT) between 0.6 and 1.0 V vs. RHE potential in O_2_-saturated 0.1 M HClO_4_ at a scan rate of 100 mV s^−1^. We can see that two typical IMCs catalysts kept excellent activity after ADT, while commercial Pt/C descended sharply after 20k cycles as illustrated in Fig. 5c. To be specific, the PtCo/B/C catalyst showed only a minor negative shift of 4 mV in E_1/2_. Particularly, the binary PtCo/B/C catalyst displayed drops of 16% and 18% in MA and SA, respectively. Similarly, the quaternary Pt_3_CoNiCu IMCs demonstrated a negative shift of 5 mV in E_1/2_, accompanied by attenuations of 16% in MA and 6% in SA. In contrast, commercial Pt/C experienced a larger negative shift of 17 mV in E_1/2_, accompanied by a substantial attenuation of 37% in MA and 19% in SA, indicating poor durability. In addition, all IMCs catalysts exhibited negligible changes in the HUPD regions in CV curves, whereas commercial Pt/C showed a noticeable decrease in these regions (Fig. 5d–f and Supplementary Figs. 32, 33).

To obtain a visualized cognition of their difference in durability, the composition and structure of the catalysts after ADT were further investigated. Supplementary Figs. 34, 36, 38, and 40 show the HAADF-STEM of IMCs after 20k cycles. All IMCs dispersed well and exhibited almost constant average size. Only a slight increase in size distribution was observed compared to their originals (Fig. 3c–f). EDS mappings of a single selected nanoparticle reveal that the non-Pt elements still retained well after CV cycles and mixed homogeneously with Pt (Supplementary Figs. 35, 37, 39, and 41), revealing the Pt shell prevented the leaching of other metals^16,41^. For the commercial Pt/C, the NPs show a severe coalescence with the average size increasing from 2.5 nm to 4.6 nm, indicating the dramatic ripening during the harsh test condition (Supplementary Fig. 42).

## Discussion

In this manuscript, we have demonstrated a universal and robust strategy utilizing hydrogenated borophene to trigger the synthesis of Pt-based catalysts dispersed on various carbon and metal oxide-based supports. This strategy allows for loading amounts of Pt metal as high as 80 wt% and achieves fine sizes of ~2.5 nm. Through comprehensive experimental characterization, we have confirmed the structural stability of Pt/B and the formation of Pt-B bonds in Pt/B/C. The DFT calculation revealed that the strength of the Pt-B bond is more than 5 times stronger than Pt-C. The strong Pt-B bonding and robust B support endowed the catalyst with superior anti-sintering performance and offered a promising approach to prepare a series of multicomponent IMCs catalysts with sub-4 nm size through high-temperature annealing even at 1000 °C. All of the IMCs catalysts presented much-enhanced mass and specific activity than that of commercial Pt/C as well as promising durability, benefiting from the combined advantages of much small size and stable intermetallic structure. It is foreseeable that our findings offered a facile, general, and effective strategy to synthesize supported catalysts with exceptional stability and versatility, thus holding enormous potentiality and wide application prospects in the broad field of catalysis and energy conversion.

## Methods

### Materials

MgB_2_ (99.9%, 200 mesh) and ion-exchange resin (Amberlite IR120, H^+^ form) were obtained from Macklin Reagent. Methanol (AR, 99.9%) was purchased from Sinopharm Group Chemical Reagent. Graphitized carbon black (Ketjen black (ECP-600JD, KB)) was supplied by Cabot Carbon Ltd. Nafion solution (5 wt%_Nafion_) and HClO_4_ were purchased from Aldrich. H_2_PtCl_6_·6H_2_O were purchased from Kunming Institute of Precious Metals. Fe(NO_3_)_3_·9H_2_O, Co(NO_3_)_2_·6H_2_O, Ni(NO_3_)_2_·3H_2_O,Cu(NO_3_)_2_·6H_2_O were obtained from Macklin Reagent. Commercial Pt/C (20 wt%_Pt_) was purchased from Alfa Aesar Chemical Co Ltd. All the chemicals were analytical grade and used without further purification. Deionized water (18.25 MΩ/cm) was used throughout the experiments.

### Synthesis of HB methanol dispersion

HB methanol dispersion was prepared by the ion exchange method^23^. The detailed experimental procedures refer to our previous work^21^. Briefly, 60 mg MgB_2_ powder was added to 200 mL methanol and then ultrasonic treated in an ice bath for 30 min. After that, methanol suspension (100 mL) which contained ion exchange resin (30 g) was added to above suspension and stirred at 250 rpm under nitrogen for 3 days. Finally, the supernatant was evaporated at 343 K under nitrogen with an oil bath until the volume of liquid to 10 mL to obtain HB methanol dispersion. The content of HB is about 30 mg (in following experiments, to avoid the oxidation, no further drying was performed).

### Synthesis of Pt/B/support

10 mL as prepared HB solution and 0.4 g different support (such as KB, graphene, Al_2_O_3_, TiO_2_, CeO_2_, CNT) were dispersed in 200 mL methanol and stirred for 5 h, then 266 mg H_2_PtCl_6_·6H_2_O dissolved in 200 mL methanol was added to the mixed solution. After being stirred for three days, the precipitates were centrifugally collected and then washed with deionized water and dried in vacuum oven (labeled Pt/B/support).

### Synthesis of Pt-based IMCs

100 mg Pt/B/C powder was dissolved in 50 mL deionized water, then transition metal precursor was added with specific mole ratio. The mixture stirred in water bath at 60 °C until dried. The obtained powder was ground and annealed at a one or two-step high temperature (for one step, each temperature (600 and 900 °C) held 2 h. For two steps, 1000 °C for an hour and then annealed at 600 °C for 6 h) in 5% H_2_/Ar atmosphere with the heating rate of 5 °C min^−1^ and then cooled to room temperature naturally. Finally, the as-prepared alloys were treated in 0.1 M HClO_4_ at room temperature for 2 h, followed by an annealing treatment in 5% H_2_/Ar at 400 °C for 2 h. (labeled PtM/B/C).

### Synthesis of Pt-based alloys without B

For the PtCo sample, H_2_PtCl_6_·6H_2_O, Co(NO_3_)_2_·6H_2_O, and KB were dispersed into 50 mL DI-water (Pt/Co mole ratio 1:1 and Pt content to be 20 wt%) under ultrasonic for 2 h. After vacuum drying and grounding, the powder was subjected to heat treatment at 1000 °C for 1 h and then annealed at 600 °C for 6 h in a 5% H_2_/Ar atmosphere with a heating rate of 5 °C min^−1^. Finally, the powder was treated in 0.1 M HClO_4_ at room temperature for 2 h, followed by an annealing treatment in 5% H_2_/Ar at 400 °C for 2 h. The synthesis of Pt_4_FeCoNiCu was similar to that of PtCo, except for varying the added amount of transition metal salt and keeping the mole ratio of Pt/Fe/Co/Ni/Cu at 4:1:1:1:1.

### Characterizations

TEM, HRTEM, HAADF-STEM, XPS and elemental mapping analysis were taken on a JEOL JEM-F200 instrument equipped with energy-dispersive X-ray spectroscopy (EDX) at an accelerating voltage of 300 kV. HAADF-STEM images were acquired on a Cs-corrected JEM-ARM300F microscope equipped with dual spherical aberration correctors at an accelerating voltage of 300 kV. The Fusion TEM holder from Protochips was used to conduct in-situ heating process and realize the precise temperature control. The Pt/B/C sample was heated from 25 °C to 600 °C at a heating rate of 1 °C/s and kept at 600 °C for extra 10 min. The in-situ TEM videos were recorded on a JEOL JEM2100 at 200 kV. XRD patterns were acquired on a LabX XRD-6100 X-ray diffractometer by using Cu Kα radiation source (λ = 1.5406 A), operating at 40 kV and 30 mA. The mass loadings of Pt in the as-prepared catalysts were determined by inductively coupled plasma mass spectrometer with NexION 350D and thermogravimetry with TGMETTLER TOLEDO TGA/DSC3^+^. XPS was carried out on a Thermo Fisher Scientific ESCALAB Xi^+^ spectrometer with an Al Kα radiator. For the HB/C sample, HB is firstly mixed with ketjen carbon black in methanol via thoroughly stirring for 5 h to form the HB/C compound, then the compound was evaporated on an oil bath at 343 K. The powder was dried in vacuum oven overnight and pressed on the tape for XPS test. The binding energy of the C 1 *s* peak (284 eV) was used as a standard to calibrate the binding energies of other elements. For the Pt/B/C sample, the powder was pressed on the tape for XPS test. Pt L-edge analysis was performed with Si (111) crystal monochromators at the BL14W1 beamlines at the Shanghai Synchrotron Radiation Facility (SSRF) (Shanghai, China). Before the analysis at the beamline, samples were pressed into thin sheets with 1 cm in diameter and sealed using Kapton tape film. The XAFS spectra were recorded at room temperature using a 4-channel Silicon Drift Detector (SDD) Bruker 5040, operated at 3.5 GeV with injection currents of 140–210 mA. To obtain a much better signal-to-noise ratio, the integration time was extended to 100 ms in the characterization of experimental samples, which is 50 ms in Pt reference samples (Pt foil and PtO_2_). The standard sample (Pt foil and commercial Pt/C) were first reduced at 250 °C in H_2_ atomosphere for 30 min and then tested in a He flow.We used IFEFFIT software (Athena) to correct the background signal, calibrate the energy scale and normalize the intensity of shock wave. Reliable parameters for the high Z (Pt, Co) and low Z (B, O) contributions were determined by multiple-shell fitting in R space with application of k^3^ and k^1^ weightings in the Fourier transformations by software (Artemis)^50^.

### Electrochemical measurement

All ORR electrochemical tests were conducted on the CHI 760 equipped with a three-electrode system which used a glassy carbon electrode (area 0.196 cm^2^) as the working electrode and saturated Ag/AgCl and a platinum foil as a reference and counter electrodes respectively. The reference electrode potential was calibrated to the reversible hydrogen electrode (RHE) potentials under hydrogen-saturated 0.1 M HClO_4_ solution before the test. Catalyst ink was prepared by mixing 1 mg catalyst with 200 μL of ultrapure water, 800 μL of isopropanol, and 10 μL of Nafion solution (5 wt%_Nafion_) and ultrasonicating for 30 min. Then, 20 μL the catalyst ink was dropped onto the glassy carbon rotating disk electrode for electrochemical measurements. All the data has been corrected with *iR* (Ohmic) drop. In addition, we replaced the electrolyte immediately after testing each electrode to avoid being contaminated. All the mearsurements were performed at room temperature (25 °C).

### DFT calculations

In this paper, we employed first-principles study based on the spin-polarized density functional theory (DFT) within the projector augmented wave method^51^, as implemented in Vienna ab initio simulation package (VASP software version: vasp.5.4.1) to examine the adsorption stability of Pt clusters on graphene and borophene substrates. The electron exchange-correlation interactions were described using the generalized gradient approximation (GGA) with the functional of Perdew-Burke-Ernzerhof (PBE)^52^. The cut-off of plane-wave kinetic energy and the convergence of total energy were set to 450 eV and 10^−5^ eV. Using a conjugate gradient algorithm, structural relaxations were performed by computing the Hellmann-Feynman forces within a force convergence of 0.01 eV/Å^53^. All studied models were located in the x-y plane with a large supercell. Supercells containing 4 × 3 and 6 × 6 primitive cells were adopted for borophene and graphene. In order to approximate the Brillouin zone integrations, 5 × 5 × 1 k-point meshes with Gamma centered grid were used. Due to the periodic boundary conditions, a vacuum region of at least 10 Å was applied along the z-axis for eliminating the interactions between neighbor layers. The adsorption energy was calculated according to the following equation:

$${\Delta {{{{{\rm{G}}}}}}}_{{{{{{\rm{ad}}}}}}}={\Delta {{{{{\rm{G}}}}}}}_{{{{{{\rm{Pt}}}}}}/{{{{{\rm{substrate}}}}}}}-({\Delta {{{{{\rm{G}}}}}}}_{{{{{{\rm{Pt}}}}}}}-{\Delta {{{{{\rm{G}}}}}}}_{{{{{{\rm{subtrate}}}}}}})$$

where ${\Delta {{{{{\rm{G}}}}}}}_{{{{{{\rm{Pt}}}}}}/{{{{{\rm{substrate}}}}}}}$ and ${\Delta {{{{{\rm{G}}}}}}}_{{{{{{\rm{subtrate}}}}}}}$ are the free energies of graphene or borophene with and without Pt cluster adsorption, and ${\Delta {{{{{\rm{G}}}}}}}_{{{{{{\rm{Pt}}}}}}}$ is energy of Pt cluster. Using adiabatic movements of the corresponding atom, escape energy of one selected Pt atom from the Pt_13_ cluster on graphene and borophene is derived from the variation of total energy.

### Supplementary information

Supplementary Information

Peer Review

### Source data

Source Data

## Data Availability

All data supporting the findings of this study are available in the main text or Supplementary Information. Source data are provided with this paper.
