# Author Correction: Hydrogenated borophene enabled synthesis of multielement intermetallic catalysts

**DOI:** 10.1038/s41467-024-48081-y

**Published:** 2024-04-29

**Authors:** Xiaoxiao Zeng, Yudan Jing, Saisai Gao, Wencong Zhang, Yang Zhang, Hanwen Liu, Chao Liang, Chenchen Ji, Yi Rao, Jianbo Wu, Bin Wang, Yonggang Yao, Shengchun Yang

**Affiliations:** 1 MOE Key Laboratory for Non-equilibrium Synthesis and Modulation of Condensed Matter, School of Physics, Xi’an Jiaotong University, Xi’an, 710049 PR China; 2 National Innovation Platform (Center) for Industry-Education Integration of Energy Storage Technology, Xi’an Jiaotong University, Xi’an, 710049 PR China; 3 Shaanxi Coal Chemical Industry Technology Research Institute Co., Ltd, Xi’an, 710100 PR China; 4 State Key Laboratory of Metal Matrix Composites, School of Materials Science and Engineering, Shanghai Jiao Tong University, Shanghai, 200240 PR China; 5 Hydrogen Science Research Center, Zhangjiang Institute for Advanced Study, Shanghai Jiao Tong University, Shanghai, 200240 PR China; 6 State Key Laboratory of Materials Processing and Die & Mould Technology, School of Materials Science and Engineering, Huazhong University of Science and Technology, Wuhan, 430074 PR China; 7 State Key Laboratory of Chemistry and Utilization of Carbon Based Energy Resources, School of Chemical Engineering and Technology, Xinjiang University, Urumqi, 830017 PR China

**Keywords:** Fuel cells, Fuel cells, Electrocatalysis

Correction to: *Nature Communications* 10.1038/s41467-023-43294-z, published online 16 November 2023

In this article the wrong figures appeared as Figs. 1c and 1d; the figures should have appeared as shown below.


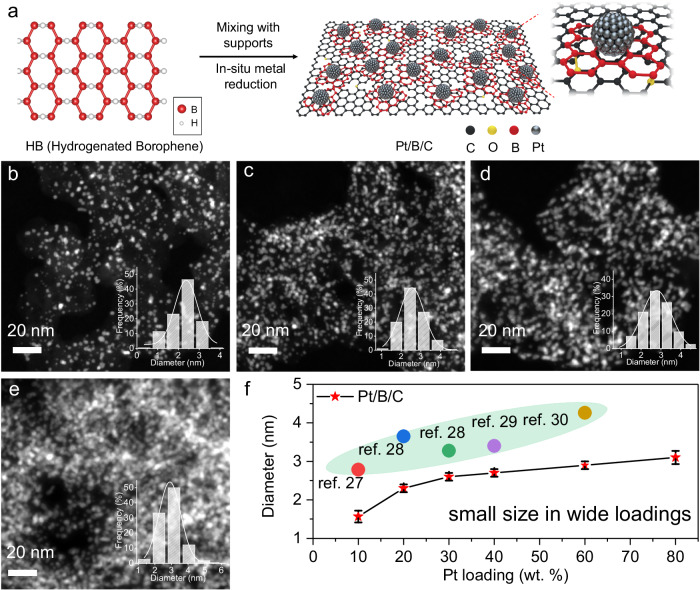


The original article has been corrected.

