# Supplementary material for: Hydrogenated borophene enabled synthesis of multielement intermetallic catalysts
Source: Nat Commun. 2023 Nov 16;14:7414. doi: 10.1038/s41467-023-43294-z (PMC10654666; doi:10.1038/s41467-023-43294-z)
Supplement: Supplementary file 1 — Supplementary Information [file 41467_2023_43294_MOESM1_ESM.docx]

**Supplementary** **Information for**

**Hydrogenated borophene enabled synthesis of multielement intermetallic catalysts**

Xiaoxiao Zeng^1,2#^, Yudan Jing^1,3,#^, Saisai Gao^1,3,#^, Wencong Zhang^4,5,#^, Yang Zhang^1,2^, Hanwen Liu^6^, Chao Liang^1,2^, Chenchen Ji^7^, Yi Rao^6^, Jianbo Wu^4,5^, Bin Wang^1,2,*^, Yonggang Yao^6,*^ and Shengchun Yang^1,2,*^

^1^ MOE Key Laboratory for Non-equilibrium Synthesis and Modulation of Condensed Matter, School of Physics, Xi’an Jiaotong University, Xi'an 710049, PR China.

^2^ National Innovation Platform (Center) for Industry-Education Integration of Energy Storage Technology, Xi’an Jiaotong University, Xi'an 710049, PR China

^3^ Shaanxi Coal Chemical Industry Technology Research Institute Co., Ltd., Xi’an 710100, PR China.

^4^ State Key Laboratory of Metal Matrix Composites, School of Materials Science and Engineering, Shanghai Jiao Tong University, Shanghai 200240, PR China.

^5^ Hydrogen Science Research Center, Zhangjiang Institute for Advanced Study, Shanghai Jiao Tong University, Shanghai 200240, PR China.

^6^ State Key Laboratory of Materials Processing and Die & Mould Technology, School of Materials Science and Engineering, Huazhong University of Science and Technology, Wuhan, 430074, PR China.

^7^State Key Laboratory of Chemistry and Utilization of Carbon Based Energy Resources, School of Chemical Engineering and Technology, Xinjiang University, Urumqi 830017, PR China.

^*^ Corresponding authors, E-mail: bin_wang@xjtu.edu.cn(B. W.); yaoyg@hust.edu.cn(Y. Y.); ysch1209@mail.xjtu.edu.cn(S. Y.).

^#^ These authors contributed equally to this work.

**This Supplementary materials contains the following information:**

- **Supplementary Figure 1-42**
- **Supplementary Table 1-3**
- **Supplementary Methods**


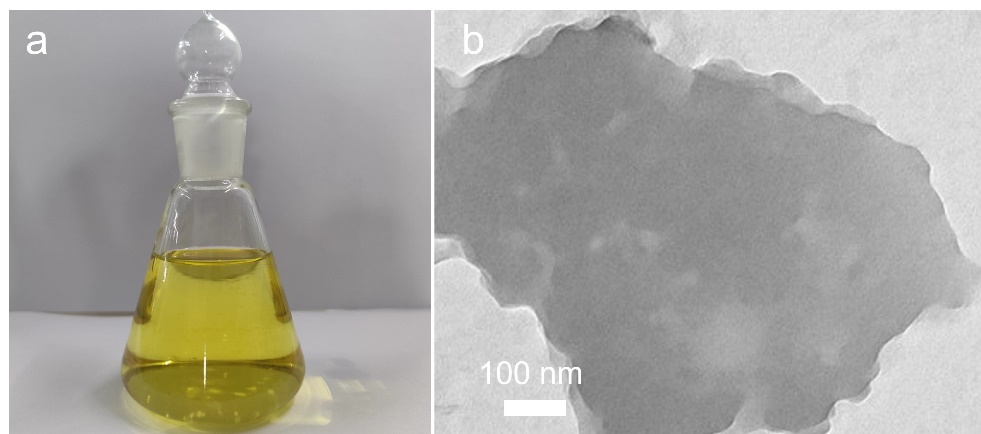


**Supplementary Fig. 1 | The as-prepared HB.** **a,** HB methanol dispersion. **b,** TEM image of HB.


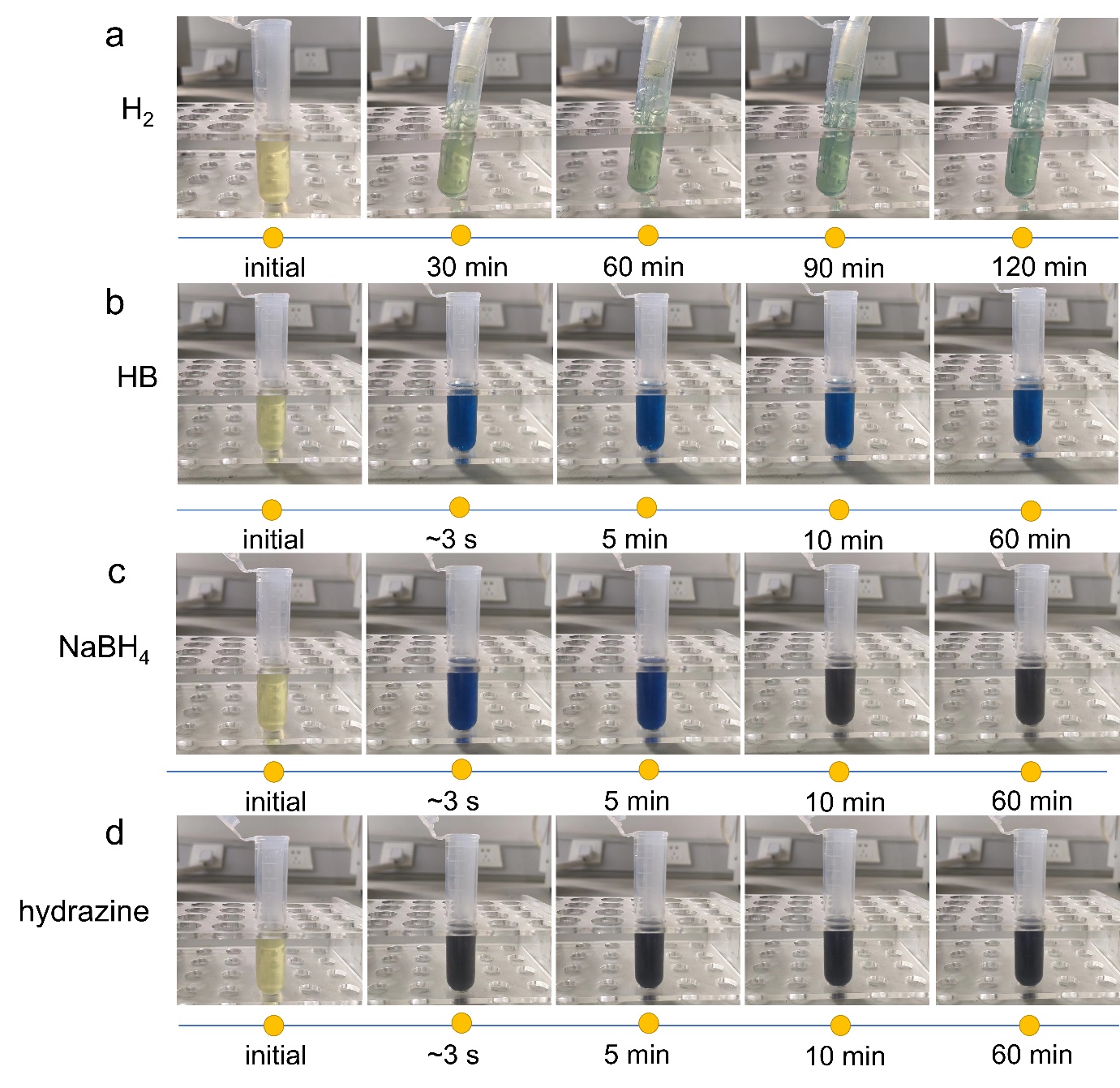


**Supplementary Fig. 2 |** **Color changes of the solution containing K_3_[Fe(CN)_6_] and Fe^3+^ in a molar ratio of 1:1 over time when added different reducing agents. a**, H_2_. **b,** HB. **c** NaBH_4_. **d.** hydrazine, respectively.


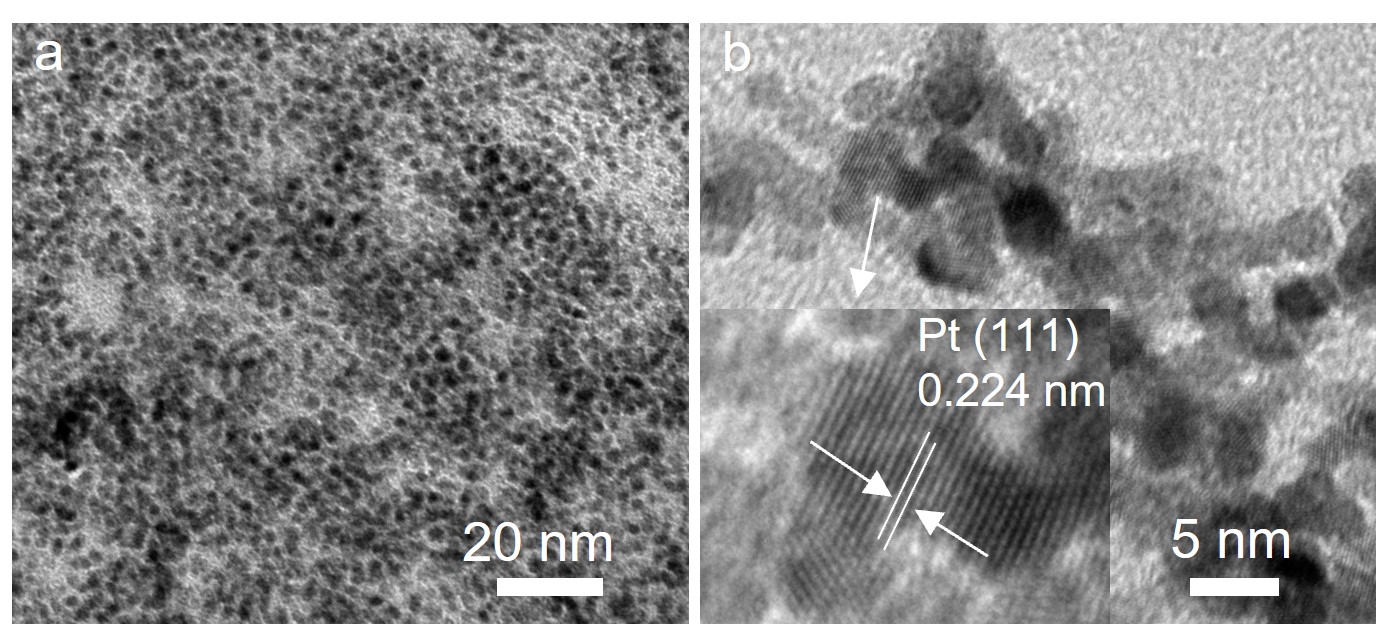


**Supplementary Fig. 3 |** **TEM image**s. **a-b,** TEM image and high-resolution TEM of Pt/B. Synthesis method: 10 mL HB solution and 266 mg H_2_PtCl_6_·6H_2_O dissolved in 200 ml methanol were mixed. After being stirred for three days, the Pt/B was obtained. Inset: magnified HRTEM image of a single particle.


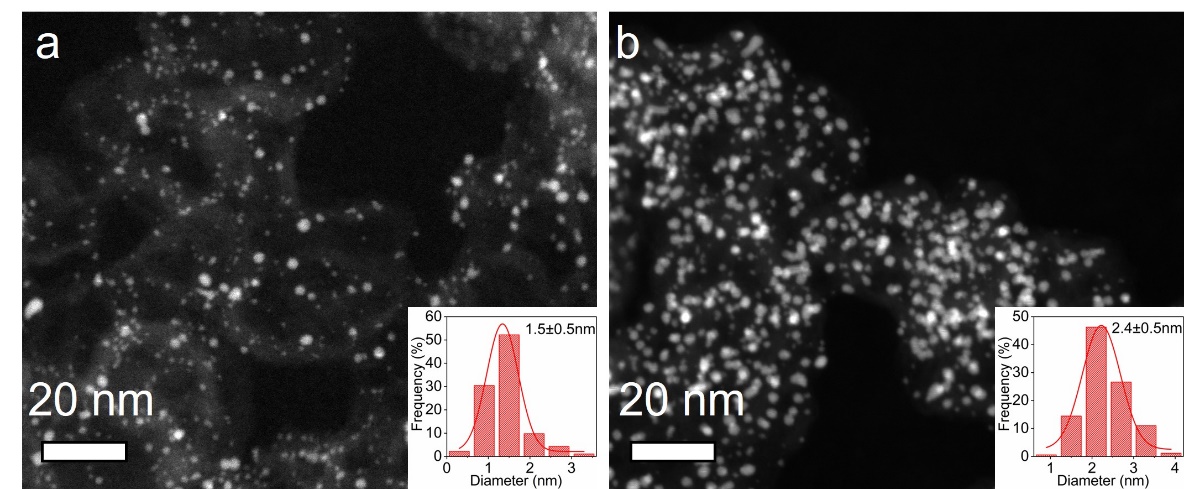


**Supplementary Fig. 4 |** **HAADF-STEM images. a-b,** HAADF-STEM images of Pt/B/C with Pt loading amounts of 10, and 30 wt%, respectively. The inserted histograms are the statistics of particle size distribution of corresponding sample.


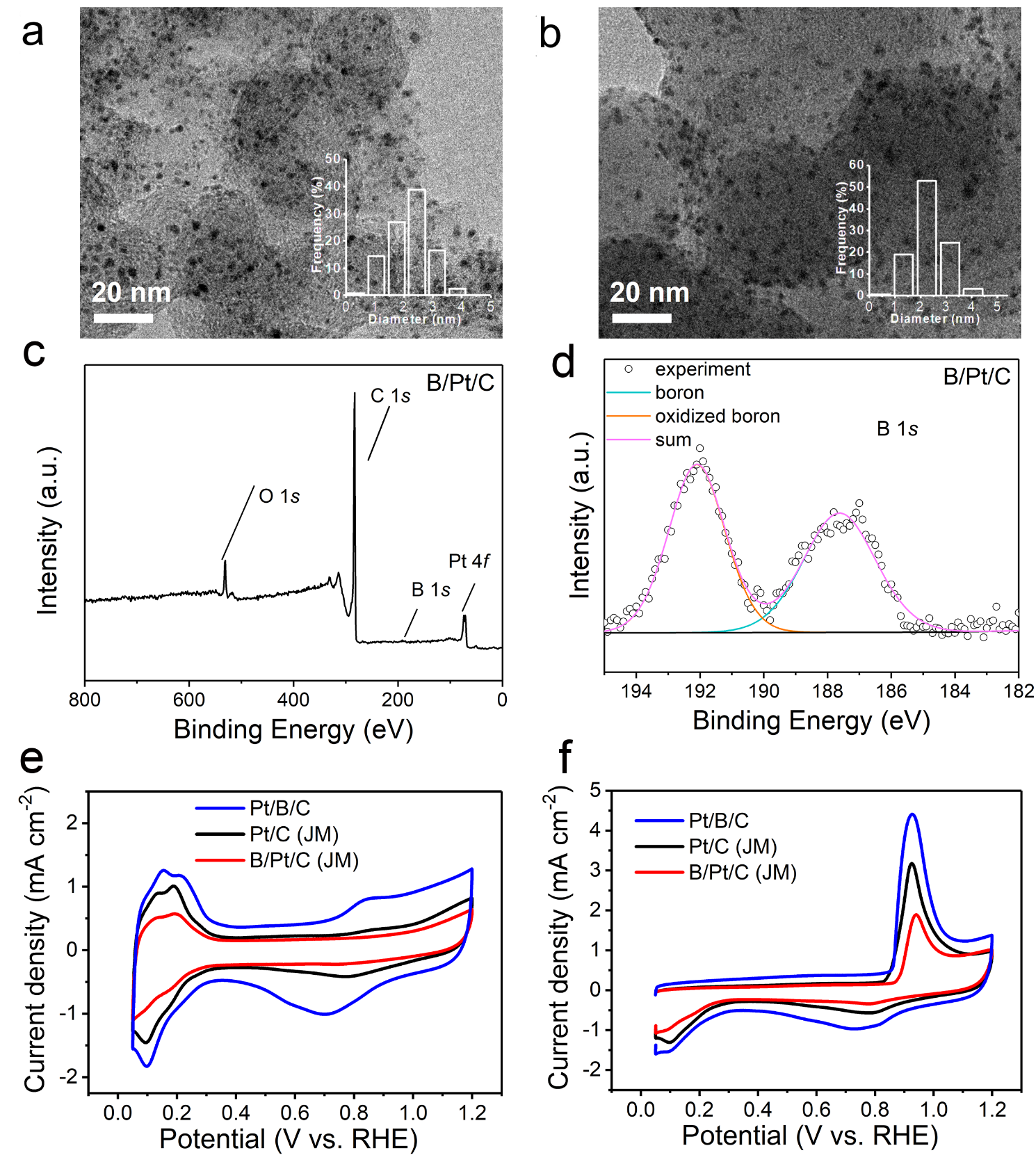


**Supplementary Fig. 5 |** **TEM and CV characterization of samples.** **a-b,** TEM of Pt/B/C and commercial Pt/C (JM) catalyst. The inserted histograms are the statistics of particle size distribution of corresponding sample. **c-d,** survey spetra XPS and B 1*s* XPS of B/Pt/C. **e-f,** CV curves and CO-stripping of Pt/B/C (as-prepared), commercial Pt/C (JM) and B/Pt/C (JM).


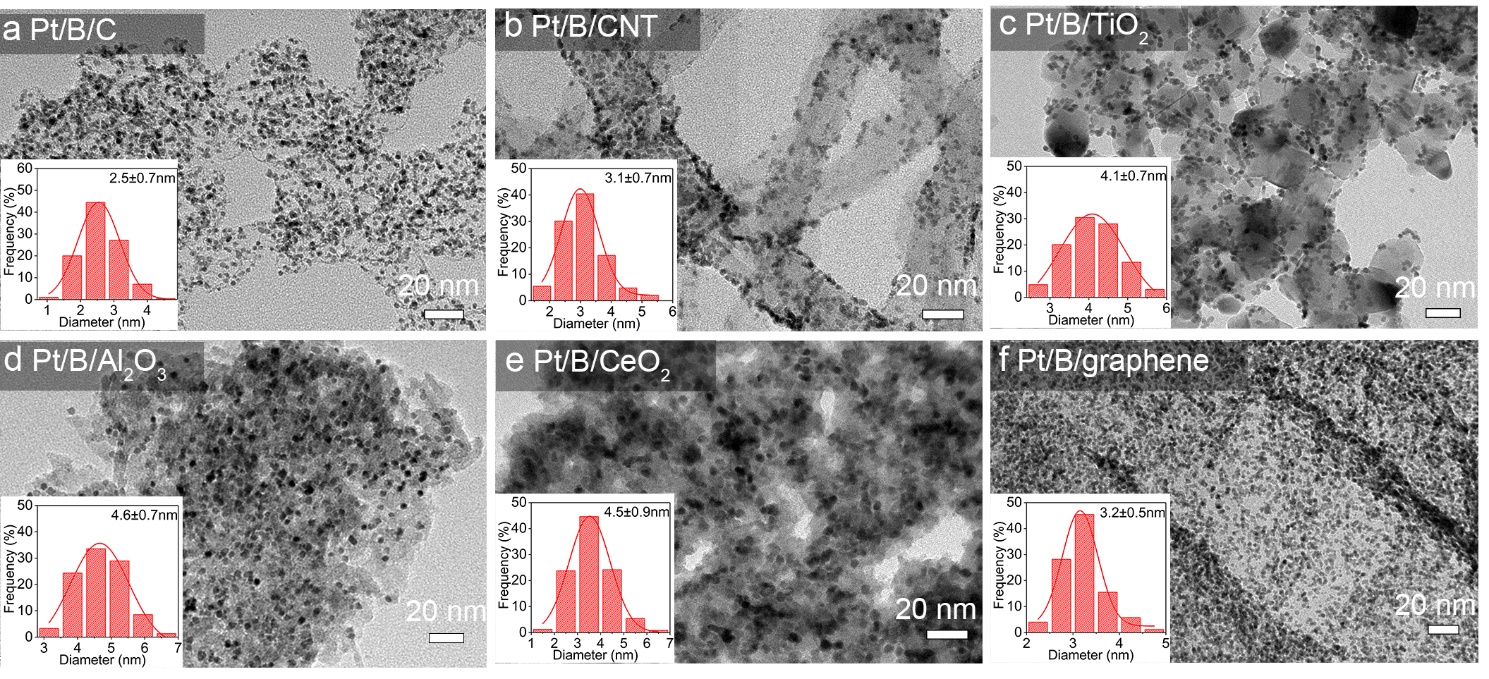


**Supplementary Fig. 6 |** **TEM characterizations of sample with different supports.** **a,** Pt/B/C. **b,** Pt/B/CNT. **c,** Pt/B/TiO_2_. **d,** Pt/B/Al_2_O_3_. **e,** Pt/B/CeO_2_. **f,** Pt/B/graphene. The loading amount of Pt is 40 wt%, 40 wt%, 30 wt%, 30 wt%, 30 wt%, 80 wt% for **a**, **b**, **c**, **d**, **e** and **f**, respectively. The inserted histograms are the statistics of particle size distribution of corresponding sample.


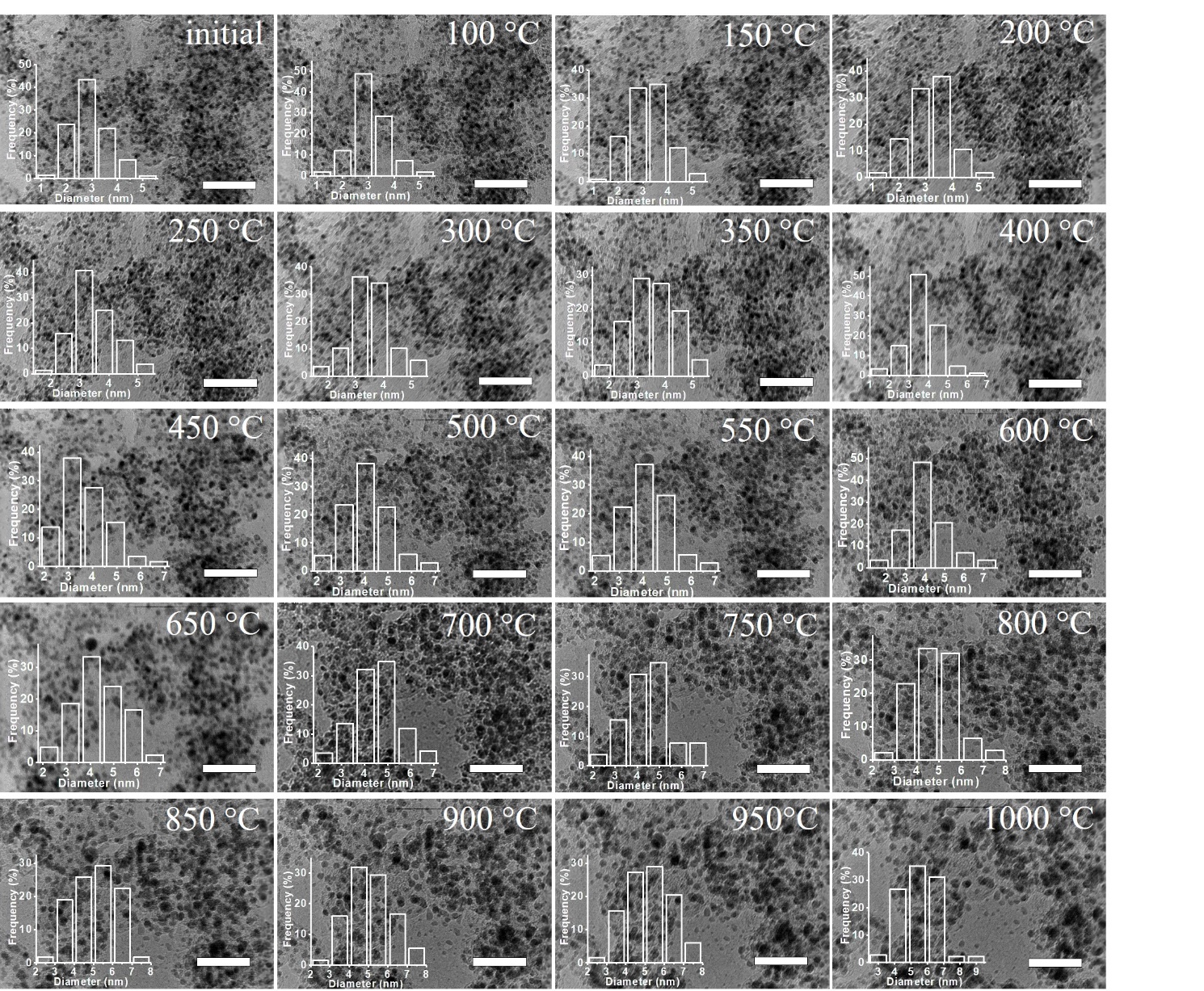


**Supplementary Fig. 7 | In-situ TEM images of Pt/B/C from room temperature to 1000 ℃.** The loading amount of Pt is 60 wt% and the scale bar is 40 nm. The inserted histograms are the statistics of particle size distribution of corresponding temperature.


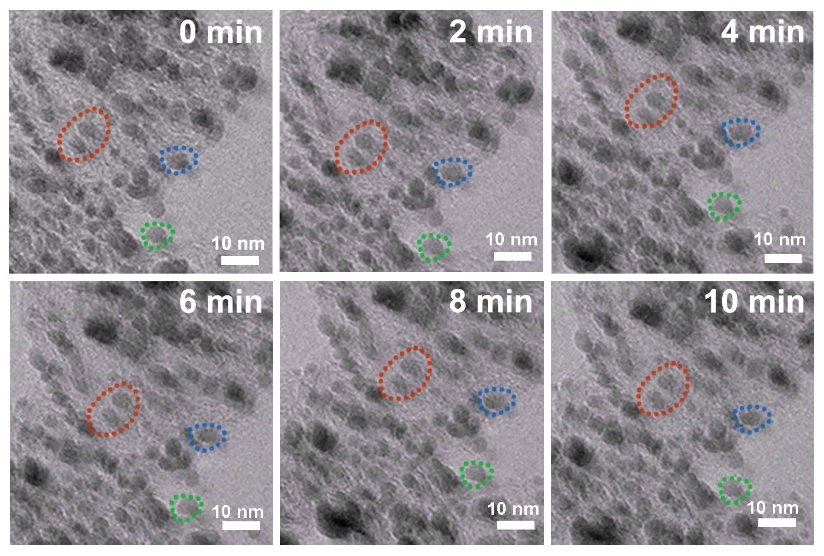


**Supplementary Fig. 8 | Time sequential TEM images of Pt/B/C kept at 600 ℃ for 10 min.** The red shows that the distance between the two particles was not changed over time. The bule and green circles present that the shape of two single nanoparticles was not changed over time.


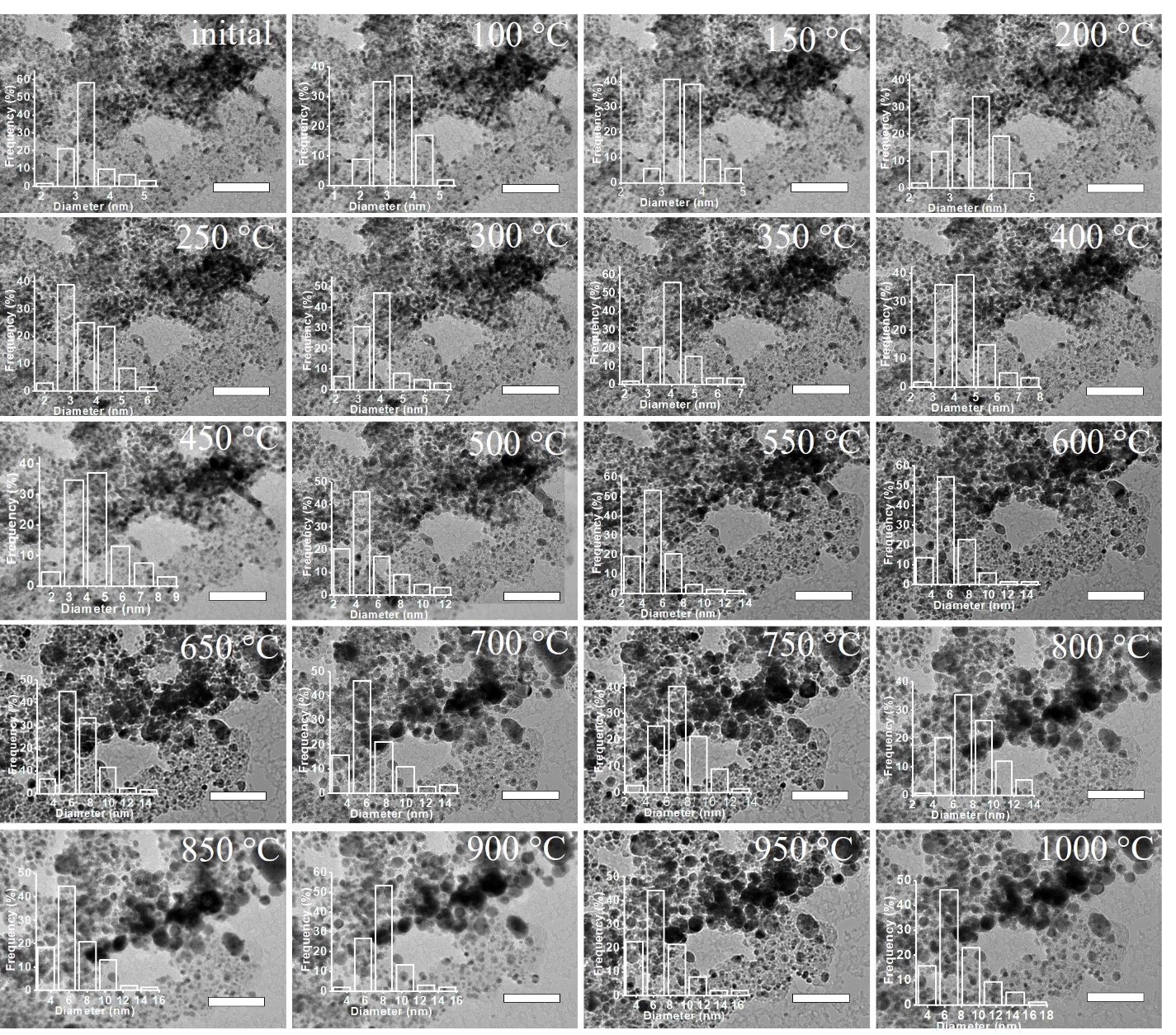


**Supplementary Fig. 9 |** **In-situ TEM images of commercial Pt/C from room temperature to 1000 ℃.** The loading amount of Pt is 60 wt% and the scale bar is 50 nm. The inserted histograms are the statistics of particle size distribution of corresponding temperature.


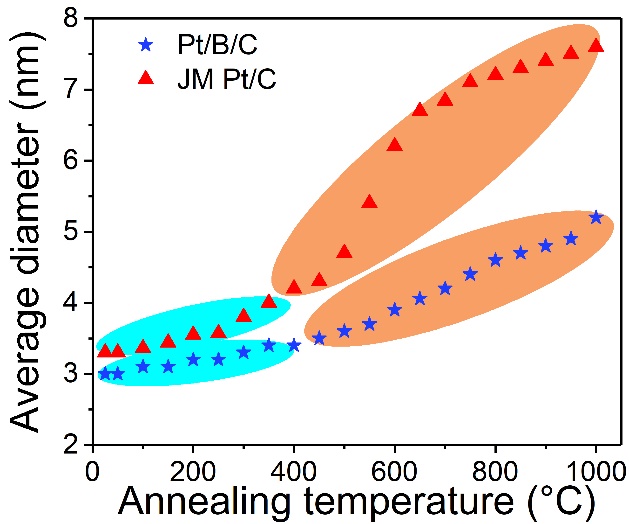


**Supplementary Fig. 10 |** The changes in average size of Pt/B/C and commercial Pt/C catalysts with the annealing temperature increasing from room temperature to 1000 ℃. The sizes were recorded from the in-situ TEM images of Pt/B/C and commercial Pt/C.


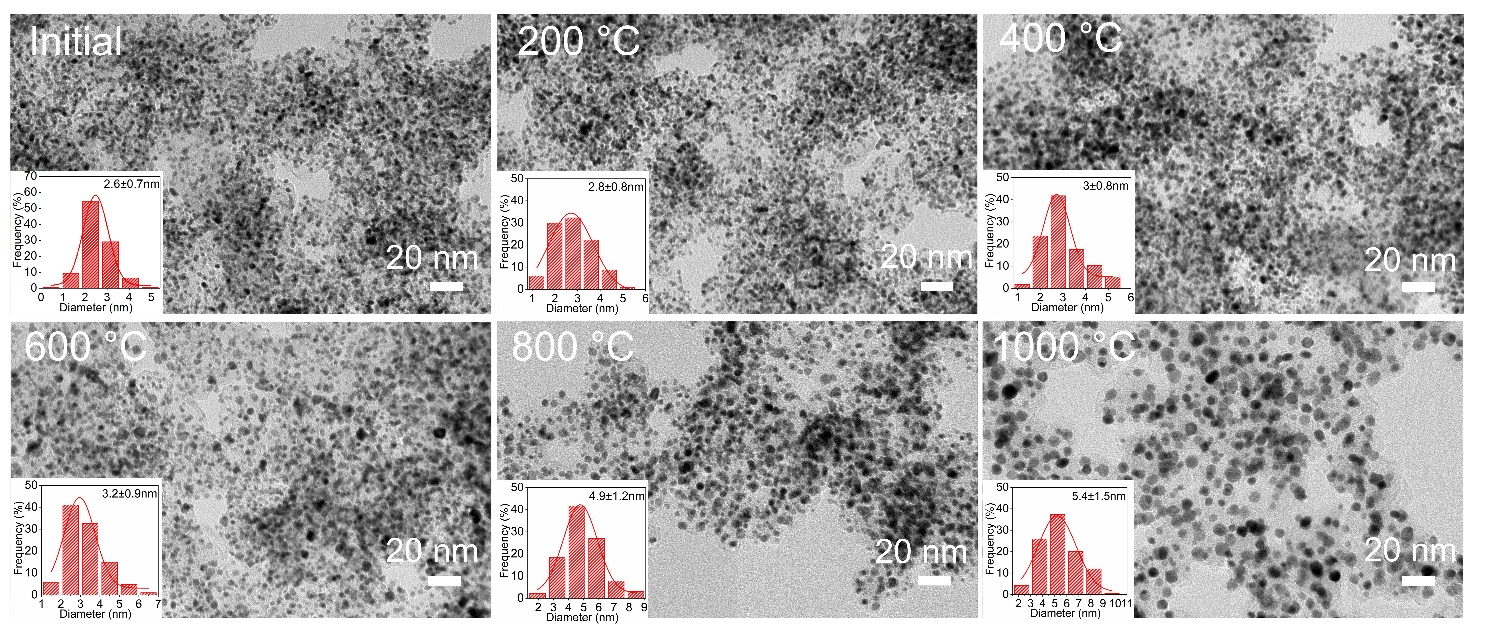


**Supplementary Fig. 11 |** **Ex-situ TEM images of Pt/B/C from room temperature to 1000 ℃.** The heating rate is 5 °C min^-1^and annealing time for each temperature is 2 h. The inserted histograms are the statistics of particle size distribution of corresponding temperature.


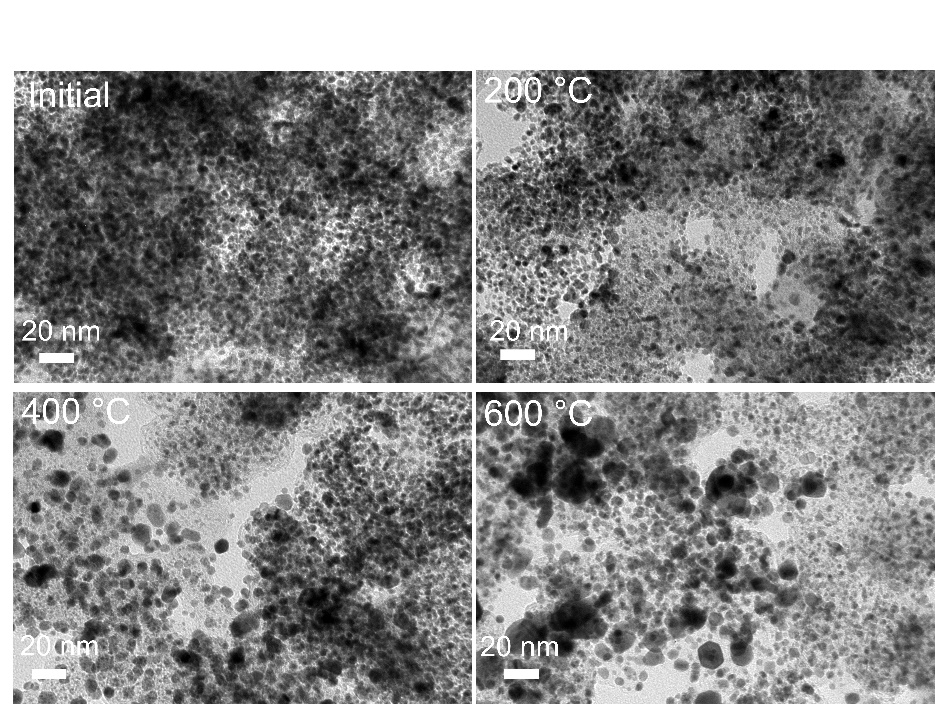


**Supplementary Fig. 12 |** Ex-situ TEM images of commercial Pt/C from room temperature to 600 ℃. The annealing time for each temperature is 2 h.


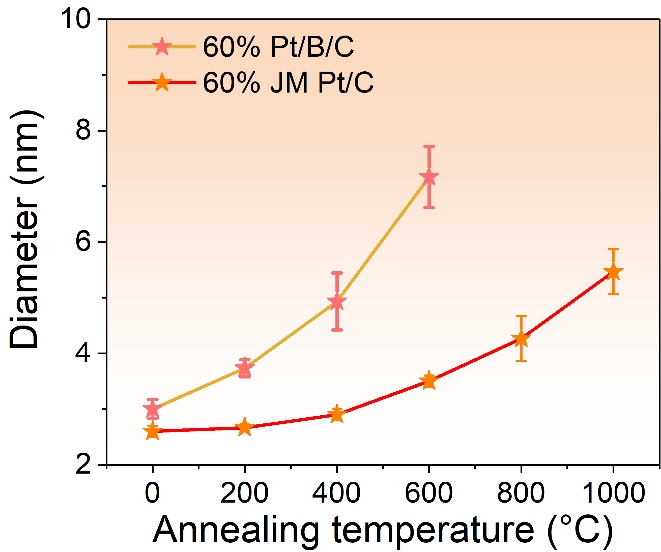


**Supplementary Fig. 13 |** The changes in average size of Pt/B/C and commercial Pt/C catalysts with the annealing temperature increasing from room temperature to 1000 ℃. The sizes were recorded from the ex-situ TEM images of Pt/B/C and commercial Pt/C.


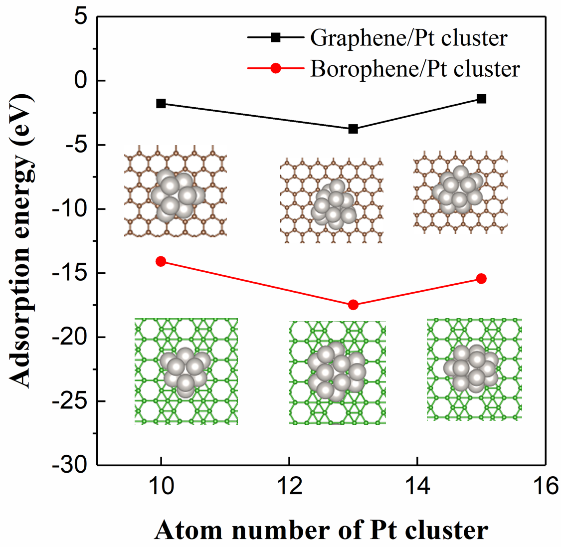


**Supplementary Fig. 14 |** Adsorption model (top view) of Pt clusters containing 10, 13 and 15 atoms on graphene and borophene, respectively. The silver, green, and brown balls represent Pt, B, C atoms respectively.





**Supplementary Fig. 15 |** The adsorption Gibbs free energy changes (ΔG) with the number of Pt atoms for the systems of Pt/borophene and Pt/graphene, respectively.


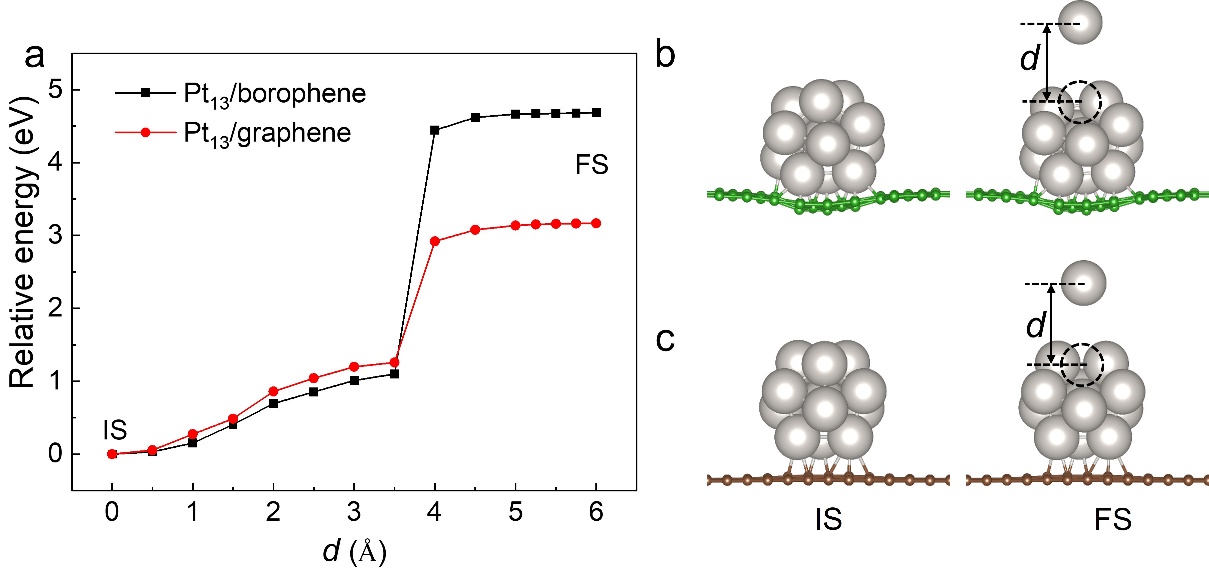


**Supplementary Fig. 16 | Theoretical investigations of Pt atom escaping from Pt13 cluster loaded on graphene and borophene. a,** Escape energy of one selected atom (top site) from the Pt13 cluster on graphene and borophene; **b** and c, the diagram of the escaping Pt atom from Pt13 cluster loaded on graphene and borophene, respectively. The silver, green, and brown balls represent Pt, B, C atoms respectively.


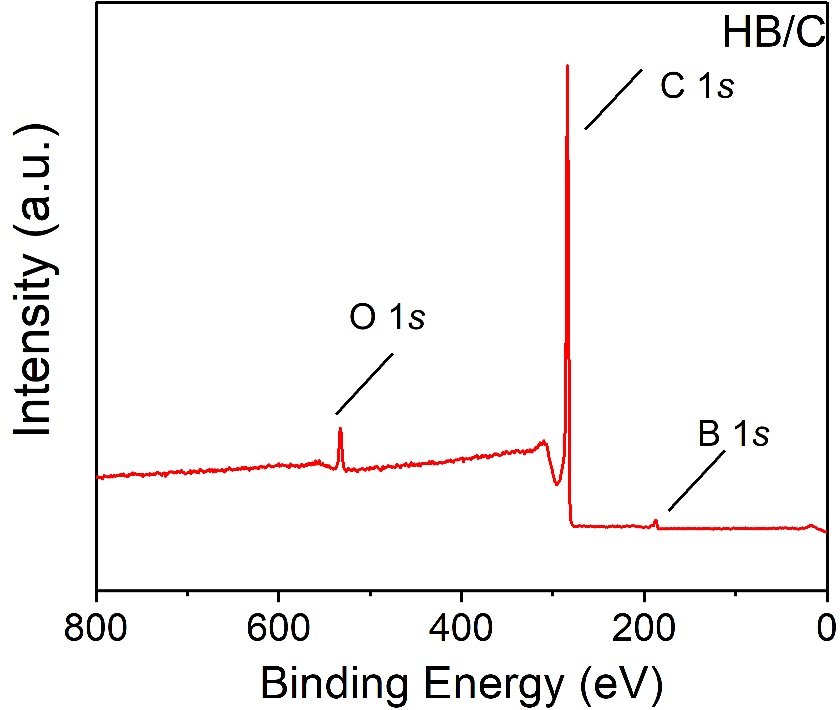


**Supplementary Fig. 17 |** Survey spectra of HB/C.


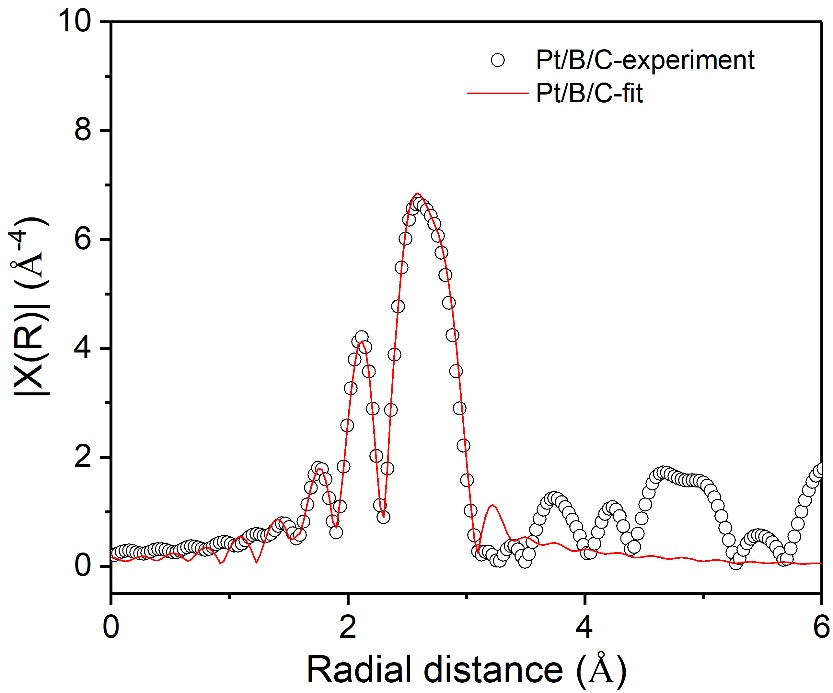


**Supplementary Fig. 18 |** EXAFS fitting spectra at Pt L_3_-edge of Pt/B/C.


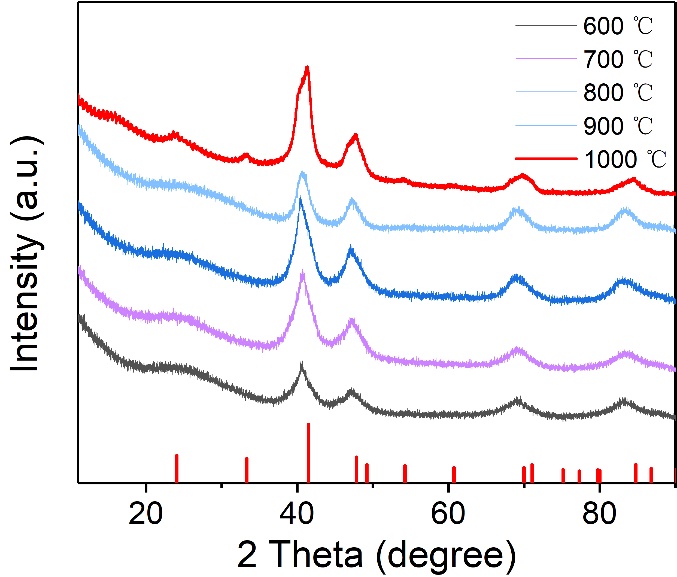


**Supplementary Fig. 19 |** Temperature evolution ex-situ XRD patterns of PtCo catalyst during the annealing process.


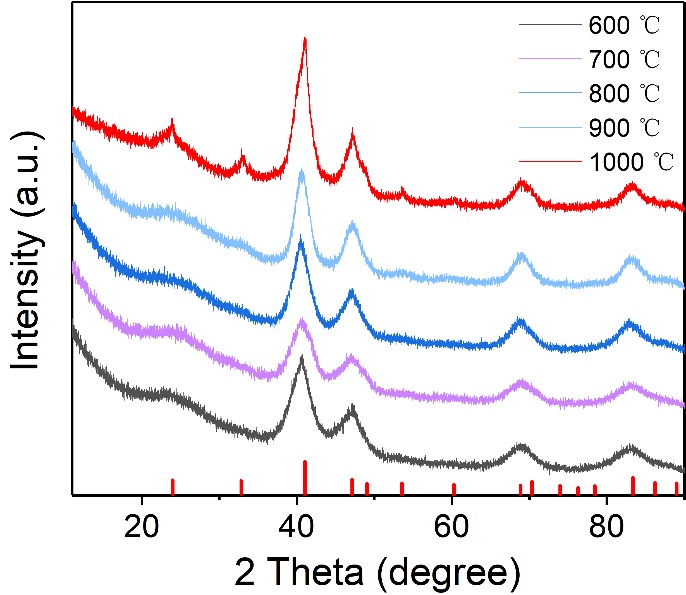


**Supplementary Fig. 20 |** Temperature evolution ex-situ XRD patterns of PtFe catalyst during the annealing process.


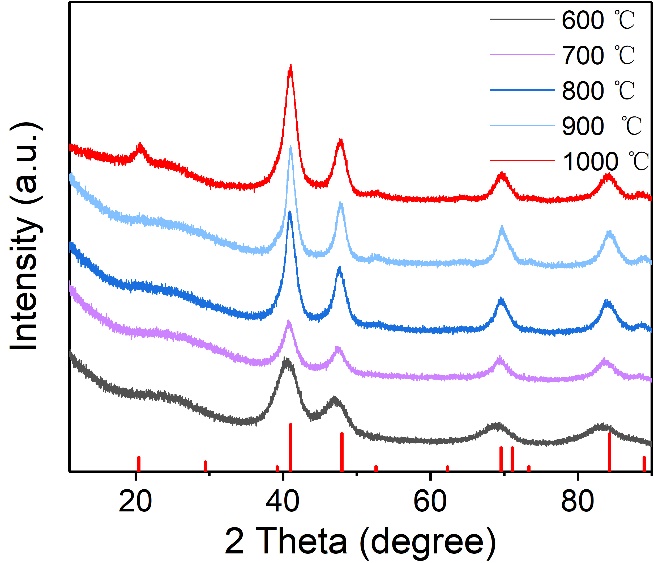


**Supplementary Fig. 21 |** Temperature evolution ex-situ XRD patterns of PtCu catalyst during the annealing process.


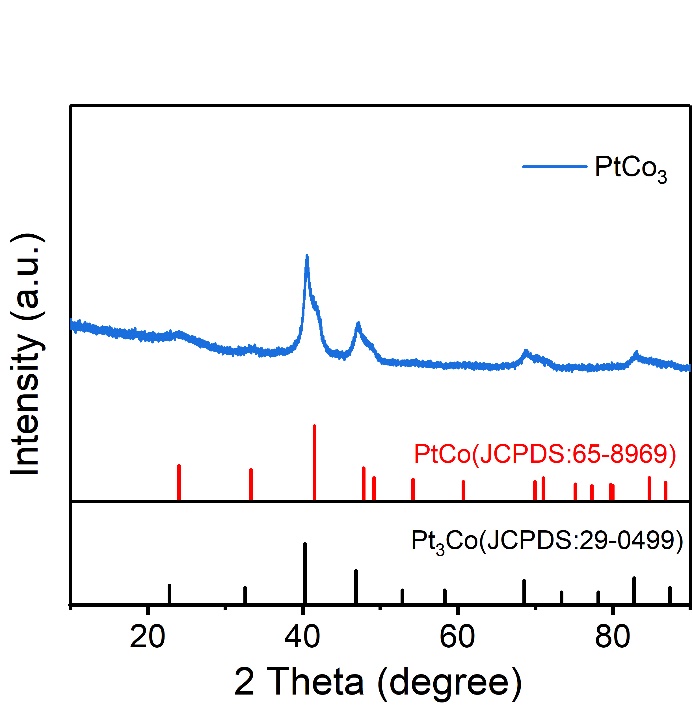


**Supplementary Fig. 22 |** XRD patterns of PtCo_3_, showing a little phase separation.There is no PDF for PtCo_3_.


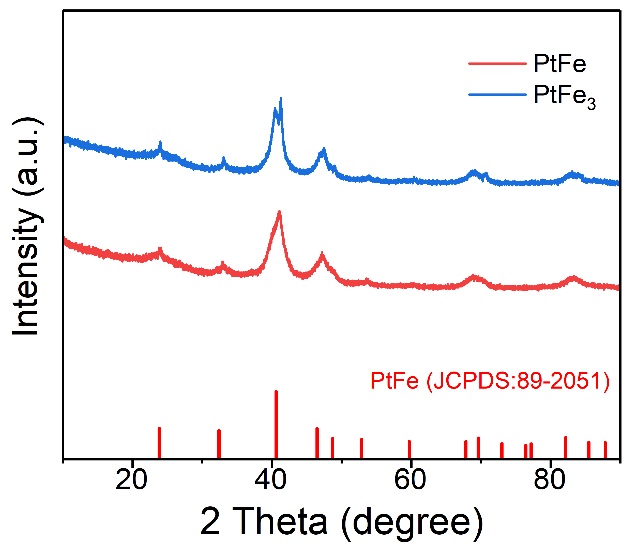


**Supplementary Fig. 23 |** XRD patterns of PtFe and PtFe_3_. There is no PDF for PtFe_3_.


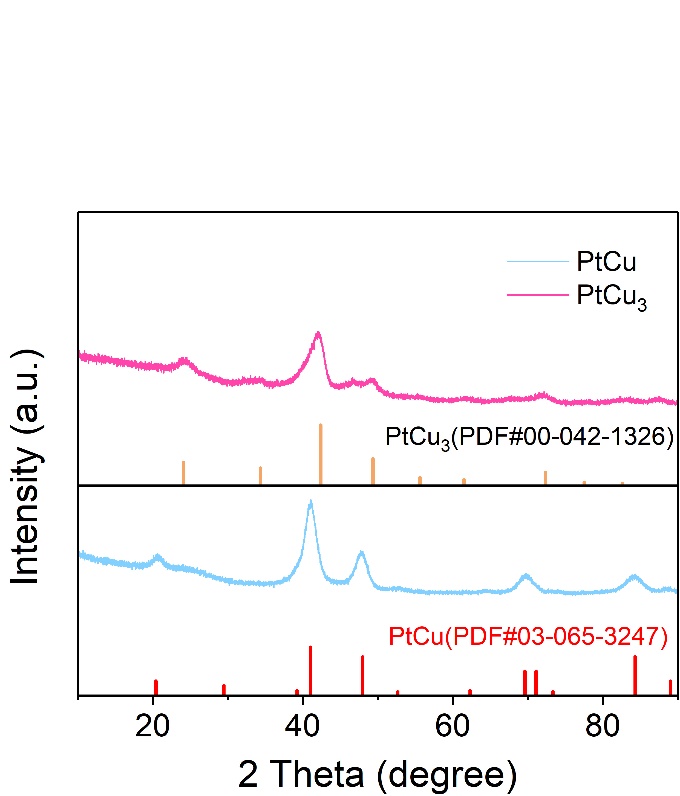


**Supplementary Fig. 24 |.** XRD patterns of PtCu and PtCu_3_.


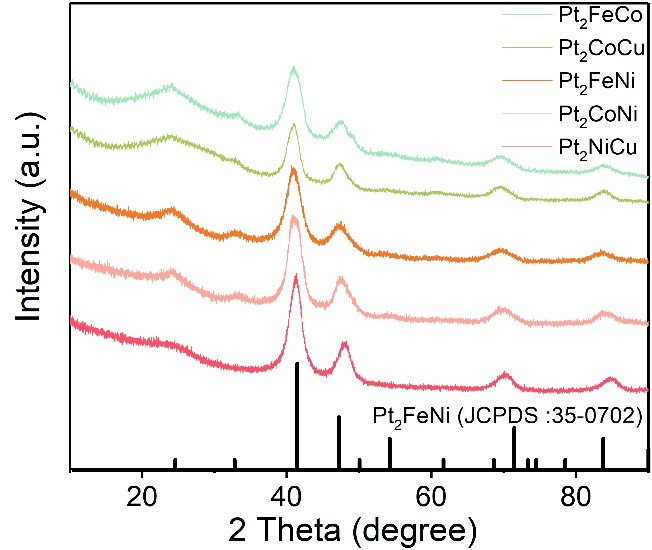


**Supplementary Fig. 25 |** XRD patterns of Pt_2_FeCo, Pt_2_CoCu, Pt_2_FeNi, Pt_2_CoNi, Pt_2_NiCu.Some of the alloys without standard PDF card and others instead.


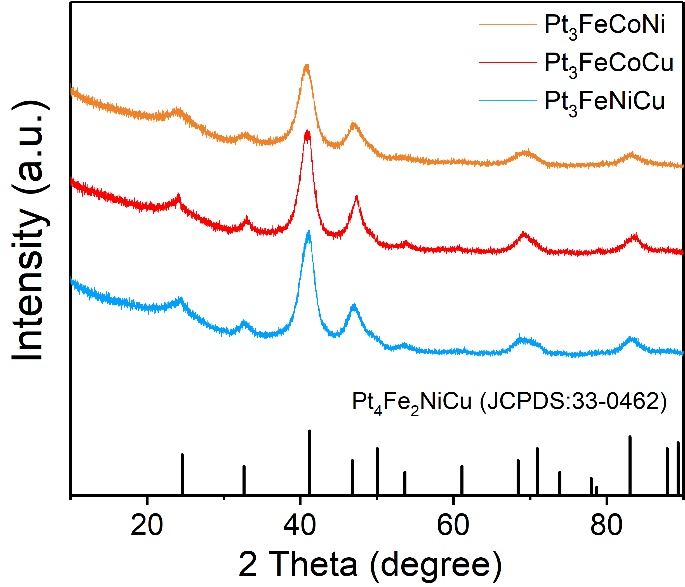
``

**Supplementary Fig. 26 |** XRD patterns of Pt_3_FeCoNi, Pt_3_FeCoCu, Pt_2_FeNiCu, Pt_4_FeCoNiCu. Some of the alloys without standard PDF card and others instead.


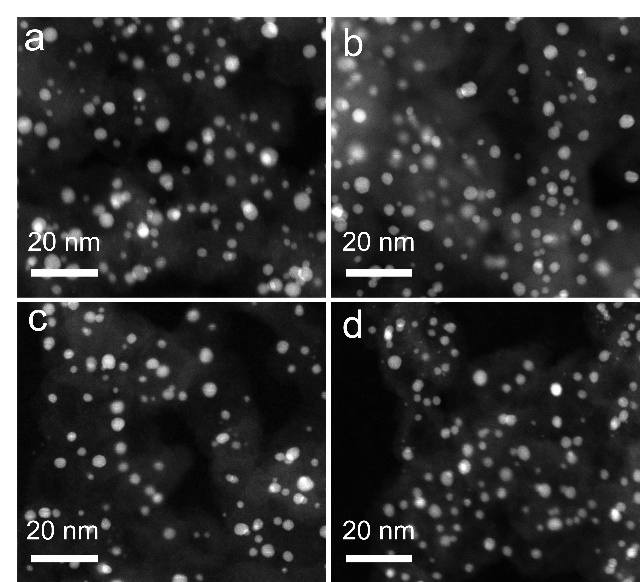


**Supplementary Fig. 27 |** **High-resolution HAADF-STEM images of intermetallic alloy. a,** PtCo/B/C. **b,** Pt_2_FeCu/B/C. **c,** Pt_3_CoNiCu/B/C. **d,** Pt_4_FeCoNiCu/B/C.


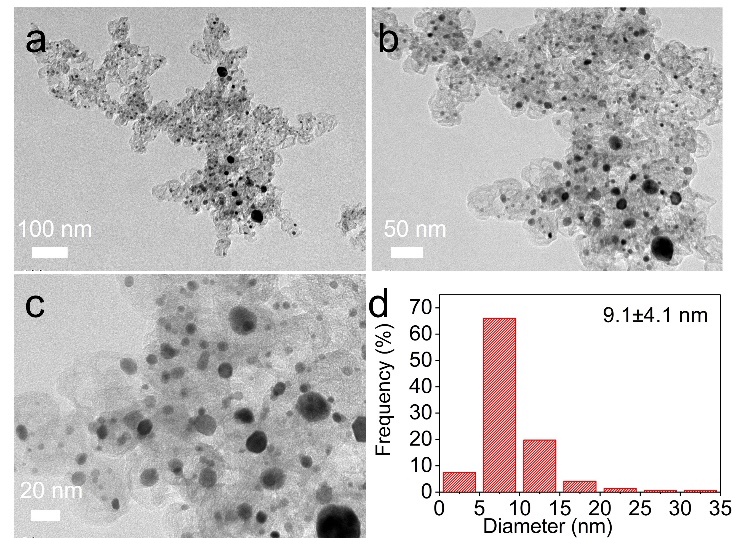


**Supplementary Fig. 28 |** **TEM images and size distributions of PtCo alloy without B. a-c,** TEM images with different enlargement. **d,** The histograms of particle size distribution of corresponding IMCs.The loading amount of alloy is 20 wt.%.


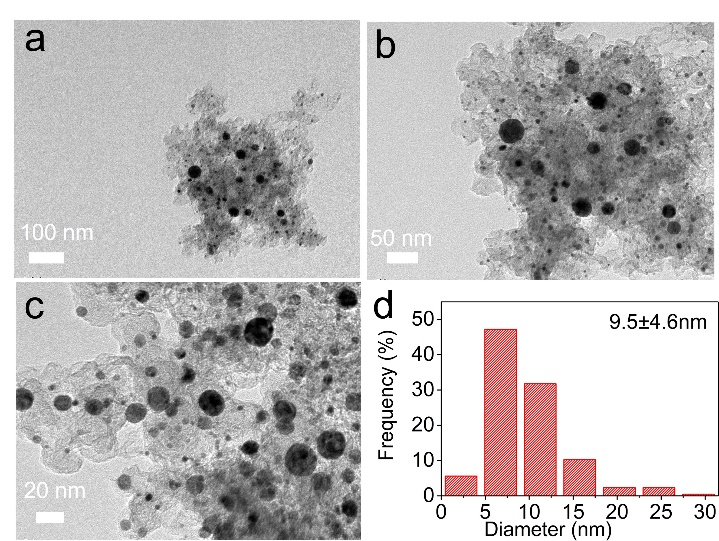


**Supplementary Fig. 29 |** **TEM images and size distributions of Pt_4_FeCoNiCu alloy without B. a-c,** TEM images with different enlargement. **d,** The histograms of particle size distribution of corresponding IMCs.The loading amount of alloy is 20 wt.%.


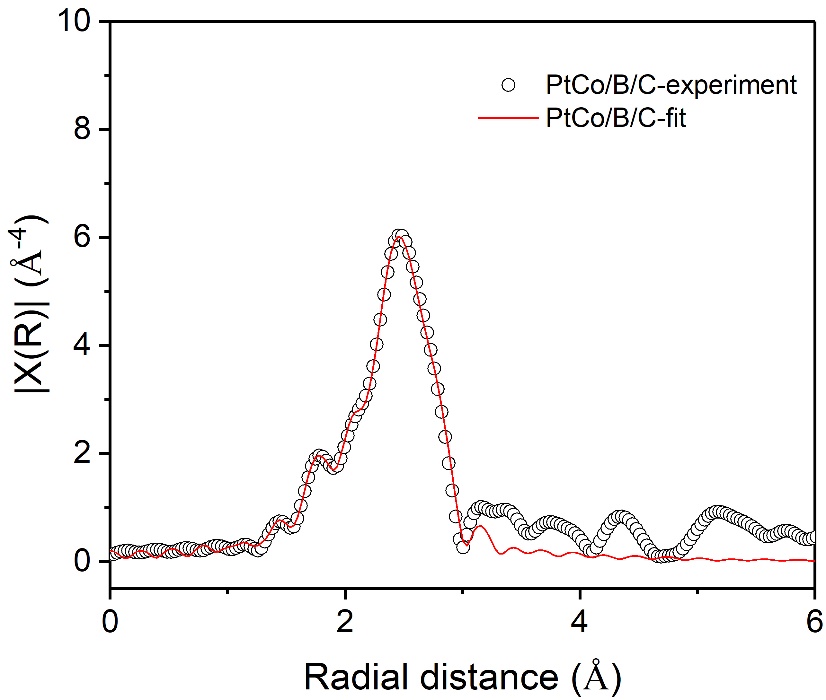


**Supplementary Fig. 30 |** EXAFS fitting spectra at Pt L_3_-edge of PtCo/B/C.


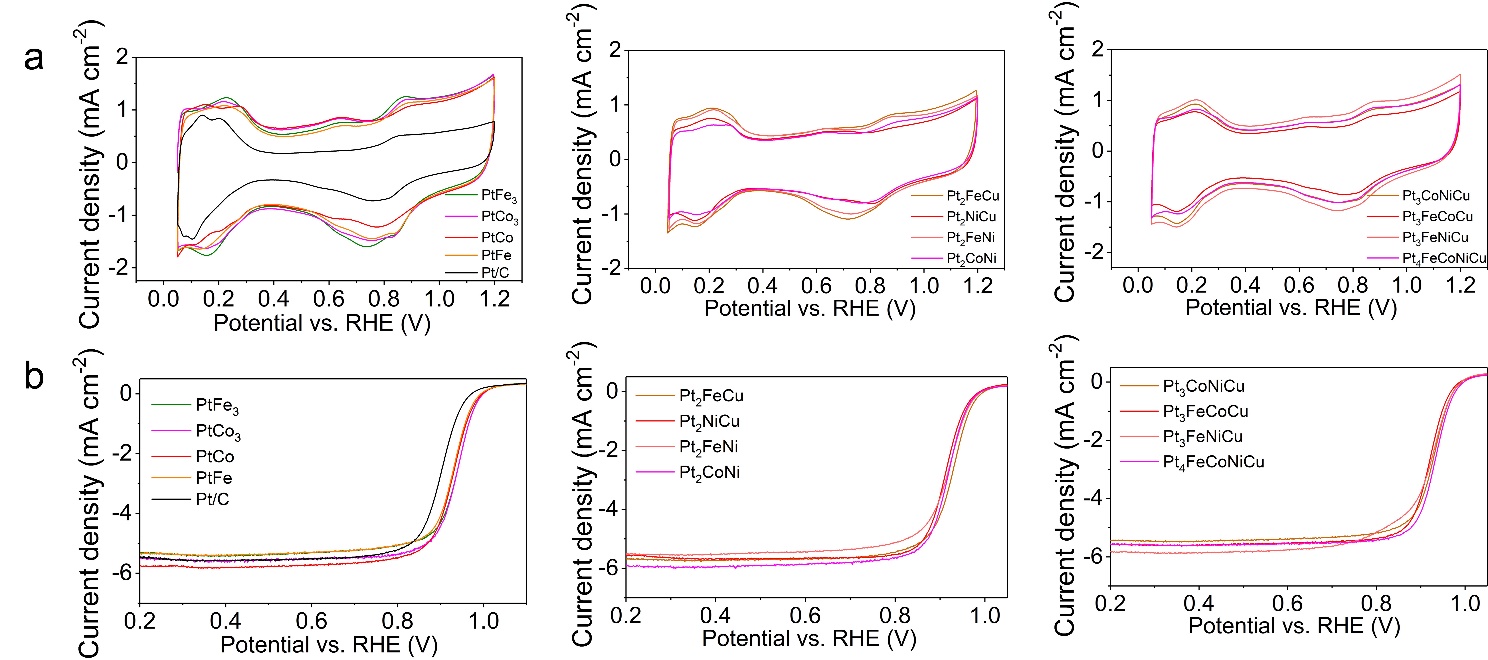


**Supplementary Fig. 31 |** **Electrochemical oxygen reduction reaction. a,** CV curves of Pt-based IMCs/B/C. **b,** ORR polarization curves of Pt-based IMCs/B/C.


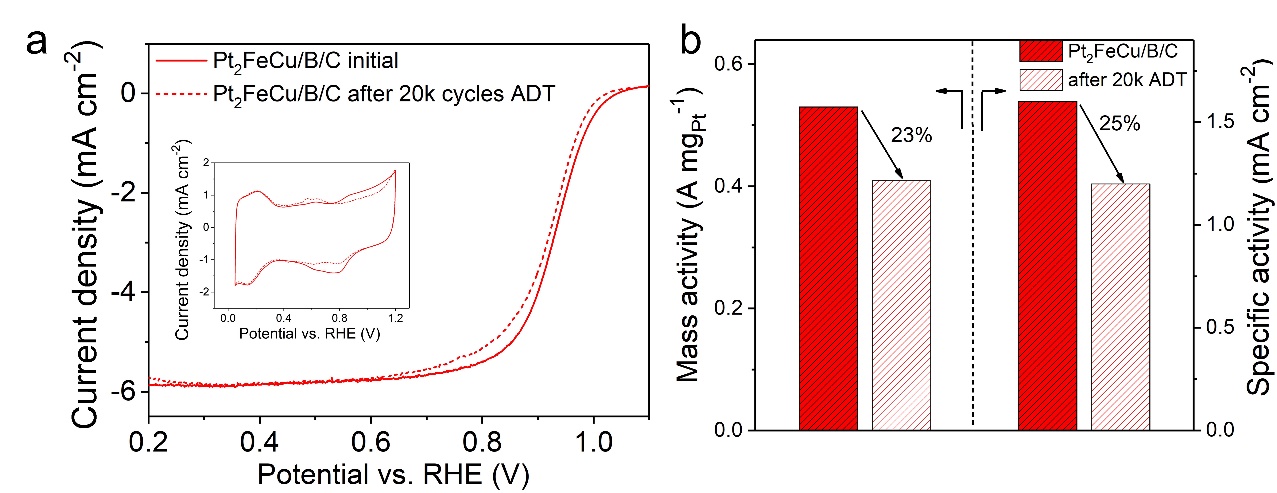


**Supplementary Fig. 32 |** **Electrochemical oxygen reduction reaction.** **a,** CV and LSV curves of Pt_2_FeCu/B/C before and after 20k cycles ADT. **b,** MAs and SAs of Pt_2_FeCu/B/C before and after 20k cycles ADT.


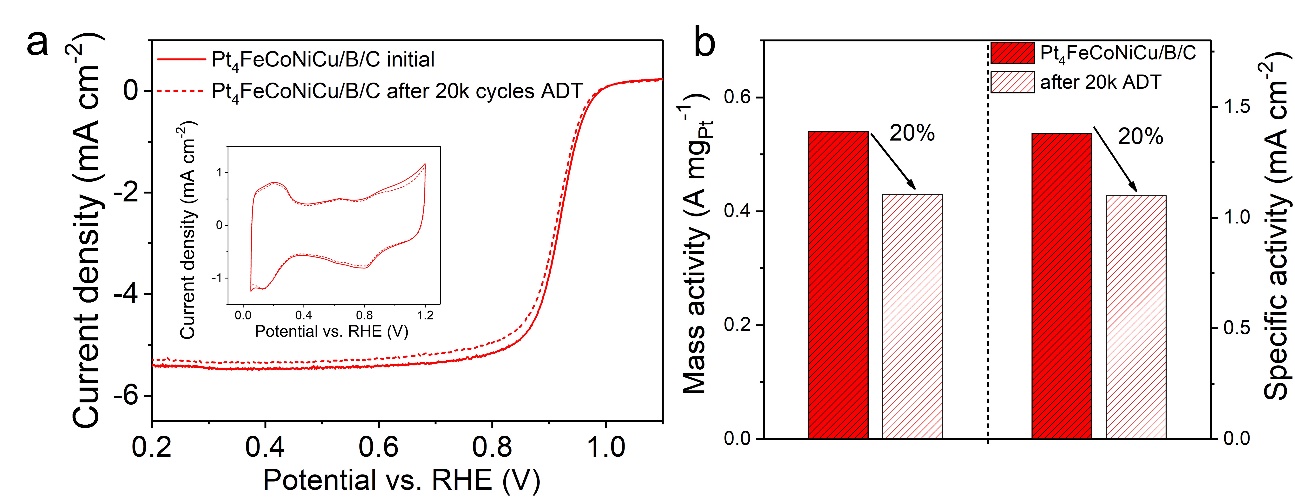


**Supplementary Fig. 33 |** **Electrochemical oxygen reduction reaction.** **a,** CV and LSV curves of Pt_4_FeCoNiCu/B/C before and after 20k cycles ADT. **b,** MAs and SAs of Pt_4_FeCoNiCu/B/C before and after 20k cycles ADT.


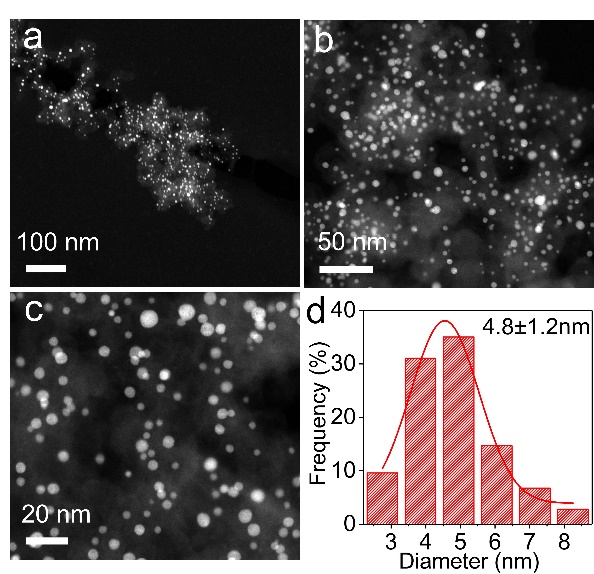


**Supplementary Fig. 34 |** **HAADF-STEM images and size distribution of PtCo/B/C after ADT test. a-c,** HAADF-STEM images with different enlargement. **d,** The histograms of particle size distribution of corresponding IMCs.


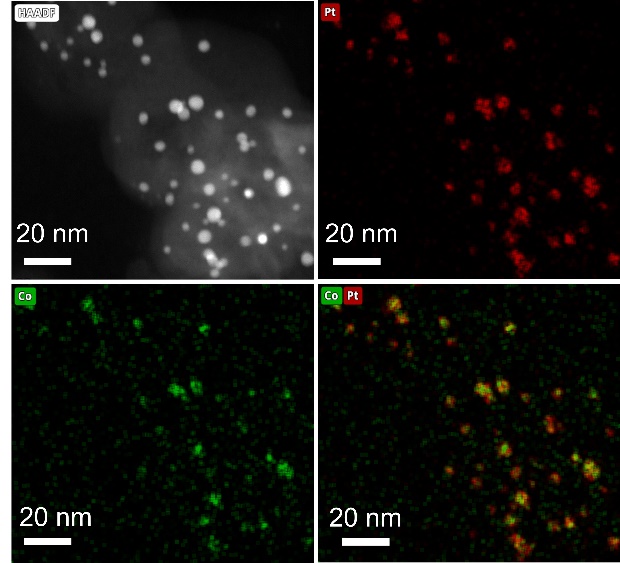


**Supplementary Fig. 35 |** HAADF-STEM images and EDS mapping of PtCo/B/C after ADT test.


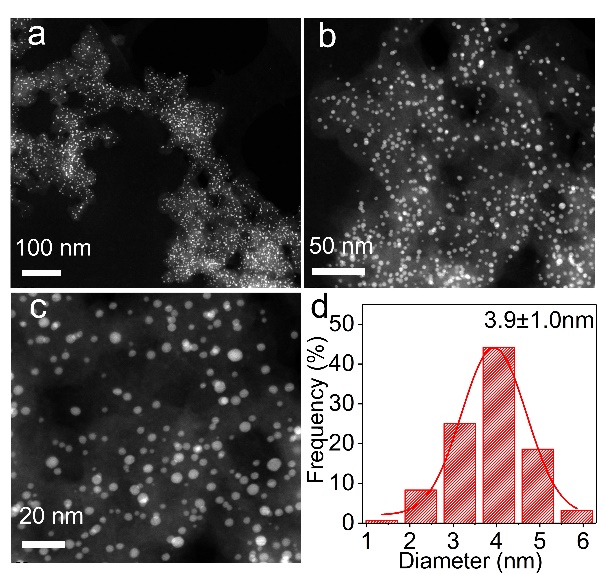


**Supplementary Fig. 36 |** **HAADF-STEM images and size distribution of Pt_2_FeCu/B/C after ADT test. a-c,** HAADF-STEM images with different enlargement. **d,** The histograms of particle size distribution of corresponding IMCs.


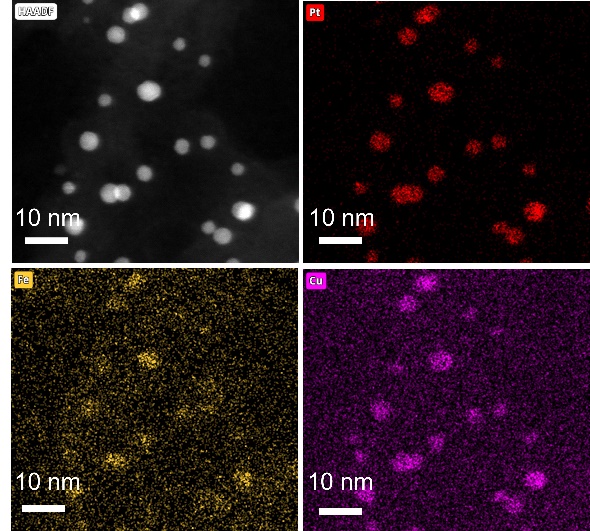


**Supplementary Fig. 37 |** HAADF-STEM images and EDS mapping of Pt_2_FeCu/B/C after ADT test.


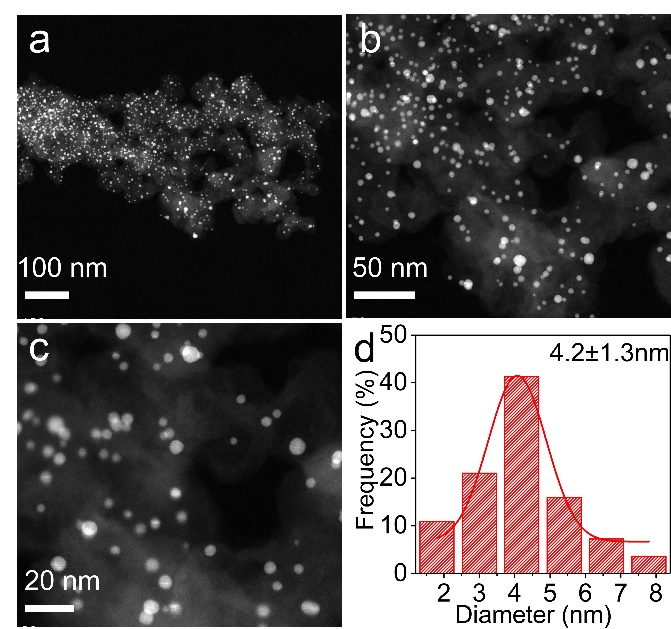


**Supplementary Fig. 38 | Supplementary Fig.38 |** **HAADF-STEM images and size distribution of Pt_3_CoNiCu/B/C after ADT test. a-c,** HAADF-STEM images with different enlargement. **d,** The histograms of particle size distribution of corresponding IMCs.


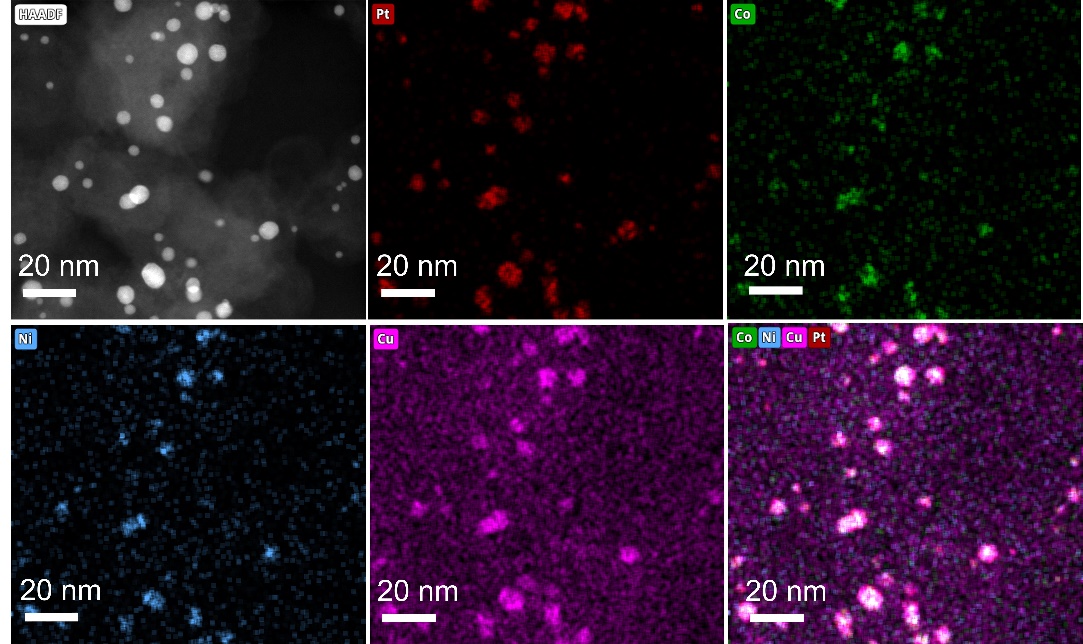


**Supplementary Fig. 39 |** HAADF-STEM images and EDS mapping of Pt_3_CoNiCu/B/C after ADT test.


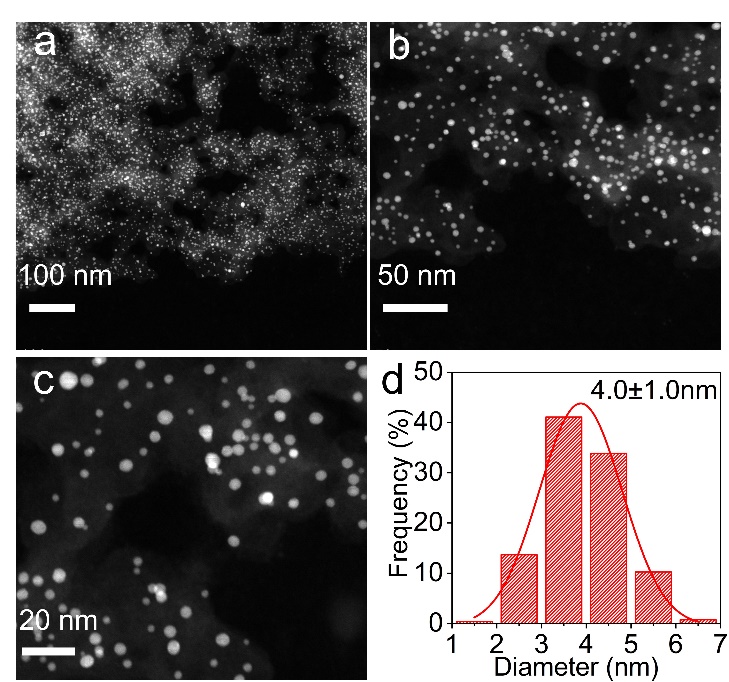


**Supplementary Fig. 40 | HAADF-STEM images and size distribution of Pt_4_FeCoNiCu/B/C after ADT test. a-c,** HAADF-STEM images with different enlargement. **d,** The histograms of particle size distribution of corresponding IMCs.


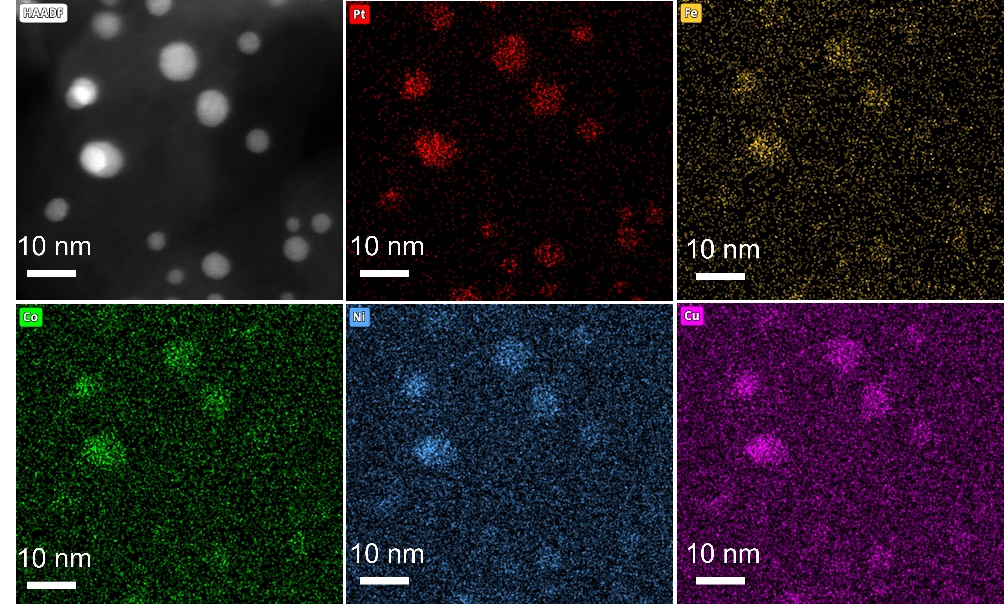


**Supplementary Fig. 41 |** HAADF-STEM images and EDS mapping of Pt_4_FeCoNiCu/B/C after ADT test.


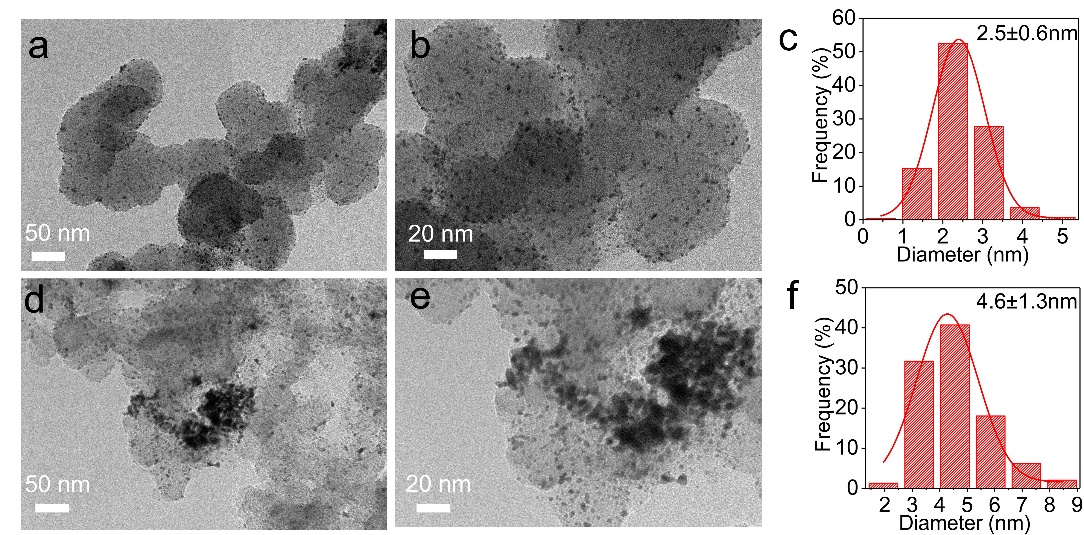


**Supplementary Fig. 42 | HAADF-STEM images and size distribution of commercial Pt/C. a-b,** TEM images with different enlargements of the sample before ADT test. **c,** The histograms of corresponding particle size distribution in **a**. **d-e,** TEM images with different enlargements of the sample after ADT test. **f,** The histograms of corresponding particle size distribution in **d**.

**Supplementary Table 1 |** ECSA of three samples calculated by different methods.

|  | Commercial Pt/C(JM) | B/Pt/C(JM) | Pt/B/C |
| --- | --- | --- | --- |
| ECSA_H_/m^2^g_Pt_^-1^ | 61.5 | 31.1 | 74.5 |
| ECSA_CO_/ m^2^g_Pt_^-1^ | 69.8 | 33.8 | 92.3 |

**Supplementary Table 2 |** Structural parameters extracted from EXAFS fitting at Pt L3-edge of Pt foil, PtO_2_, Pt/B/C, PtCo/B/C in R space.

| Sample | Path | CN | R(Å) | σ^2^*10^-3^(Å) | ΔE_0_(eV) | R-factor |
| --- | --- | --- | --- | --- | --- | --- |
| Pt foil | Pt-Pt | 12.00 | 2.76 | - | - | - |
| PtO_2_ | Pt-O | 6 | 2.02 | - | - | - |
| Pt/B/C | Pt-Pt | 4.71±0.39 | 2.75±0.02 | 6.52±0.62 | 9.05±1.03 | 0.010 |
|  | Pt-B | 1.23±0.11 | 2.22±0.05 | 5.90±0.75 | -11.38±1.75 |  |
| PtCo/B/C | Pt-Pt | 4.82±0.49 | 2.70±0.00 | 8.76±0.50 | 5.95±0.42 | 0.008 |
|  | Pt-Co | 2.57±0.57 | 2.67±0.02 | 9.63±0.79 | 9.64±0.77 |  |
|  | Pt-B | 1.12±0.15 | 2.22±0.03 | 7.38±0.37 | 4.42±0.35 |  |

*CN, coordination number; R, distance between absorber and backscatter atoms; Δσ^2^, change value of Debye-Waller factor relative to the reference compound; ΔE_0_, inner potential correction between the sample and the reference compound. Error bounds (accuracies) of the structural parameters are estimated to be: CN ±20%; R, ±2%; Δσ^2^, ±20%; and ΔE_0_, ±20%.

**Supplementary Table 3 |** Intermetallic alloy loading measuring by ICP-MS.

| IMCs | Total loading  (wt.%) | Pt  (at.%) | Fe  (at.%) | Co  (at.%) | Ni  (at.%) | Cu  (at.%) |
| --- | --- | --- | --- | --- | --- | --- |
| PtCo | 22.9% | 53.1 | - | 46.9 | - | - |
| PtCo_3_ | 22.6% | 34.2 | - | 65.8 | - | - |
| PtFe | 25.3% | 47.9 | 52.1 | - | - | - |
| PtFe_3_ | 30.1% | 26.6 | 73.4 | - | - | - |
| PtCu | 27.2% | 51.8 | - | - | - | 48.2 |
| PtCu_3_ | 32.0% | 35.7 | - | - | - | 64.3 |
| Pt_2_FeCo | 29.0% | 55.5 | 19.8 | 24.7 | - | - |
| Pt_2_FeNi | 26.7% | 57.9 | 20 | - | 22.1 | - |
| Pt_2_FeCu | 27.1% | 64.0 | 6.5 | - | - | 29.5 |
| Pt_2_CoNi | 25.3% | 61.1 | - | 18.8 | 20.1 | - |
| Pt_2_CoCu | 33.9% | 56.2 | - | 23.6 | - | 20.2 |
| Pt_2_NiCu | 30.5% | 59.4 | - | - | 15.4 | 25.2 |
| Pt_3_FeCoNi | 26.2% | 60.1 | 18.5 | 12.1 | 9.3 | - |
| Pt_3_FeCoCu | 31.0% | 52.5 | 10.3 | 21.5 | - | 15.7 |
| Pt_3_FeNiCu | 27.0% | 64.2 | 10.3 | - | 13.6 | 11.9 |
| Pt_3_CoNiCu | 25.1% | 57.3 | - | 16.7 | 15.6 | 10.4 |
| Pt_4_FeCoNiCu | 25.5% | 56.0 | 9.1 | 14.4 | 11.3 | 9.2 |

**Supplementary Methods**

**Reducing capacity of HB.** We qualitatively compared the reducing capacity of HB with some well-known reducing agents at room temperature via the color reaction between K_3_[Fe(CN)_6_] and Fe^2+^. Briefly, H_2_, HB, NaBH_4_, and hydrazine are added (flowed) into a 4 mL solution containing K_3_[Fe(CN)_6_] and Fe(NO_3_)_3_·9H_2_O with a molar ratio of 1:1, respectively. The color changes with time by different reductants are recorded . It is clear that a small amount of Fe^2+^ is produced over time when flowing H_2_ into the solution (a), demonstrating a weaker reducibility of H_2_ toward Fe^3+^ at room temperature. When HB, NaBH_4_, and hydrazine were added to the as-prepared solution, it only takes 3 s for the solution to change color from pale yellow to dark blue, indicating that Fe^3+^ ions are immediately reduced to Fe^2+^ by HB, NaBH_4_, and hydrazine, respectively (b-d). By observing the rate of color changes, we can qualitatively deduce that HB does have considerable reducibility, although weaker than that of other reducing agents (i.e., NaBH_4_, and hydrazine).

**CO stripping Test.** CV curves of these samples were recorded in N_2_-saturated electrolyte at a scan rate of 50 mV s^-1^; CO stripping was carried out by first holding the catalyst electrode at 0.06 V in CO-saturated 0.1 M HClO_4_ for 15 min. Subsequently, N_2_ was purged through the electrolyte for 20 min to remove residual CO and then the electrode was scanned from 0.06 to 1.0 V at 100 mV s^−1^ to record the CO stripping voltammogram. To demonstrate that B was not coated on the surface of Pt nanoparticles in Pt/B/C, a control experiment is conducted. Briefly, 20 wt.% commercial Pt/C(JM), which has similar particle size with our Pt/B/C catalyst (a, b), was mixed with 5 mL methanol suspension of HB by thoroughly stirring for 1 day. And then, 2 mL DI water was added to above mixture, stirring for 1 hour to promote the hydrolysis of HB before drying in vacuum oven. It is obvious that the B layer, which is derived from the hydrolysis of HB, would be coated on the surface of Pt nanoparticles in JM catalyst (denoted as B/Pt/C(JM)). c and d show the incorporation of the B source in the B/Pt/C sample. Moreover, it should be noted that the boron species on the Pt surface are susceptible to oxidation. e and f show the cyclic voltammetry (CV) curves and CO stripping of B/Pt/C(JM), Pt/C(JM), and Pt/B/C, respectively. It is evident that the integrated area of B/Pt/C(JM) sample in both H_2_ underpotential deposition (HUPD) and CO stripping regions drops sharply in contrast to that of the initial Pt/C(JM). Furthermore, the B/Pt/C(JM) sample has the smallest ECSA compared with that of Pt/C(JM), which should be ascribed to the B coating layer on Pt nanoparticles. While our Pt/B/C sample presents the highest specific surface area among these samples (Supplementary Table 1**)**, implying that the Pt nanoparticle in Pt/B/C was not covered by B.
