# Supplementary material for: Hydrogenated borophene enabled synthesis of multielement intermetallic catalysts
Source: Nat Commun. 2023 Nov 16;14:7414. doi: 10.1038/s41467-023-43294-z (PMC10654666; doi:10.1038/s41467-023-43294-z)
Supplement: Supplementary file 2 — Peer Review [file 41467_2023_43294_MOESM2_ESM.pdf]

nature portfolio

Peer Review File

Hydrogenated borophene enabled synthesis of multielement intermetallic catalysts

Open Access This file is licensed under a Creative Commons Attribution 4.0 International License, which permits use, sharing, adaptation, distribution and reproduction in any medium or format, as long as you give appropriate credit to the original author(s) and the source, provide a link to the Creative Commons license, and indicate if changes were made. In the cases where the authors are anonymous, such as is the case for the reports of anonymous peer reviewers, author attribution should be to 'Anonymous Referee' followed by a clear attribution to the source work. The images or other third party material in this file are included in the article's Creative Commons license, unless indicated otherwise in a credit line to the material. If material is not included in the article's Creative Commons license and your intended use is not permitted by statutory regulation or exceeds the permitted use, you will need to obtain permission directly from the copyright holder. To view a copy of this license, visit http://creativecommons.org/licenses/by/4.0/.

REVIEWER COMMENTS

Reviewer #1 (Remarks to the Author):

In this work, the authors demonstrate an unusual reduction technique which allows them to prepare very small Pt and Pt-alloy nanoparticle catalysts which exhibit significant sintering resistance. In particular, the catalysts show hugely improved stability at high temperatures. This capability is key towards forming Pt intermetallic catalysts, and very few strategies have been demonstrated for performing this. These intermetallic catalysts are of very high importance for a number of electrocatalytic transformations, but their stability during synthesis is a major hurdle. For this reason I support the publication of this manuscript subject to major revisions.
I have three main criticisms of the manuscript:

1. There is very little understanding of what happens to the boron inside the catalyst material. Furthermore, the mechanism by which the boron enhances sintering stability requires further refinement. This is presumably quite insoluble stuff, and will coat the surface of the catalyst, which prevents a lot of the sintering. Anchoring effects have been comprehensively reported by the group of Andy (Xueliang) Sun and others, where merely depositing trace quantities of inert Mo, La, or Nb oxides by ALD massively reduces the mobility of Pt nanoparticles across the surface of carbon supports. It is rather difficult to differentiate these purely mechanical/physical effects from chemical effects which strengthen the attachment of the Pt particle to the support. Unfortunately the boron is challenging to probe with electron microscopy or even X-ray techniques.

If, as proposed by the authors, boron-rich supports provide all their stability enhancement through purely chemical effects which enhance the particle-support interactions, these should be obvious in control experiments. The stability of Pt nanoparticles on boron-doped carbon supports or boride composites should be similarly enhanced. However this is not what we see in the literature, where N and B doped systems have either relatively small or no effect on the attachment strength of Pt nanoparticle catalysts. If the morphology of the borophene precursor is important, this would also indicate physical vs chemical effects in the stabilization mechanism.

The authors do engage with this topic using DFT modelling, which supports the enhanced adsorption strength onto boron films. However the calculations are not very compelling and the conditions seem arbitrarily selected. The choice of the model is going to influence the result quite strongly, and I see no evidence for the model proposed in Fig 1a or Fig S9.

I recommend the authors broaden their discussion to include alternative hypotheses for the stability enhancement. Otherwise, much more evidence is required to support the strong claims being made about Pt-B bonds stabilizing the particles. The results in this manuscript demonstrating enhanced stability are very significant, especially with the thoughtfully selected in-situ TEM work. Even if we do not have a precise explanation for their stability, the paper stands on the high quality of the imaging alone.

2. The quality of the English writing impedes the clarity of the work, and numerous grammatical errors and awkward translations are contained throughout the manuscript. This paper would benefit from further review by a native speaker, there are several parts where the meaning and interpretation of the science is unfortunately clouded by the writing quality. For example, the XRD analysis discusses superlattice “facets”, which should instead refer to

“planes”.

3. Not enough experimental detail is given to reproduce the work. Several examples of this are given in the miscellaneous comments below

Line 54 the intrinsic activity of catalysts for many of these reactions actually increases as particle size grows. Taking ORR as an example, the well known “particle size effect” shows that ultrasmall particles (~<2nm) have activities perhaps 10x smaller than extended polycrystalline surfaces. This has driven crystallite sizes upwards for many of these reactions, optimizing the mass activity for catalysts at a point significantly larger than the smallest practically obtainable size, balancing surface area and specific activity.
In the introduction, it is claimed that HB has significant reducability. Please indicate how strong of a reducing agent HB is, perhaps by quoting the standard reduction potential. If there are strong kinetic barriers as one might expect for a solid state reductant, specify the relative strength vs other well known reducing agents (eg H2, hydrazine, or BH4)
In Figure 1B, what is the loading? This sample with a particle size of 2.0nm does not seem to correspond to any of the samples seen in the 1f plot. The others also have similar problems, for example part e shows 2.1nm at 40wt, but 1f shows over 2.5nm at 40wt%.
The counts in the histograms should not be reported in arbitrary units
10min is rather short to usefully say the materials are super stable at high temperatures. Can we crystallize intermetallics formed at temperatures for such brief periods of time?
In the in situ TEM experiment, which is great, we see obvious sintering in the lower right corner. It is difficult to say onset temperature, since the images are not of particularly high resolution in the figure. More importantly, I have no idea over which length of time these data were acquired. The comparison with the JM material is both appropriate and striking.
The boron XPS data appear to be severely overfitted, and not useful in my interpretation of the results. Is there a reason why the signal is so poor for the Pt@B sample? More detail must be given towards exactly which sample is being measured in the XPS. More experimental details about the XPS must also be given. Please also show the survey spectra in the SI.
The XANES/NEXAFS is interesting, and does show differences between the Pt foil and the Pt@B sample. However, the real comparsion we are interested in is the Pt@C sample. The wavelet analysis certainly shows a peak which I agree could be a Pt-C or Pt-B length. However the text interprets this as the formation of a bond, which in my opinion is not supported or especially useful here. When you adsorb atoms (from ALD, for example) onto any catalyst support, you will see the associated internuclear distances in the EXAFS. However these are simply distances, and not necessarily bonds. This is why the Pt supported on a traditional carbon support is a much better comparison, since we don’t usually speak of Pt@HSAC as exhibiting organometallic Pt-C bonds. In the Fig 3 analysis of the PtCo@B/C, the Pt-C correlation length is noticeably absent from the labelling.
The temperature and treatment conditions for the alloy samples needs to much more precisely detailed. It is difficult, for example, to understand what sample Fig S19 represents. How it was annealed? What is the support?
The images in Fig 3 c-f are much too low resolution to understand the particle size distributions shown as inset.

Reviewer #2 (Remarks to the Author):

The authors propose utilizing highly reductive hydrogen borophene (HB) to produce an

electrocatalyst characterized by small size, high-density dispersion, and robust stability, which is primarily attributed to the formation of the Pt-B bond. Moreover, they have successfully demonstrated the synthesis of a wide array of ultrafine binary, ternary, quaternary, and quinary intermetallic catalysts using the Pt@B module. These catalysts exhibit remarkable stability, resisting particle sintering even when annealed at 1,000°C. The issue of maintaining particle size during heat treatment is of paramount importance when it comes to producing thermodynamically stable alloys, given that an increase in catalyst loading may induce severe sintering via particle coalescence. As the authors present a novel strategy characterized by a dependable morphology, this reviewer recommends the manuscript for publication in Nature Communications, once the remaining issues have been addressed.
(1) The authors have loaded the Pt@B module onto the support. How can it be confirmed whether Pt nanoparticle adheres to the support or B is loaded onto the support? Isn't there a possibility that B covering the surface of Pt could prevent sintering?
(2) There will be lattice mismatching between B and the support. And this mismatching will likely affect the electronic and geometric structure of Pt and B. It might be beneficial to discuss the implications of this. Such explanation is missing in the DFT model.
(3) In the XPS data, the binding energy of Pt 4f has increased, which typically signifies a decrease in oxygen adsorption strength due to a d-band downshift. However, the authors explain this trend in reverse. The transfer of electrons from Pt to B implies a decrease in activity due to an upshift of the d band. An explanation is needed for this.
(4) The changes in Pt's electronic structure induced by B are likely to result in changes in activity and durability. I recommend adding DFT results for this and discussing it alongside physicochemical analyses, including XPS and EXAFS.
(5) The double layer of the catalyst with the PtB module appears very wide. Materials with a wide double layer often exhibit low electrochemical reactivity. It is necessary to discuss whether the presence of B could potentially act as a resistance to activity (excluding changes in the catalyst's electronic structure) and whether it possesses adequate electrical conductivity.
(6) Writing out the full name of IMCs when it first appears would be helpful for the readers to understand.
(7) The Pt/C ORR curve appears to be underestimated compared to previous reports. Verification is necessary. Using an Ag/AgCl electrode as a reference electrode may not be appropriate. Cl contamination can interfere with the ORR reaction of Pt, leading to potential underestimation. Additionally, it is recommended to specify whether the data has been corrected for IR (Ohmic) drop in the experimental section.

Reviewer #3 (Remarks to the Author):

Zeng et al. proposed to use borophene-stabilized strategy to synthesize Pt@B module catalyst, which can be further dispersed on various carbon and metal oxide-based supports. To show the application of this material, they studied electrocatalytic ORR as a demonstration. The emphasis of this work is the stability of materials. Here are questions regarding the stability of materials and the local structure of Pt@B.

1. Based on Fig. 1 and Fig. S2, in Pt@B, the average size of Pt is about 2 nm. But after depositing Pt@B to other supports, the Pt size changes depending on the type of supports and the weight loading of Pt@B. Why would that happen if Pt has a strong interaction with B? Does that mean that after the addition of substrates, Pt could migrate to substrates?

2. Do you have evidence showing that Pt remains interacting with B after the addition of substrates?

3. This work seems emphasizing the concept of Pt@B module. Was this type of idea proposed before?

4. TEM is a local technique and based on the shapes of the particles, I feel it is hard to estimate the particle size. What is the size of Pt based on XAS data?

5. Lines 156-159, "As shown in Supplementary Figs.4 , the TEM images of Pt@B/C acquired from 25℃ to 1000 ℃ and corresponding size analysis show the well-maintained dispersion and limited growth of nanoparticles." What are the results from the corresponding size analysis? The size distributions of Pt after different temperatures are not shown in Fig. S4.

6. I think for catalysts, their stability under reactive conditions is the big challenge. I would suggest the authors testing the stability of Pt@B under temperature dependent reducing conditions.

7. According to XPS (fig. 2d), Pt2+ and Pt0 coexist. What is Pt2+ species? Can you see this species from XAS data?

8. Lines 222-223, "…..scattering peak of Pt@B at ~2.45 Å, which is smaller than Pt foil, can be assigned to the contribution of Pt-Pt/Pt-B bonds." I don't think it can be assigned to Pt-B bonds. Cannot be that long.

9.Lines 223-224, "Due to heteroatomic interactions in Pt@B, a shortened Pt-Pt distance is displayed compared to the standard Pt foil." Do not understand this sentence.

10. Please show y scales in Fig 2g,h. Based on Fig. 2g, the intensity of oscillation of Pt@B is weaker than that of Pt foil but the peak intensity of Pt@B in R space appears larger.

11. Inconsistency of R space spectra and WT-EXAFS maps. In WT-EXAFS, somehow Pt-B/O contribution shifts to higher R. As said, Pt2+ was observed from XPS. Can the low R contribution due to Pt-O?

12. Can the authors distinguish Pt-O and Pt-B bond? What are their bond distances? What is the Pt-Pt coordination number? What is the size of Pt based on EXAFS analysis?

13. Lines 270-271, "…..which can be assigned to the mix of Pt-Co, Pt-B, Pt-O coordination (Fig. 3i), and the…." Where is this O coming from?

Manuscript ID: NCOMMS-23-12450-A

Title: Response to Reviewer Comments on the Paper " ***Borophene-stabilized multielement catalysts*** "

Response Letter to Reviewers

(*Black italic: Editor's remarks*; Blue type: Our response; Red type: Our revision)

From reviewer's comments, we noticed that the naming of Pt@B might cause misunderstanding to readers about the structure of the as-prepared samples, because A@B usually means a core-shell structure. Therefore, we have replaced the @ to / (e.g. Pt@B/C to Pt/B/C, PtM/B/C in the revised manuscript.

To better reflect the focus of the article, we have revised the title to " ***Hydrogenated borophene enabled synthesis of ultrafine multielement catalysts***".

Point-by-Point Response to Reviewers' Comments

Reviewer #1:

In this work, the authors demonstrate an unusual reduction technique which allows them to prepare very small Pt and Pt-alloy nanoparticle catalysts which exhibit significant sintering resistance. In particular, the catalysts show hugely improved stability at high temperatures. This capability is key towards forming Pt intermetallic catalysts, and very few strategies have been demonstrated for performing this. These intermetallic catalysts are of very high importance for a number of electrocatalytic transformations, but their stability during synthesis is a major hurdle. For this reason I support the publication of this manuscript subject to major revisions.

Response: We appreciate the reviewer for the positive and valuable comments on our paper, which help us make a significant improvement in the quality of our article. We hope that these revisions address all the concerns and make the manuscript complete. We truly thank you for your time and attention.

Comment 1. *There is very little understanding of what happens to the boron inside the catalyst material. Furthermore, the mechanism by which the boron enhances sintering stability requires further refinement. This is presumably quite insoluble stuff, and will coat the surface of the catalyst, which prevents a lot of the sintering. Anchoring effects have been comprehensively reported by the group of Andy (Xueliang) Sun and others, where merely depositing trace quantities of inert Mo, La, or Nb oxides by ALD massively reduces the mobility of Pt nanoparticles across the surface of carbon supports. It is rather difficult to differentiate these purely mechanical/physical effects from chemical effects which strengthen the attachment of the Pt particle to the support. Unfortunately the boron is challenging to probe with electron microscopy or even X-ray techniques.*

If, as proposed by the authors, boron-rich supports provide all their stability enhancement through purely chemical effects which enhance the particle-support interactions, these should be obvious in control experiments. The stability of Pt nanoparticles on boron-doped carbon supports or boride composites should be similarly enhanced. However this is not what we see in the literature, where N and B doped systems have either relatively small or no effect on the attachment strength of Pt nanoparticle catalysts. If the morphology of the borophene precursor is important, this would also indicate physical vs chemical effects in the stabilization mechanism.

The authors do engage with this topic using DFT modelling, which supports the enhanced adsorption strength onto boron films. However the calculations are not very compelling and the conditions seem arbitrarily selected. The choice of the model is going to influence the result quite strongly, and I see no evidence for the model proposed in Fig 1a or Fig S9.

I recommend the authors broaden their discussion to include alternative hypotheses for the stability enhancement. Otherwise, much more evidence is required to support the strong claims being made about Pt-B bonds stabilizing the particles. The results in this manuscript demonstrating enhanced stability are very significant, especially with the thoughtfully selected in-situ TEM work. Even if we do not have a precise explanation for their stability, the paper stands on the high quality of the imaging alone.

Response: We are grateful for the reviewer's comment about the refinement and further discussion of the sintering resistance mechanism in our system Pt/B/C (i.e., physical *vs.* chemical effects). We agree with the reviewer that both physical and chemical effects between Pt-B can induce largely enhanced stability yet with different origins and mechanisms. As the reviewer mentioned, boron is challenging to probe with electron microscopy or even X-ray techniques; In the revision stage, we mainly derived the structural model and stabilization mechanism of our catalysts from (1) a detailed explanation of our sample preparation process, (2) a key control sample of B/Pt/C by forming B sheets physically covering on Pt nanoparticles in Pt/C, (3) DFT calculation for strong Pt-B chemical interaction, and (4) XAS probing of the Pt-B bond/interaction. As a quick summary, we attribute the enhanced stability of our Pt/B/C catalysts to mostly the chemical effects rather than physical covering.

Sample preparation process. First, for the preparation of Pt/B/C catalysts, HB was firstly mixed with Ketjen carbon black to form HB/C composite support structure; Pt precursor was then added to the HB/C composite support and reduced by HB to form Pt/B/C catalysts (**Figure R1**). Since Pt is lastly added to HB/C and was in situ reduced by HB to form Pt nanoclusters, it is unlikely that Pt nanoclusters were physically covered by the HB or Boron sheets to induce any severe physical covering effect.

Below is the detailed sample preparation process. HB and carbon black were mixed in methanol *via* thorough stirring for 5 h to form the HB/C compound suspension by taking advantage of the strong adsorption of HB with a 2D morphology to carbon black. The precursor salt of Pt dissolved in methanol is then added to the above suspension and stirred for three days. During such a process, the Pt ions were reduced to Pt (0) by HB. The following growth of Pt nanoparticles was on the basis of the in-situ formed Pt clusters that have been already anchored on the surface of C through B layer. Therefore, from the view of the preparation process, the Pt nanoparticles loaded on C is unlikely to be coated by the B-layer.

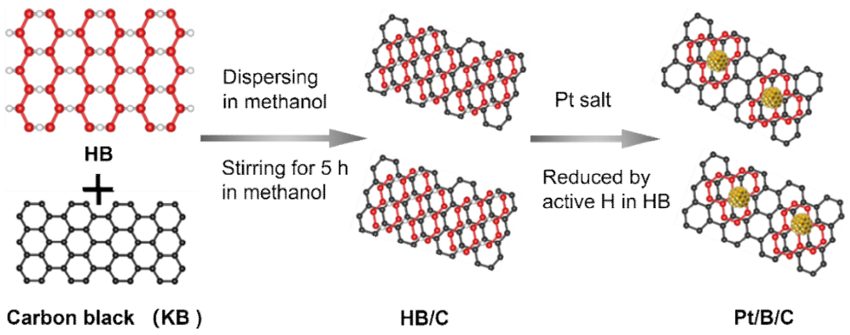

Figure R1-1. Schematic diagram of the synthesis process of Pt/B/C.

Control sample B/Pt/C. Second, a key control sample (B/Pt/C) was also synthesized and compared with Pt/B/C to indirectly demonstrate that B was not coated on the surface of Pt nanoparticles in our Pt/B/C. To illustrate the B physical covering effect, we purposely synthesized B/Pt/C by covering the Pt/C with HB or Boron layers. Briefly, 20 wt.% commercial Pt/C(JM), which has a similar particle size to our Pt/B/C catalyst (**Figure R1-2a and 2b**), was mixed with 5 mL methanol suspension of HB by thoroughly stirring for 1 day. And then, 2 mL DI water was added to the above mixture, stirring for 1 hour to promote the hydrolysis of HB and Boron layer coating. It is obvious that the B layer, which is derived from the hydrolysis of HB, would be coated on the surface of Pt nanoparticles in JM catalyst (denoted as B/Pt/C(JM)).

Figures R1-2c and 2d show the cyclic voltammetry (CV) curves and CO stripping of B/Pt/C(JM) - B physical covering, Pt/C(JM) – no B, and Pt/B/C – our catalysts, respectively. It is evident that the integrated area of B/Pt/C(JM) sample in both H_2 underpotential deposition (HUPD) and CO stripping regions drops sharply in contrast to that of the initial Pt/C(JM), while our Pt/B/C sample presents the highest specific surface area among these samples (**Table R1-1**). Considering Pt nanoparticles are similar (size and loading) in these three samples, such a drastic change in surface exposure implies different surface structures: the smallest ECSA in B/Pt/C should be ascribed to the B physical coating layer on Pt nanoparticles, which further proves that the Pt nanoparticle in our Pt/B/C was mostly exposed and not covered by B.

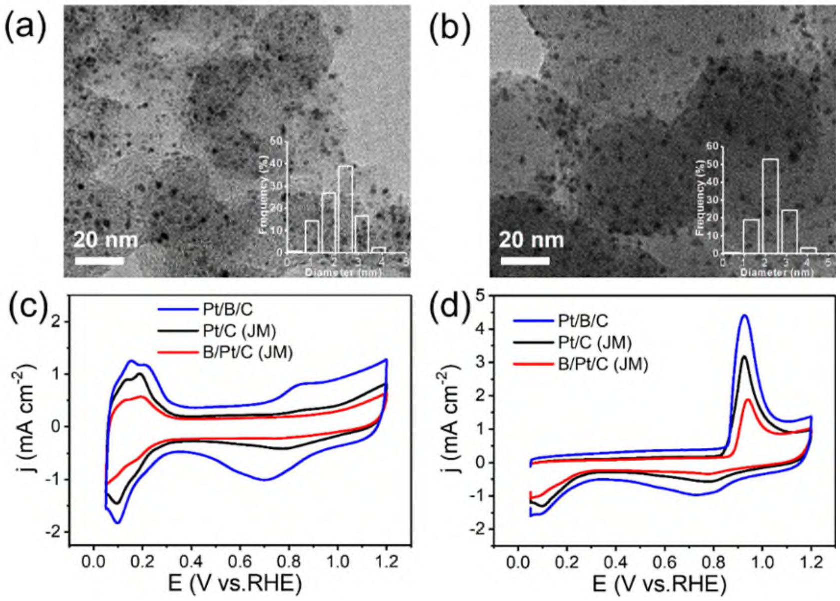

Figure R1-2. (a- b), TEM of Pt/B/C and commercial Pt/C (JM) catalyst; (c-d), CV curves of and CO-stripping of Pt/B/C (as-prepared), commercial Pt/C (JM) and B/Pt/C (JM). Test conditions: CV curves of these samples were recorded in N_2-saturated electrolyte at a scan rate of 50 mV s^{-1}; CO stripping was carried out by first holding the catalyst electrode at 0.06 V in CO-saturated 0.1 M $HClO_4$ for 15 min. Subsequently, N_2 was purged through the electrolyte for 20 min to remove residual CO and then the electrode was scanned from 0.06 to 1.0 V at 100 mV s^{-1} to record the CO stripping voltammogram. Production process, Pt/C: purchased; Pt/B/C: as-prepared; B/Pt/C: Pt/C was mixed with 5 mL methanol suspension of HB by thoroughly stirring for 1 day. And then, 2 mL DI water was added to above mixture, stirring for 1 hour to promote the hydrolysis of HB before drying in vacuum oven.

Table R1-1. ECSA of three samples calculated by different methods.

	Pt/C	B/Pt/C	Pt/B/C
$ECSA_H/m^2g_{Pt}^{-1}$	61.5	31.1	74.5
$ECSA_{CO}/ m^2g_{Pt}^{-1}$	69.8	33.8	92.3

DFT calculation of strong Pt-B interaction. The above discussion can eliminate

significant physical effects in our Pt/B/C catalysts. To illustrate the chemical effects, in our original manuscript, we used DFT to calculate the chemical interaction between Pt and B. Our DFT calculation results in Supplementary Figure 14 indicated that the adsorption energy of Pt cluster on B/C was much stronger than that directly on graphene (KB), thus theoretically proving the strong chemical interaction in Pt/B/C to support enhanced stability.

Since it is hard to build a model with real atomic numbers, we chose some stable structures with low atomic numbers to qualitatively study the general trend of adsorption stability. The calculation results indicate that the interaction of Pt-B is stronger than Pt-C. In addition, we also calculated the anti-sintering ability of Pt/B/C by simulating the Ostwald ripening processes which involve the migration of individual atoms driven by the surface energy of nanoparticles (*Yin et.al., Nat. Commun., 2021,12:3135*). We calculated the escape energy of the atom in Pt_{13} which is furthest from the substrate (graphene or borophene). It is obvious that the escape energy of individual atoms from Pt_{13} cluster on borophene (4.68 eV) was much larger than that on graphene (3.16 eV), implying a stronger interaction exists when Pt nanoparticles grown on B/C than that directly on carbon (**Figure R1-3**).

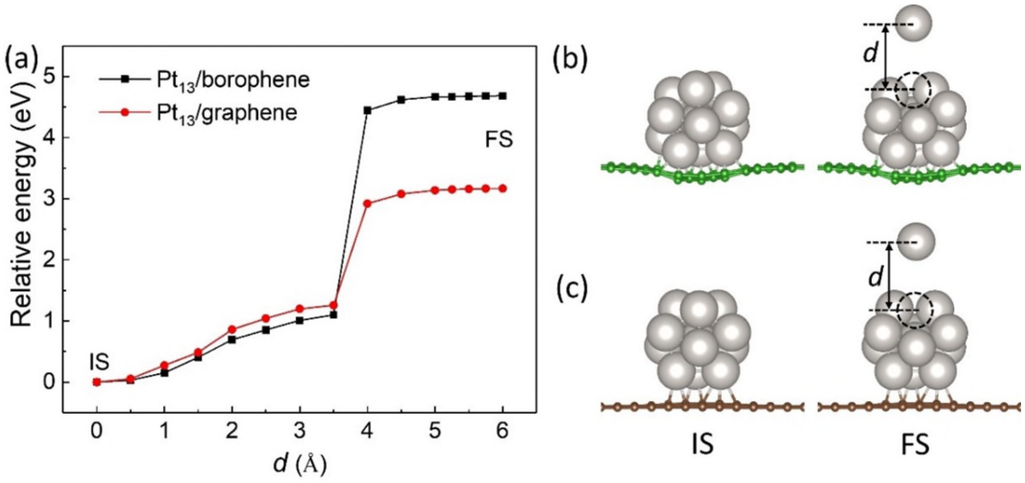

Figure R1-3. Theoretical investigations of Pt atom escaping from Pt_{13} cluster loaded on graphene and borophene. (a) Escape energy of one selected atom (top site) from the Pt_{13} cluster on graphene and borophene; (b) and (c), the diagram of the escaping Pt atom from Pt_{13} cluster loaded on graphene and borophene, respectively.

XAS probing of Pt-B interaction. Lastly, although B is hard to probe, we performed the XAS analysis of Pt/B/C to demonstrate the chemical interaction of Pt-B. Before analysis, by consulting the Crystallography Open Database (COD) developed by the University of Cambridgeit (www.crystallography.net), the slight difference in lengths of Pt-O (2.02 Å), Pt-B (2.19 Å) bond was found. So, we raised the Fourier transform weight in FT-EXAFS spectrum from k^2 in original manuscript to k^3 latest revised manuscript, which can increase the resolution to distinguish Pt-O and Pt-B. From the EXAFS spectrum of Pt/B/C, an apparent peak in 1.78 Å indicating the Pt-B bond and no sign of Pt-O occurs compared to Pt/C and PtO_2 (**Figure R1-4**). In addition, the structural parameters extracted from EXAFS fitting at Pt L_3-edge in R space of Pt/B/C also indicate the existence of Pt-B bond in the Pt/B/C sample and has a bond length of 2.22 Å (**Figure R1-5** and **Table R1-2**), different from Pt-O (2.02 Å). Based on these analyses, we believe that there is a chemical bond between Pt and B.

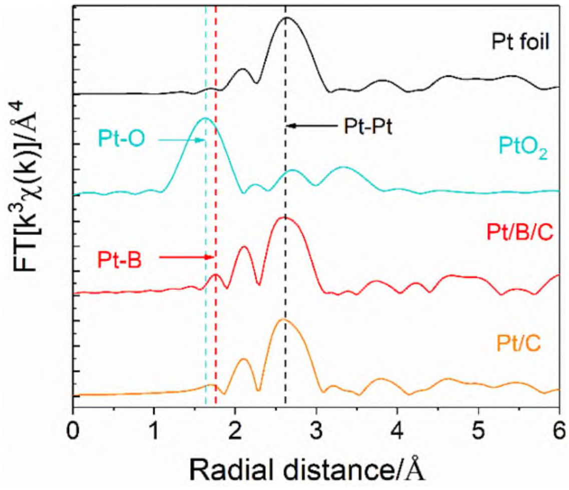

Figure R1-4. EXAFS spectra at Pt L_3-edge for Pt foil, PtO_2, Pt/B/C, commercial Pt/C in R space.

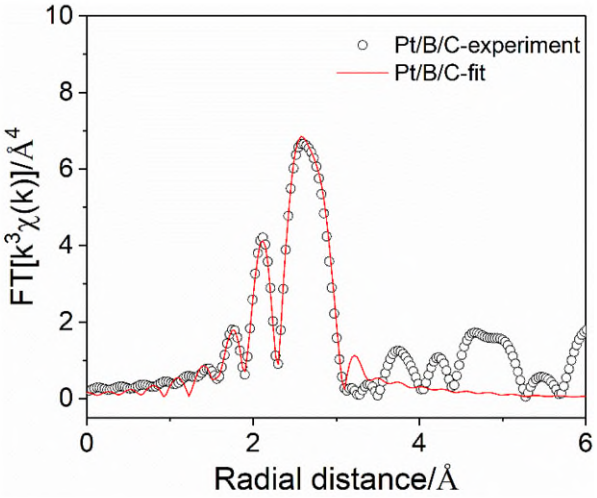

Figure R1-5. Fitting results of EXAFS at Pt L_3-edge of Pt/B/C.

Table R1-2. Structural parameters extracted from EXAFS fitting at Pt L_3-edge of Pt foil, PtO_2, Pt/B/C in R space.

Sample	Path	CN	R(Å)	$\sigma^2 * 10^{-3}$(Å)	ΔE_0(eV)	R-factor
Pt foil	Pt-Pt	12.00	2.76	-	-	-
PtO_2	Pt-O	6	2.02	-	-	-
Pt/B/C	Pt-Pt	4.71±0.39	2.75±0.02	6.52±0.62	9.05±1.03	0.010
	Pt-B	1.23±0.11	2.22±0.05	5.90±0.75	-11.38±1.75	

*CN, coordination number; R, distance between absorber and backscatter atoms; σ^2, change value of Debye-Waller factor relative to the reference compound; ΔE_0, inner potential correction between the sample and the reference compound. Error bounds (accuracies) of the structural parameters are estimated to be: CN ±20%; R, ±2%; $\Delta\sigma^2$, ±20%; and ΔE_0, ±20%.

In summary, from the above explanation, control samples and experiments, theoretical calculation, and detailed characterization, we believe that the chemical interaction between Pt-B is the main reason to enhance the particle-support interactions,

thus improving the sintering stability of Pt/B/C.

Modification in the revised manuscript:

(1) line 118

In the experiment, we conducted a two-step synthesis procedure to fabricate the catalyst, as illustrated in Figure 1a. Firstly, the as-prepared HB was mixed with activated carbon to form HB/C support. Subsequently, a solution containing H_2PtCl_6 was introduced, where the Pt^{4+} ions was then reduced by the bridging hydrogen atoms in HB, along with the split of hexagonal boron network, due to the breakup of the terminal B-H bond. The following growth of Pt nanoparticles was on the basis of the *in-situ* formed Pt clusters that has been already anchored on the surface of C through B layer.

(2) line 133

Our additional experiments have revealed that, in contrast to the encapsulation mechanism often reported in the literature[31], this stabilization effect is achieved by employing B fragments as a stabilizing medium between activated carbon and Pt nanoparticles (See Supplementary Fig. 4 and Table 1).

(3) line 190

Additionally, the anti-sintering ability of Pt/B was also demonstrated by simulating the Ostwald ripening processes which involves the migration of individual atom driven by surface energy of nanoparticles. We calculated the escape energy of the atom in Pt_{13} which is furthest from the substrate (graphene or borophene). Notably, the escape energy of individual atom from Pt_{13} cluster on borophene (4.68 eV) was obviously larger than that on graphene (3.16 eV) (Supplementary Fig. 15). These calculation results confirmed that the strong interaction between metal and B sheet is the key reason for improving the thermal stability of Pt nanoparticles[34]. Furthermore, the bonding energy between graphene and borophene layers the equilibrium position was reported higher than that between graphene layer (-0.035 eV/atom vs. -0.02 eV/atom), indicating a stronger adsorption of carbon toward borophene fragments, which is also conductive to the stabilization of Pt nanoaprticles on support[35].

(4) line 234

Fourier transform of EXAFS data in *R* space is shown in Fig. 2f. Compared to Pt-O bond (located at 1.63 Å) in PtO_2, the apparent peak located at 1.78 Å can be assigned to Pt-B scattering. Subsequent fitting results further confirmed the existence of Pt-B bond in Pt/B/C with a bond length of 2.22 Å (Supplementary Fig. 17 and Table 2), consistent with the computation model after relaxation in DFT calculation (Supplementary Fig. 14). Furthermore, the corresponding wavelet transformed (WT)-EXAFS analysis was performed, which is a precise approach to distinguish the backscattering atoms even though they overlap substantially in R space. As shown in Fig. 2g, the Pt/B/C sample exhibits two maximums on y axis, one is located at *ca.* 2.8 Å which is ascribed to the Pt-Pt coordination, consistent with Pt foil reference, and the other ($R+\alpha$: *ca.* 2.2 Å) which is different from Pt-O ($R+\alpha$: *ca.* 2 Å) in PtO_2 reference can be assigned to the Pt-B bond. The combination of these analyses conclusively demonstrates that the Pt is loaded on borophene, enhancing the adhesive energy between nanoparticles and the B/C support while suppressing aggregation and sintering of Pt nanoparticles at elevated temperatures.

(5) Fig. 2 has been updated

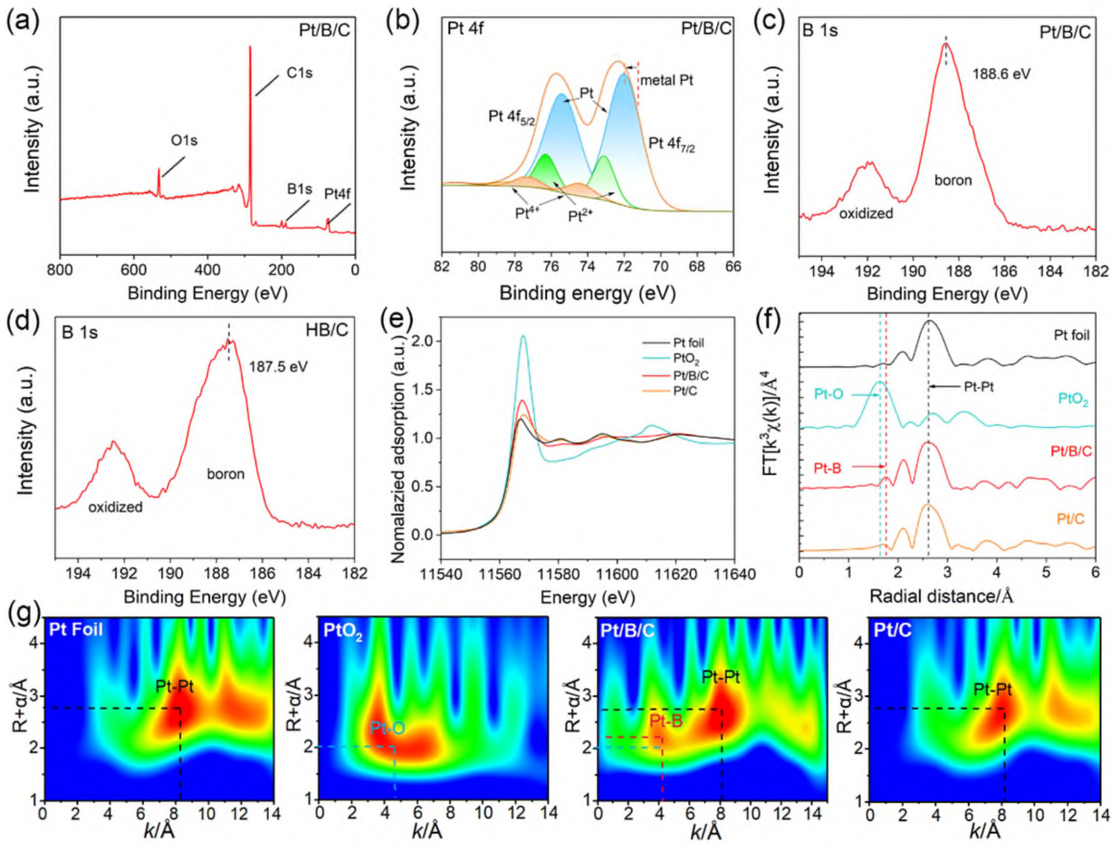

Fig.2 | Interaction of Pt-B analyzed in the experiment . (a) survey spetra XPS of Pt/B/C. (b) Pt 4f XPS of Pt/B/C. (c) B 1s XPS of Pt/B/C. (d) B 1s XPS of HB/C. (e) Pt L_3-edge XANES spectra of Pt/B/C, along with PtO_2, Pt foil and commercial Pt/C. (f) Pt L_3-edge EXAFS spectra of Pt/B/C, with PtO_2, Pt foil and commercial Pt/C. (g) WT-EXAFS of the Pt L_3-edge signal of the Pt foil, PtO_2, Pt/B/C and commercial Pt/C.

Modification in the Supplementary Information:

(1) **Figure R1-2** was listed in revised **Supplementary Information** as **Supplementary Figure 4.**

(2) **Figure R1-3** was listed in revised **Supplementary Information** as **Supplementary Figure 15.**

(3) **Figure R1-5** was listed in revised **Supplementary Information** as **Supplementary Figure 17.**

(4) **Table R1-1** was listed in revised **Supplementary Information** as **Supplementary Table 1.**

(5) **Table R1-2** was listed in revised **Supplementary Information** as **Supplementary Table 2.**

Comment 2. *The quality of the English writing impedes the clarity of the work, and numerous grammatical errors and awkward translations are contained throughout the manuscript. This paper would benefit from further review by a native speaker, there are several parts where the meaning and interpretation of the science is unfortunately clouded by the writing quality. For example, the XRD analysis discusses superlattice "facets", which should instead refer to "planes".*

Response: Thanks for the suggestion from the reviewer. We have checked the manuscript carefully and revised the errors accordingly in the revised manuscript.

Comment 3. *Not enough experimental detail is given to reproduce the work. Several examples of this are given in the miscellaneous comments below.*

Response: We appreciate the expert's attention to the details and we tried our best to show the experiment detail point-by-point as mentioned by the reviewer.

1. Line 54 the intrinsic activity of catalysts for many of these reactions actually increases as particle size grows. Taking ORR as an example, the well known "particle size effect" shows that ultrasmall particles (~<2nm) have activities perhaps 10x smaller than extended polycrystalline surfaces. This has driven crystallite sizes upwards for many of these reactions, optimizing the mass activity for catalysts at a point significantly larger than the smallest practically obtainable size, balancing surface area and specific activity.

Response: We are grateful for the reviewer's valuable comments and we agree with the reviewer. Therefore, balancing mass activity and specific activity should be a very promising research topic. In the current case, decreasing the particle size of PtM

intermetallic nanoparticles could help to increase the mass activity. What we emphasized is that the harsh environments during prolonged operation (such as high temperatures, redox atmosphere, and strongly acidic or alkaline chemical environments) challenge the stability of catalysts, thus causing either material failure (*i.e.*, metal dissolution) or weak metal-support interaction (*i.e.*, catalysts sintering) that greatly affect the performance, rather than the relationship between size effect and catalytic activity.

2. In the introduction, it is claimed that HB has significant reducability. Please indicate how strong of a reducing agent HB is, perhaps by quoting the standard reduction potential. If there are strong kinetic barriers as one might expect for a solid state reductant, specify the relative strength vs other well known reducing agents (eg H_2, hydrazine, or BH_4)

Response: We truly appreciate the reviewer's valuable comment about the reducibility of HB. Frankly, HB possesses significant reducibility to reduce Pt ions to form Pt nanoparticles relative to most inert supports (carbon, oxide, *etc.*). However, its reducibility may not be outstanding or fabulous among various reducing agents. We have revised the improper description which may have confused the reviewer. Furthermore, we also qualitatively compared the reducing capacity of HB with some well-known reducing agents as mentioned by the reviewer at room temperature *via* the color reaction between $K_3[Fe(CN)_6]$ and Fe^{2+}.

Briefly, H_2, HB, $NaBH_4$, and hydrazine are added (flowed) into a 4 mL solution containing $K_3[Fe(CN)_6]$ and $Fe(NO_3)_3 \cdot 9H_2O$ with a molar ratio of 1:1, respectively. The color changes with time by different reductants are recorded, as shown in **Figure R1-6**. A small amount of Fe^{2+} is produced over time when flowing H_2 into the solution **(Figure R1-6a**), demonstrating weaker reducibility of H_2 toward Fe^{3+} at room temperature. When HB, $NaBH_4$, and hydrazine were added to the as-prepared solution, it only takes 3 s for the solution to change color from pale yellow to dark blue, indicating that Fe^{3+} ions are immediately reduced to Fe^{2+} by HB, $NaBH_4$, and hydrazine,

respectively (**Figure R1-6b-d**). By observing the rate of color changes, we can qualitatively deduce that HB does have considerable reducibility, although weaker than that of other reducing agents (i.e., $NaBH_4$, and hydrazine).

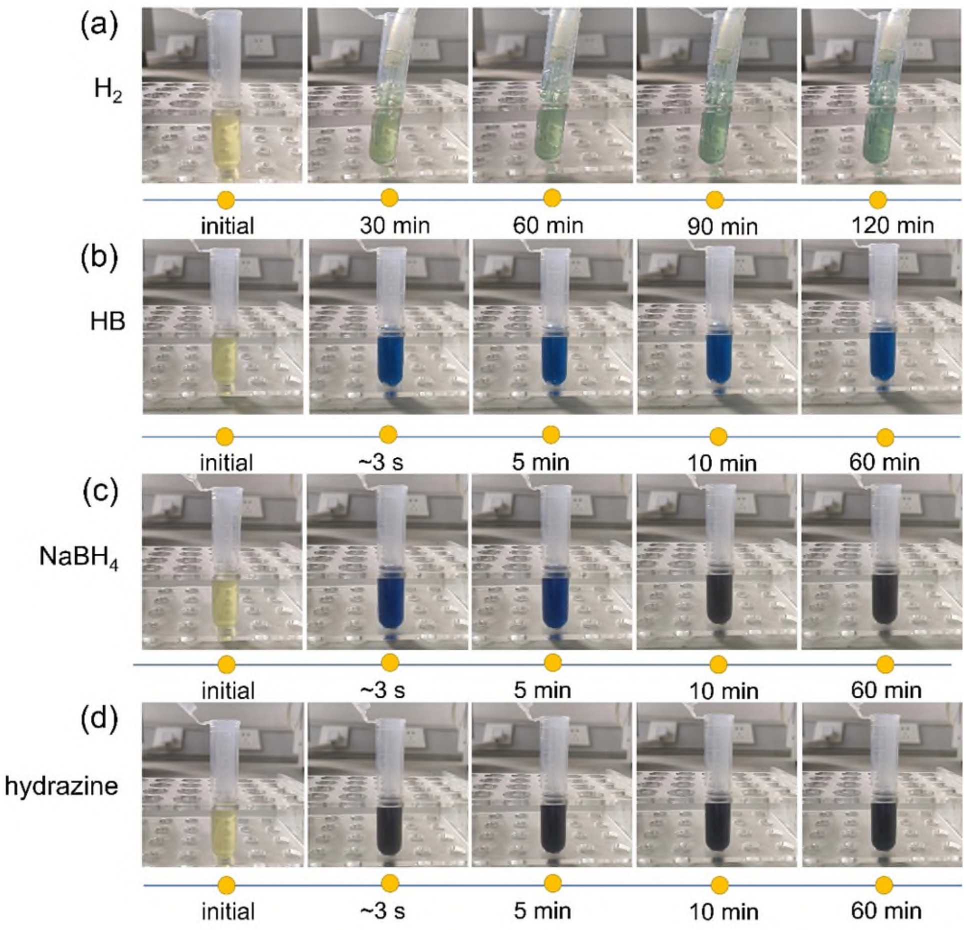

Figure R1-6. Color changes of the solution containing $K_3[Fe(CN)_6]$ and Fe^{3+} in a molar ratio of 1:1 over time when added (a) H_2, (b) HB, (c) $NaBH_4$, (d) hydrazine, respectively.

Modification in the revised manuscript:

(1) line 85

It is worth noting that the active hydrogen present in HB exhibits a notable reducibility, making it a valuable reducing agent for the synthesis of catalysts and related materials.

(2) line 115

The hydrogen in HB exhibited a noble reducibility, as confirmed through a series of control experiments (see **supplementary Fig. 2**).

Modification in revised Supporting Information:

(1) **Figure R1-5** was listed in revised **Supplementary Information** as **Supplementary Figure 2.**

3. In Figure 1B, what is the loading? This sample with a particle size of 2.0 nm does not seem to correspond to any of the samples seen in the 1f plot. The others also have similar problems, for example part e shows 2.1 nm at 40wt, but 1f shows over 2.5 nm at 40wt.%.

Response: We are grateful for the reviewer's comment about some details of Figure 1. In the original manuscript, Figures 1b-e show the Pt NPs loaded on different supports, i.e., the Pt/B itself, Pt/B loaded on Ketjen carbon black, and Pt/B loaded on graphene, respectively. Figure 1f shows the size distribution of Pt with increasing the loading amount for the Pt on carbon black (C). Although we have labeled the necessary information in the upper left corner of each TEM in Figure 1, it inevitably still caused the reader to misinterpret the relationship between Figures 1b-f, and associate Figures 1b-e with Figures f. Thus, we reorganized Figure 1 and presented the HAADF images of Pt/B/C with loading amounts from 20 wt. % to 80 wt.%, which is consistent with **Figure 1f** (as shown in **Figure. R1-6**). In addition, the TEM images of Pt/B, Pt/B/CNT, Pt/B/TiO_2, Pt/B/Al_2O_3, Pt/B/CeO_2 and Pt/B/graphene were presented in supplementary information.

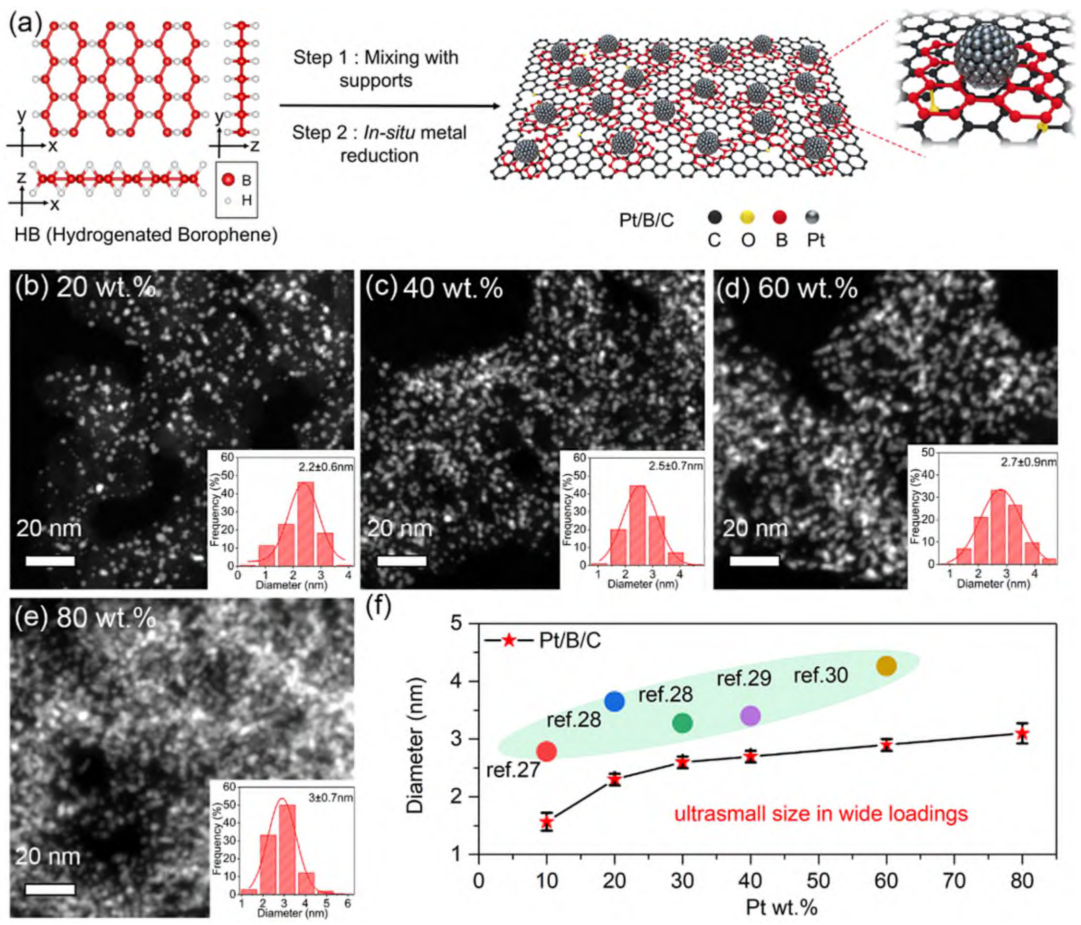

Figure R1-7. Synthesis of Pt/B/C catalyst. (a) Schematic illustration of the process of borophene-stabilized metal catalysts. (b-e) HAADF-STEM images of Pt/B synthesized on KB with Pt loading amounts of 20, 40, 60 and 80 wt.%, respectively. (f) Size distribution of Pt with increasing loading amount for the Pt/B on KB as well as those from the previous literature.

Modification in the revised manuscript:

(1) line 126

However, in our case, even with an increased loading percentage of metallic Pt on carbon (specifically Ketjen black, ECP-600JD, KB) from 10% to as high as 80%, the Pt nanoparticles maintained a uniform and ultrasmall size (Figures 1b-f and Supplementary Fig. 3).

(2) **Figure R1-6** was listed in revised manuscript as **Fig. 1.**

(3) line 137

Moreover, our synthesis strategy exhibits high versatility by allowing the facile loading of metal nanoparticles onto various supports, such as carbon nanotubes, graphene, Al_2O_3, TiO_2 and CeO_2 et.al. (See **Supplementary Fig. 5**)

Modification in revised Supporting Information:

(1)

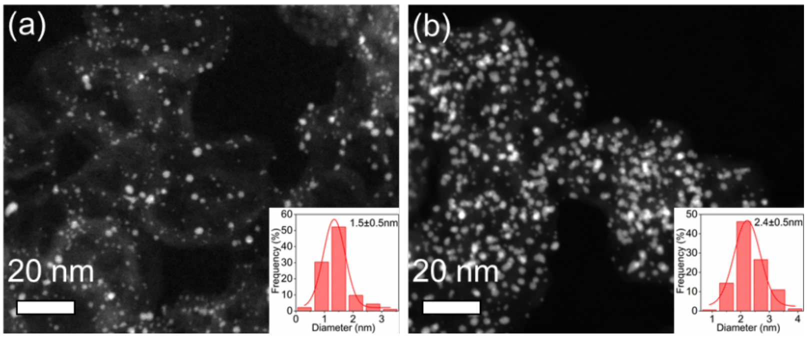

Supplementary Figure 3. HAADF-STEM images and size distribution of Pt/B/C with Pt loading amounts of 10, and 30wt.%, respectively.

(2)

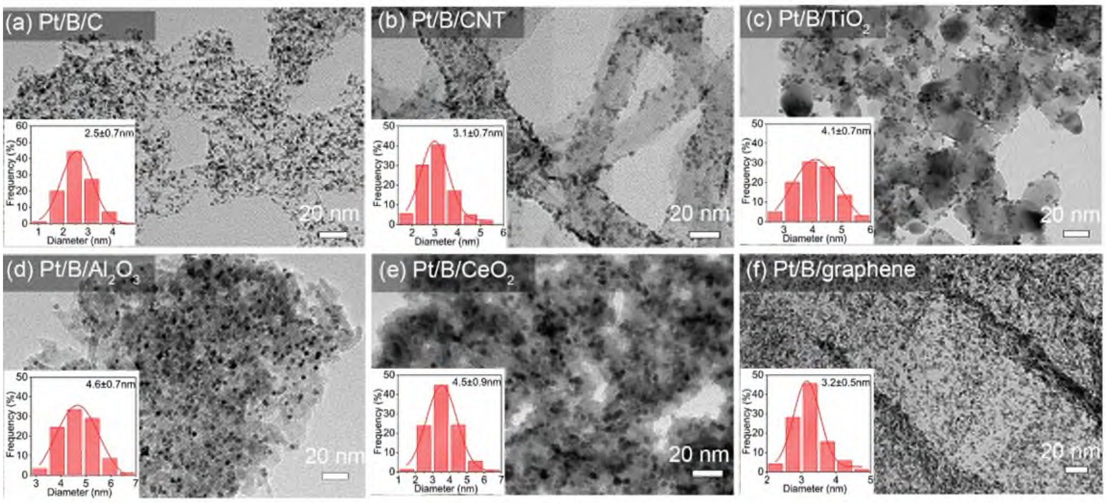

Supplementary Figure 5. TEM image and size distribution of (a) Pt/B/C, (b) Pt/B/CNT, (c) Pt/B/TiO_2, (d) Pt/B/Al_2O_3, (e) Pt/B/CeO_2, (f) Pt/B/graphene. The loading amount of Pt is 40 wt.%, 40 wt.%, 30 wt.%,30 wt.%, 30 wt.%, 80 wt.% for (a), (b), (c), (d), (e) and (f), respectively.

4. The counts in the histograms should not be reported in arbitrary units

Response: We have modified the units of all histograms.

5. 10 min is rather short to usefully say the materials are super stable at high temperatures. Can we crystallize intermetallics formed at temperatures for such brief periods of time?

Response: We are grateful for the reviewer's valuable comments. It is hard to observe the crystallization of intermetallic nanoparticles at 600 ℃ in such a short time. While the trend of particle sintering can be observed visually using the in-situ TEM experiment. What we show here is to monitor the sintering behavior of Pt NPs loaded on B/C substrate. As mentioned in the manuscript, the distance between two labeled nanoparticles keeps consistent during heating for 10 min, indicating a relativity high stability of Pt NPs on the support of B/C. Moreover, a series of ex-situ heating experiments, in which the sample of Pt/B/C was heated for 2h at the temperature of 200, 400, 600, 800, and 1000 ℃, were performed to further prove the stabilization of Pt/B/C. The TEM images obtained from the sample after different heating treatments were shown in **Figure R1-7**. It can be seen that the Pt NPs show little growth, further proving that the as-prepared sample has excellent anti-sintering performance. However, as a contrast, a serious sintering occurred in 60 wt.% Pt/C (JM) at 600 ℃ for 2h heating treatment.

We are grateful for the reviewer's valuable comments. The data of 10 min *in-situ* TEM at 600 ℃ is just to monitor the sintering behavior of Pt NPs loaded on B/C substrate. As for the stability of the material, there are a series of *in situ* and *ex-situ* experienment.(**Figure R1-8** and **Supplementary Figure 6**). In addition, all IMCs were obtained at high temperature held one hour.

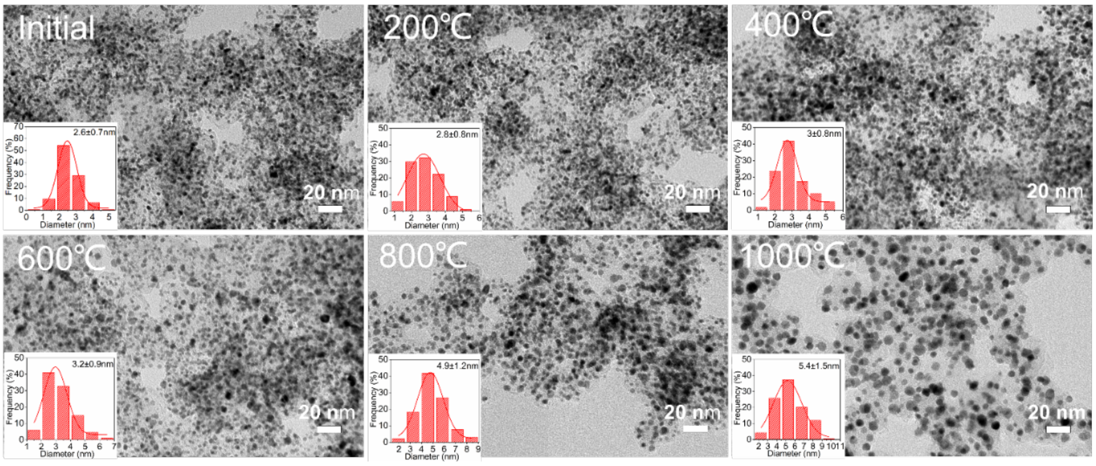

Figure R1-8. Ex-situ TEM images of Pt/B/C after calcinated at room temperature, 200℃, 400℃, 600℃, 800℃ and 1000 ℃ for 2 h with a heating rate of 5 ℃/min, respectively.

Modification in the revised manuscript:

(1) line 170

To avoid the influence of transmission electron beam on the morphology of the catalyst during the in-situ observation and evaluate the stability of Pt/B/C under reducing conditions, the catalyst was subjected to annealing at different temperatures for 2 hours in a tube furnace under a 5% H_2/Ar atmosphere. The resulting morphology was characterized by TEM, and the corresponding images are presented in Supplementary Fig. 10.

Modification in revised Supporting Information:

(1) **Figure R1-7** was listed in revised **Supplementary Information** as **Supplementary Figure 10**

6. In the in-situ TEM experiment, which is great, we see obvious sintering in the lower right corner. It is difficult to say onset temperature, since the images are not of particularly high resolution in the figure. More importantly, I have no idea over which length of time these data were acquired. The comparison with the JM material is both

appropriate and striking.

Response: In the in-situ TEM experiment, it is different to obtain clear TEM images with high resolution since the sample continuously moves in the field of view during the heating process. Thus, a video is captured to record the structure change of nanoparticles during the in-situ TEM characterization with a heating rate of 1℃/min from room temperature to 1000 ℃. The TEM images used in **Supplementary Figure 6 and 8** are screenshots from the captured video, thus resulting in the resolution of the figures is not high. Even so, it can still demonstrate the thermal stability of nanoparticles (Pt/B/C) when compared with the commercial Pt/C(JM) catalysts. Besides the in-situ TEM experiment, we also performed a series of ex-situ heating experiments in which the sample of Pt/B/C was heated for 2h at a temperature from 200 to 1000 ℃ (**Figure R1-8**) as mentioned in response 5. These two experiments provide conclusive evidence that Pt/B/C has impressive thermal stability, opening up the possibility for further preparation of small-sized Pt-based intermetallic nanoparticles.

7.The boron XPS data appear to be severely overfitted, and not useful in my interpretation of the results. Is there a reason why the signal is so poor for the Pt/B sample? More detail must be given towards exactly which sample is being measured in the XPS. More experimental details about the XPS must also be given. Please also show the survey spectra in the SI.

Response: Thanks for the comments from the reviewer. The boron XPS data indeed shows a poor signal in Pt/B sample because of the relatively low content and low weight. From the preparation process, first HB and carbon were mixed to form the HB/C, then Pt^{4+} was added to form Pt/B/C. So, we measured the XPS spectra of HB/C and Pt/B/C samples to analyze the status of boron (**Figure R1-9**). The results show that the signal of B enhanced after adding C, demonstrating that C could stabilize B.

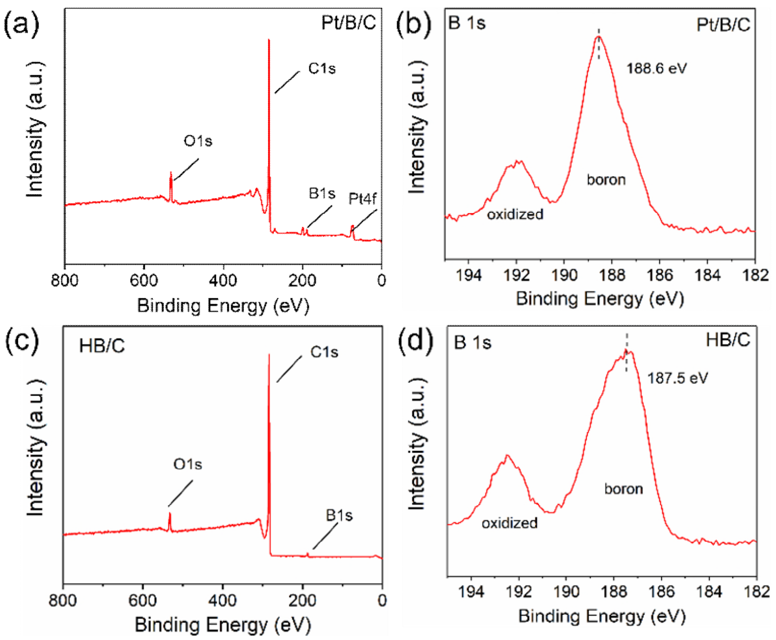

Figure R1-9. (a-b) XPS survey spectra and B 1s spectra of Pt/B/C. (c-d) XPS survey spectra and B 1s spectra of Pt/B/C.

Sample information and XPS measurement details: For the HB/C sample, HB is firstly mixed with ketjen carbon black in methanol via thorough stirring for 5 h to form the HB/C compound, then the compound was evaporated on an oil bath at 343 K. The powder was dried in vacuum oven overnight and pressed on the tape for XPS test. For Pt/B/C sample, HB is firstly mixed with ketjen carbon black in methanol via thorough stirring for 5 h to form the HB/C compound, then the precursor salt of Pt dissolved in methanol was added to the above suspension and stirred for three days. The precipitates were centrifugally collected and then washed with deionized water before dried in vacuum oven. Finally, the powder was pressed on the tape for XPS test.

Modification in the revised manuscript:

(1) line 204

X-ray photoelectron spectroscopy (XPS) was used to analyze the interaction between Pt and B. Fig. 2a presents the XPS survey spectra of the Pt/B/C sample, where distinct Pt 4f and B 1s signals can be resolved from the spectrum. The high-resolution XPS of Pt 4f show that the binding energy (BE) of Pt 4f shifts to higher value relative to the

reference metal Pt[36], demonstrating the potential electron transfer from Pt to B. The B 1s obtaining from the sample of Pt/B/C and spectra of HB/C are shown in Fig. 2c-d and Supplementary Fig. 16, respectively. The binding energy higher than 190 eV is attributed to B-O bonds, and the bonds present between 187.5 eV and 189 eV are attributed to B-B bonds [19,23,37]. Compared to the B1s peak position in HB/C, the B1s peak in Pt/B/C samples exhibits a positive shift of approximately 1.1 eV. This trend is consistent with the results reported by Kawamura et al.[19], which demonstrated a similar upward shift in the B1s peak position after UV-irradiation-induced H_2 release from HB. It suggests that the consumption of active H in HB by Pt^{4+} also leads to a decrease in electron density around the B atom, causing the observed positive peak shift.

(2) Figure 2 has been updated.

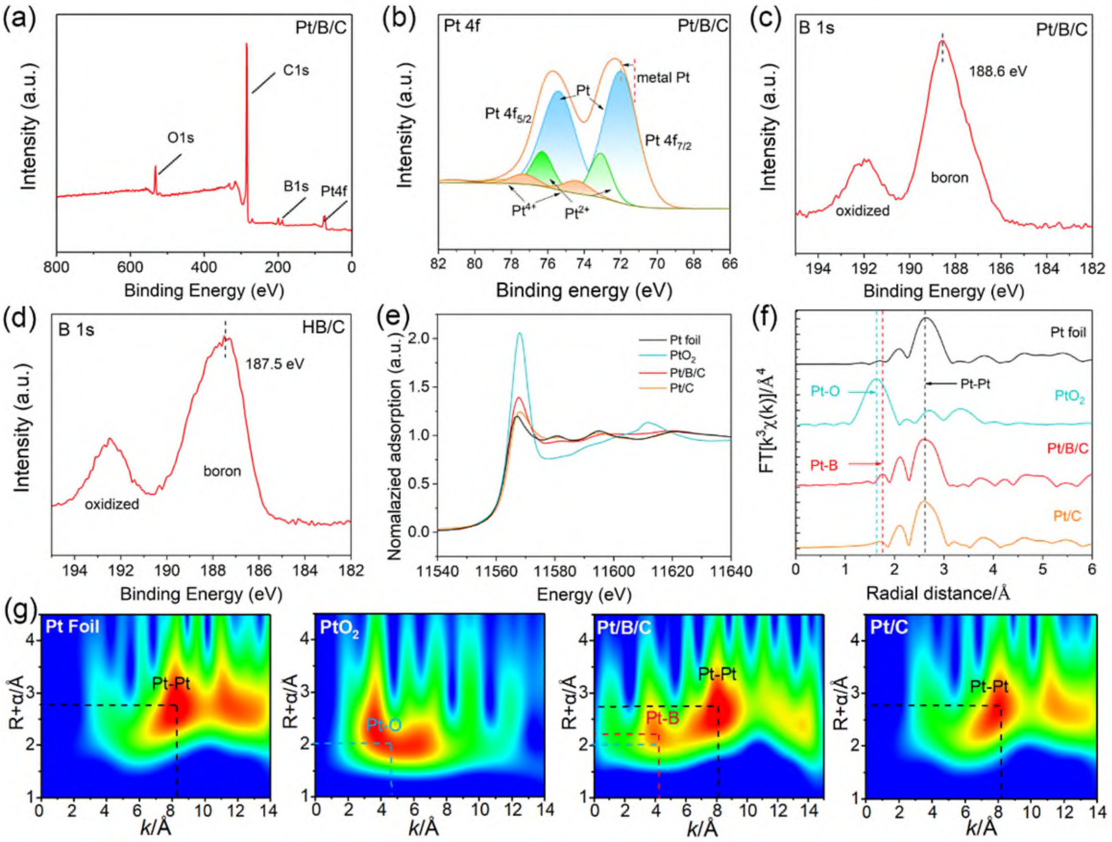

Fig.2 | Interaction of Pt-B analyzed in the experiment. (a) survey spectra XPS of Pt/B/C. (b) Pt 4f XPS of Pt/B/C. (c) B 1s XPS of Pt/B/C. (d) B 1s XPS of HB/C. (e) Pt L_3-edge XANES spectra of Pt/B/C, along with PtO_2, Pt foil and commercial Pt/C. (f) Pt L_3-edge EXAFS spectra of Pt/B/C, with PtO_2, Pt foil and commercial Pt/C. (g) WT-

EXAFS of the Pt L_3-edge signal of the Pt foil, PtO_2, Pt/B/C and commercial Pt/C.

(2) line 459

Characterizations. XPS was carried out on a Thermo Fisher Scientific ESCALAB Xi+ spectrometer with an Al Kα radiator. For the HB/C sample, HB is firstly mixed with ketjen carbon black in methanol *via* thorough stirring for 5 h to form the HB/C compound, then the compound was evaporated on an oil bath at 343 K. The powder was dried in vacuum oven overnight and pressed on the tape for XPS test. For Pt/B/C sample, HB is firstly mixed with ketjen carbon black in methanol *via* thorough stirring for 5 h to form the HB/C compound, then the precursor salt of Pt dissolved in methanol was added to the above suspension and stirred for three days. The precipitates were centrifugally collected and then washed with deionized water before dried in vacuum oven. Finally, the powder was pressed on the tape for XPS test.

8. The XANES/NEXAFS is interesting, and does show differences between the Pt foil and the Pt/B sample. However, the real comparsion we are interested in is the Pt/C sample. The wavelet analysis certainly shows a peak which I agree could be a Pt-C or Pt-B length. However the text interprets this as the formation of a bond, which in my opinion is not supported or especially useful here. When you adsorb atoms (from ALD, for example) onto any catalyst support, you will see the associated internuclear distances in the EXAFS. However, these are simply distances, and not necessarily bonds. This is why the Pt supported on a traditional carbon support is a much better comparison, since we don't usually speak of Pt/HSAC as exhibiting organometallic Pt-C bonds. In the Fig 3 analysis of the PtCo@B/C, the Pt-C correlation length is noticeably absent from the labelling. XANES/NEXAFS

Response: Thanks to the reviewer's comment on our XANES/NEXAFS result. We agree with the reviewer's analysis and added the XAS data of Pt/B/C and commercial Pt/C.

Since the main coordination element in all samples is Pt and the oscillation interval

of EXAFS on the XAFS map is mainly focused on the high *k* region, the Fourier transform weight in the FT-EXAFS map is raised from k^2-weight to k^3-weight.

From the EXAFS of Pt/B/C, the apparent peak located at 1.76 Å attribute to Pt-B bond and no sign of Pt-O or Pt-C occur. No Pt-C length was found in Pt/B/C, PtCo/B/C and commercial Pt/C samples (Fig. 2 and Fig. 3) Due to the small bond length of the Pt-C bond and low concentrations, only Pt-Pt but no Pt-C neighbors were observed in pre-reduced Pt/C samples, which would be expected as the background (from slowly changing signal of radiation generated by the blank area between Pt core and coordination atom) removal by AUTOBK Algorithm method (*Jiang Guo Ping and Harry E. Ruda Phys. Rev. B 47, 1412*), consistent with test results of similar material systems. So, we did not find any peaks that could be attributed to Pt-C in XAFS, but observed a slight increase in the white line peak of Pt/C samples compared to the Pt foil, indicating a slight interaction between Pt and C. Therefore, the characteristic peak of commercial Pt/C sample is not observed in the experiment, which is consistent with the literature (*Phys. Chem. Chem. Phys., 2009,11, 5773-5781*). For PtCo sample, there were three scattering pathes (Pt-Pt, Pt-Co, and Pt-O) in EXAFS and no Pt-C scattering was observed.

Modification in the revised manuscript:

(1) Figure 2 and Figure 3 has been updated.

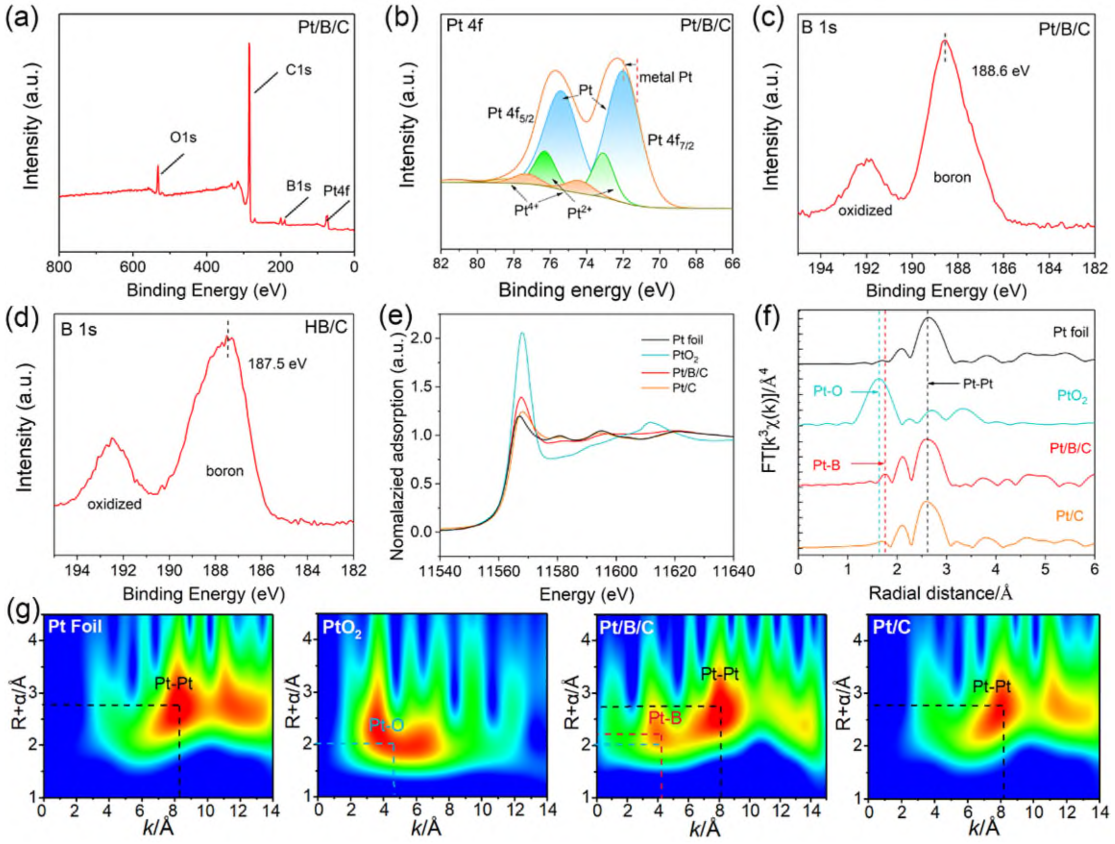

Fig.2 | Interaction of Pt-B analyzed in the experiment.(a) survey spetra XPS of Pt/B/C. (b) Pt 4f XPS of Pt/B/C. (c) B 1s XPS of Pt/B/C. (d) B 1s XPS of HB/C. (e) Pt L_3-edge XANES spectra of Pt/B/C, along with PtO_2, Pt foil and commercial Pt/C. (f) Pt L_3-edge EXAFS spectra of Pt/B/C, with PtO_2, Pt foil and commercial Pt/C. (g) WT-EXAFS of the Pt L_3-edge signal of the Pt foil, PtO_2, Pt/B/C and commercial Pt/C.

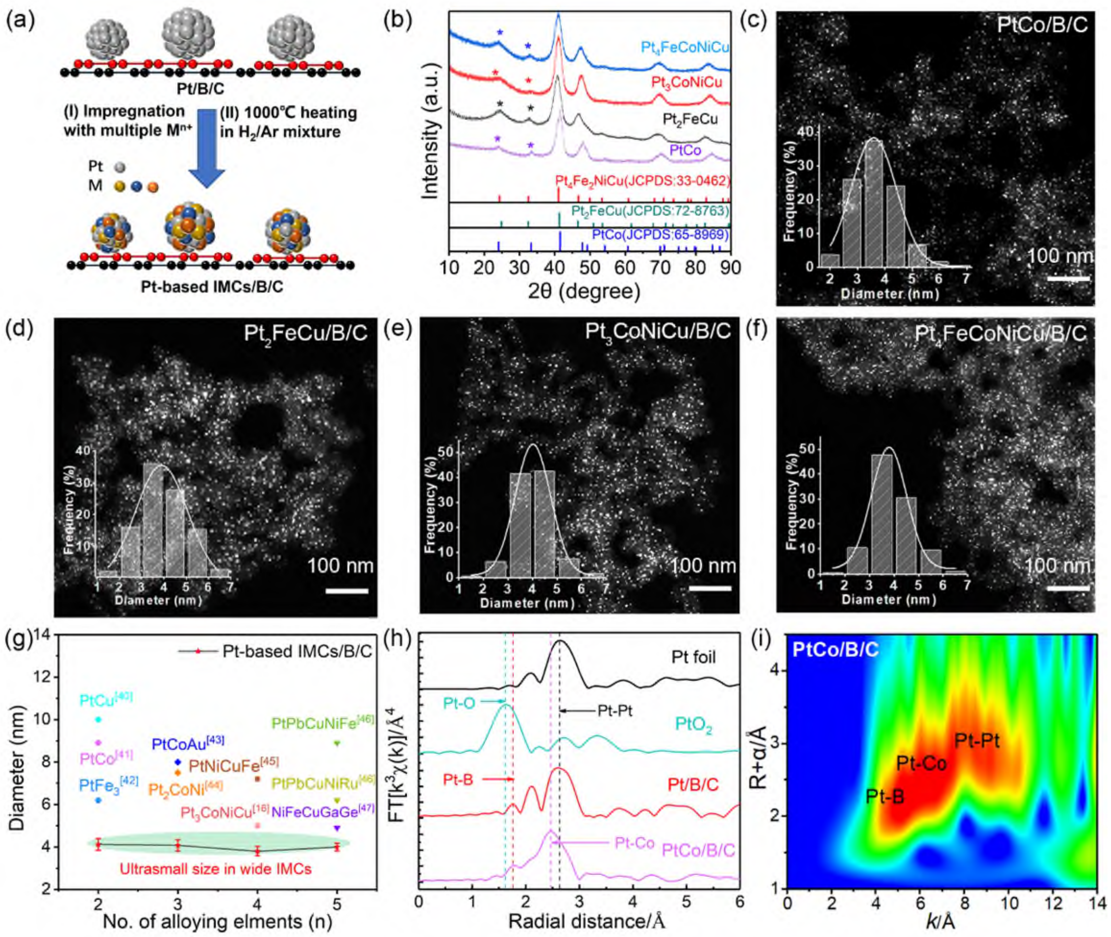

Fig. 3 | Synthesis of ultrafine Pt-based IMCs based on Pt/B/C (a) Schematic illustration of the process of Pt-based multielement intermetallic catalysts. (b) Powder XRD patterns of Pt-based intermetallic alloy. (c-f) HAADF-STEM images and size distribution of intermetallic alloy PtCo/B/C, Pt_2FeCu/B/C, $Pt_3CoNiCu$/B/C, $Pt_4FeCoNiCu$/B/C. (g) Comparison of intermetallic alloy size between our work with the state-of-art in the literature. (h) Pt L_3-edge EXAFS spectra of PtCo/B/C, along with Pt/B/C, PtO_2 and Pt foil in *R* space. (i) WT-EXAFS of the Pt L_3-edge signal of PtCo/B/C.

9. The temperature and treatment conditions for the alloy samples needs to much more precisely detailed. It is difficult, for example, to understand what sample Fig S19 represents. How it was annealed? What is the support?

Response: Thanks for the comments from the reviewer. For **Supplementary Figure 19,** the metal salts are adsorbed by ketjen black (KB), followed with annealing at

1000°C for 1 hour and then annealed at 600°C for 6 hours at 5% H_2/Ar atmosphere. The heating rate for each stage is 5 °C min^{-1}. We have added the synthesis details of alloy sample in the experiment section.

Modification in the revised manuscript:

(1) line 430

Synthesis of Pt-based IMCs. 100 mg Pt/B/KB powder was dispersed in 50 mL deionized water. Then, the transition metal precursor with a specific mole ratio was added to the dispersion with continuous stirring in a water bath at 60 °C until the solution was evaporated. The obtained powder was ground and annealed at a one or two-step high temperature (for one step, each temperature (600-900 °C) held 2 h. For two steps, 1000 °C for an hour and then annealed at 600 °C for 6 h) in 5% H_2/Ar atmosphere with the heating rate of 5 °C min^{-1} and then cooled to room temperature naturally. Finally, the as-prepared alloys were treated in 0.1 M $HClO_4$ at room temperature for 2 h, followed by an annealing treatment in a 5% H_2/Ar at 400°C for 2 h. (labeled PtM/B/C).

(2) line 439

Synthesis of Pt-based alloys without B. For the PtCo sample, $H_2PtCl_6{\cdot}6H_2O$, $Co(NO_3)_2{\cdot}6H_2O$, and KB were dispersed into 50 mL DI-water (Pt/Co mole ratio 1:1 and Pt content to be 20 wt.%) under ultrasonic for 2 h. After vacuum drying and grounding, the powder was subjected to heat treatment at 1000°C for 1 hour and then annealed at 600°C for 6 hours in a 5% H_2/Ar atmosphere with a heating rate of 5 °C min^{-1}. Finally, the powder was treated in 0.1 M $HClO_4$ at room temperature for 2 h, followed by an annealing treatment in a 5% H_2/Ar at 400°C for 2 h. The synthesis of $Pt_4FeCoNiCu$ was similar to that of PtCo, except for varying the added amount of transition metal salt and keeping the mole ratio of Pt/Fe/Co/Ni/Cu at 4:1:1:1:1.

10. The images in Fig 3 c-f are much too low resolution to understand the particle size distributions shown as inset.

Response: Thanks for this comment. The reason to show the HAADF-STEM images with relatively low magnification in Figures 3c-f is to enable readers to observe the uniform distribution of nanoparticles in a large field of view, which is conducive to understanding the particle size distribution over a large range and that our process did prevent nanoparticle sintering during the high-temperature intermetallic formation process. According to the reviewer's suggestion, the enlarged HAADF-STEM images as shown in **Figure R1-10** were also provided in the **supplementary information**.

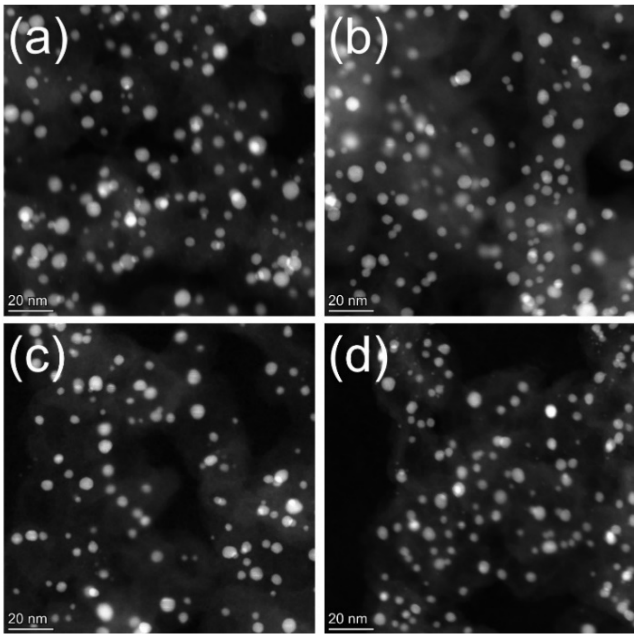

Figure R1-10. High-resolution HAADF-STEM images of intermetallic alloy PtCo/B/C, Pt_2FeCu/B/C, $Pt_3CoNiCu$/B/C, $Pt_4FeCoNiCu$/B/C, respectively.

Modification in the revised manuscript:

(1) line 268

HAADF-STEM images obtained from the aforementioned samples confirm the uniform distribution of both binary, ternary, quaternary, and quinary IMCs nanoparticles on the carbon support with narrow size distributions (Fig. 3c-f and Supplementary Fig. 26).

(2) **Figure R1-10** was listed in revised **Supplementary Information** as **Supplementary Fig. 26.**

Reviewer #2:

The authors propose utilizing highly reductive hydrogen borophene (HB) to produce an electrocatalyst characterized by small size, high-density dispersion, and robust stability, which is primarily attributed to the formation of the Pt-B bond. Moreover, they have successfully demonstrated the synthesis of a wide array of ultrafine binary, ternary, quaternary, and quinary intermetallic catalysts using the Pt@B module. These catalysts exhibit remarkable stability, resisting particle sintering even when annealed at 1,000°C. The issue of maintaining particle size during heat treatment is of paramount importance when it comes to producing thermodynamically stable alloys, given that an increase in catalyst loading may induce severe sintering via particle coalescence. As the authors present a novel strategy characterized by a dependable morphology, this reviewer recommends the manuscript for publication in Nature Communications, once the remaining issues have been addressed.

Response: We thank the reviewer for thinking highly of our work about the novel strategy to stabilize metal with high stability, and sincerely appreciate the reviewer for his/her valuable comments which have made a tremendous improvement to our manuscript.

Comment 1. *The authors have loaded the Pt@B module onto the support. How can it be confirmed whether Pt nanoparticle adheres to the support or B is loaded onto the support? Isn't there a possibility that B covering the surface of Pt could prevent sintering?*

Response: We are grateful for the reviewer's comment about the refinement of the sintering resistance mechanism Since boron is challenging to probe with electron microscopy or even X-ray techniques, the revised draft is mainly from the preparation process and some control experiments to demonstrate the Pt/B/support model

<u>Sample preparation process.</u> First, for the preparation of Pt/B/C catalysts, HB was firstly mixed with Ketjen carbon black to form HB/C composite support structure; Pt

precursor was then added to the HB/C composite support and reduced by HB to form Pt/B/C catalysts (**Figure R2-1**). Since Pt is lastly added to HB/C and was in situ reduced by HB to form Pt nanoclusters, it is unlikely that Pt nanoclusters were physically covered by the HB or Boron sheets to induce any severe physical covering effect.

Below is the detailed sample preparation process. HB and carbon black were mixed in methanol *via* thorough stirring for 5 h to form the HB/C compound suspension by taking advantage of the strong adsorption of HB with a 2D morphology to carbon black. The precursor salt of Pt dissolved in methanol is then added to the above suspension and stirred for three days. During such a process, the Pt ions were reduced to Pt (0) by HB. The following growth of Pt nanoparticles was on the basis of the in-situ formed Pt clusters that have been already anchored on the surface of C through B layer. Therefore, from the view of the preparation process, the Pt nanoparticles loaded on C is unlikely to be coated by the B-layer.

Control sample B/Pt/C. Second, a key control sample (B/Pt/C) was also synthesized and compared with Pt/B/C to indirectly demonstrate that B was not coated on the surface of Pt nanoparticles in our Pt/B/C. To illustrate the B physical covering effect, we purposely synthesized B/Pt/C by covering the Pt/C with HB or Boron layers. Briefly, 20 wt.% commercial Pt/C(JM), which has a similar particle size to our Pt/B/C catalyst (**Figure R2-2a and 2b**), was mixed with 5 mL methanol suspension of HB by thoroughly stirring for 1 day. And then, 2 mL DI water was added to the above mixture, stirring for 1 hour to promote the hydrolysis of HB and Boron layer coating. It is obvious that the B layer, which is derived from the hydrolysis of HB, would be coated on the surface of Pt nanoparticles in JM catalyst (denoted as B/Pt/C(JM)).

Figures R2-2c and 2d show the cyclic voltammetry (CV) curves and CO stripping of B/Pt/C(JM) - B physical covering, Pt/C(JM) – no B, and Pt/B/C – our catalysts, respectively. It is evident that the integrated area of B/Pt/C(JM) sample in both H_2 underpotential deposition (HUPD) and CO stripping regions drops sharply in contrast to that of the initial Pt/C(JM), while our Pt/B/C sample presents the highest specific surface area among these samples (**Table R2-1**). Considering Pt nanoparticles are similar (size and loading) in these three samples, such a drastic change in surface

exposure implies different surface structures: the smallest ECSA in B/Pt/C should be ascribed to the B physical coating layer on Pt nanoparticles, which further proves that the Pt nanoparticle in our Pt/B/C was mostly exposed and not covered by B.

From reviewer's comments, we noticed that the naming of Pt@B might cause misunderstanding to readers about the structure of the as-prepared samples, because A@B usually means a core-shell structure. Therefore, we have replaced the Pt@B to Pt/B in the revised manuscript.

Furthermore, the XAS data of Pt/B/C was also shown. Since the main coordination element in all samples is Pt and the oscillation interval of EXAFS on the XAS map is mainly focused on the high *k* region. So, the Fourier transform weight in the FT-EXAFS map is raised from k^2-weight to k^3-weight to increase the resolution of peak. From the EXAFS of Pt/B/C, we can see that an apparent peak in 1.78 Å which attribute to Pt-B bond and no sign of Pt-O or Pt-C occur comparing to Pt/C and PtO_2. In addition, the fitting results also indicates that the existence of Pt-B bond in Pt/B/C sample and has a bond length 2.22 Å (**Figure R2-3** and **Table R2-2**).

In summary, we believe that Pt load on B/C to form Pt/B/C structure, rather than B covering the surface of Pt (B/Pt/C) to prevent sintering.

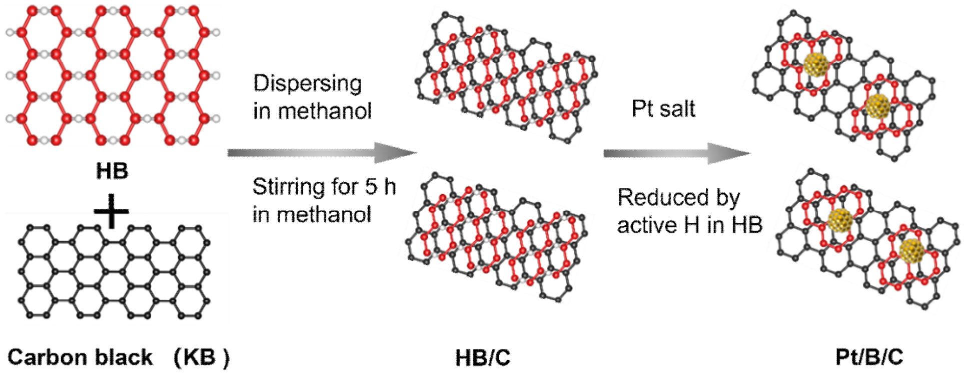

Figure R2-1. Schematic diagram of synthesis process of Pt/B/C.

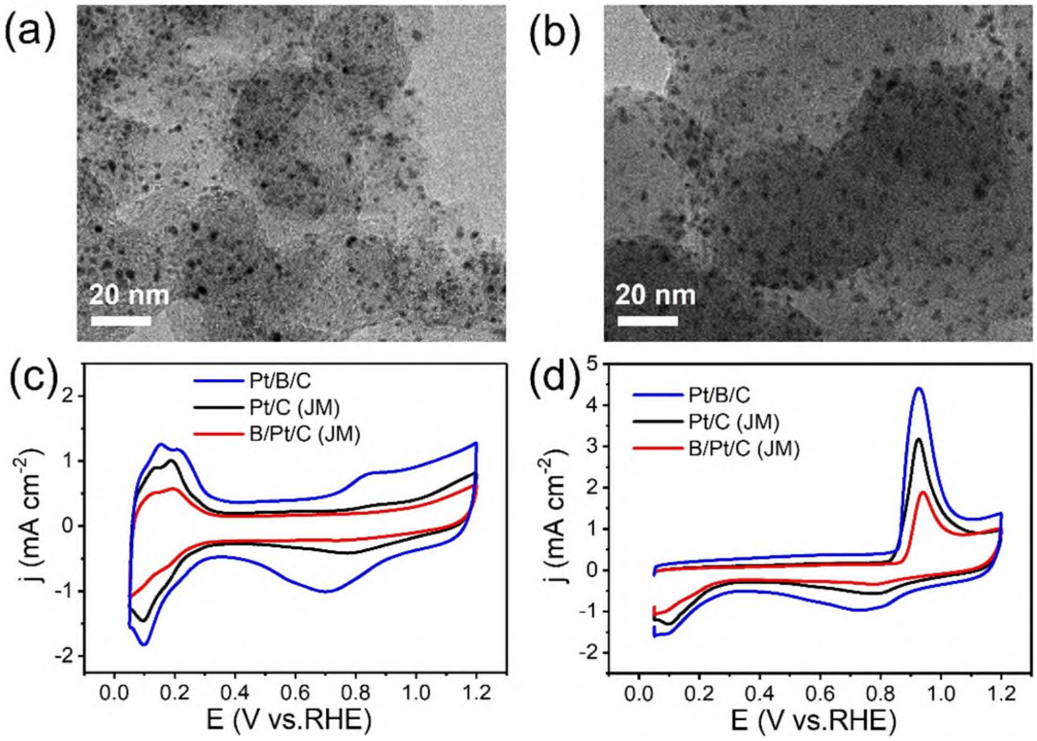

Figure R2-2. (a- b) TEM of Pt/B/C and commercial Pt/C (JM) catalyst; (c-d), CVcurves of and CO-stripping of Pt/B/C (as-prepared), commercial Pt/C (JM) and B/Pt/C (JM).

Table R2-1. ECSA of three samples calculated by different methods.

	Pt/C	B/Pt/C	Pt/B/C
$ECSA_H/m^2g_{Pt}^{-1}$	61.5	31.1	74.5
$ECSA_{CO}/ m^2g_{Pt}^{-1}$	69.8	33.8	92.3

Test conditions: CV curves of these samples were recorded in N_2-saturated electrolyte at a scan rate of 50 mV s^{-1}; CO stripping was carried out by first holding the catalyst electrode at 0.06 V in CO-saturated 0.1 M $HClO_4$ for 15 min. Subsequently, N_2 was purged through the electrolyte for 20 min to remove residual CO and then the electrode was scanned from 0.06 to 1.0 V at 100 mV s^{-1} to record the CO stripping voltammogram. Production process, Pt/C: purchased; Pt/B/C: as-prepared; B/Pt/C: Pt/C was mixed with 5 mL methanol suspension of HB by thoroughly stirring for 1 day. And then, 2 mL DI water was added to above mixture, stirring for 1 hour to promote the hydrolysis of HB before drying in vacuum oven.

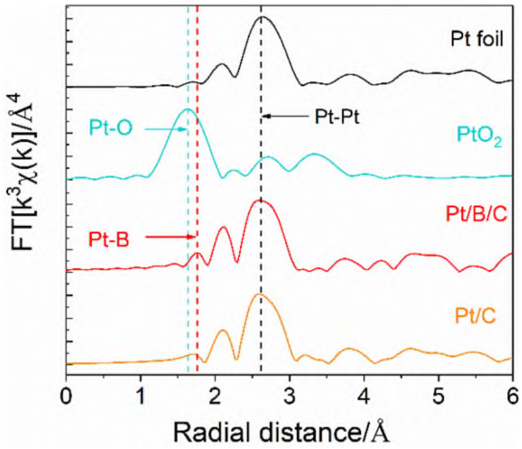

Figure R2-3. EXAFS spectra at Pt L3-edge for for Pt foil, PtO_2, Pt/B/C, commercial Pt/C in R space.

Table R2-2. Structural parameters extracted from EXAFS fitting at Pt L_3-edge of Pt foil, PtO_2, Pt/B/C, Pt/B in R space.

Sample	Path	CN	R(Å)	σ^2*10^{-3}(Å)	ΔE_0(eV)	R-factor
Pt foil	Pt-Pt	12.00	2.76	-	-	-
PtO_2	Pt-O	6	2.02	-	-	-
Pt/B/C	Pt-Pt	4.71±0.39	2.75±0.02	6.52±0.62	9.05±1.03	0.010
	Pt-B	1.23±0.11	2.22±0.05	5.90±0.75	-11.38±1.75	

*CN, coordination number; R, distance between absorber and backscatter atoms; σ^2, change value of Debye-Waller factor relative to the reference compound; ΔE_0, inner potential correction between the sample and the reference compound. Error bounds (accuracies) of the structural parameters are estimated to be: CN ±20%; R, ±2%; $\Delta\sigma^2$, ±20%; and ΔE_0, ±20%.

Modification in the revised manuscript:

(1) line 118

In the experiment, we conducted a two-step synthesis procedure to fabricate the catalyst, as illustrated in Figure 1a. Firstly, the as-prepared HB was mixed with activated carbon

to form HB/C support. Subsequently, a solution containing H_2PtCl_6 was introduced, where the Pt^{4+} ions was then reduced by the bridging hydrogen atoms in HB, along with the split of hexagonal boron network, due to the breakup of the terminal B-H bond. The following growth of Pt nanoparticles was on the basis of the in-situ formed Pt clusters that has been already anchored on the surface of C through B layer.

(2) line 133

Our additional experiments have revealed that, in contrast to the encapsulation mechanism often reported in the literature[31], this stabilization effect is achieved by employing B fragments as a stabilizing medium between activated carbon and Pt nanoparticles (See Supplementary Fig. 4 and Table 1).

(3) line 234

Fourier transform of EXAFS data in R space is shown in Fig. 2f. Compared to Pt-O bond (located at 1.63 Å) in PtO2, the apparent peak located at 1.78 Å can be assigned to Pt-B scattering. Subsequent fitting results further confirmed the existence of Pt-B bond in Pt/B/C with a bond length of 2.22 Å (Supplementary Fig. 17 and Table 2), consistent with the computation model after relaxation in DFT calculation (Supplementary Fig. 14). Furthermore, the corresponding wavelet transformed (WT)-EXAFS analysis was performed, which is a precise approach to distinguish the backscattering atoms even though they overlap substantially in R space. As shown in Fig. 2g, the Pt/B/C sample exhibits two maximums on y axis, one is located at *ca.* 2.8 Å which is ascribed to the Pt-Pt coordination, consistent with Pt foil reference, and the other (R+α: *ca.* 2.2 Å) which is different from Pt-O (R+α: *ca.* 2 Å) in PtO_2 reference can be assigned to the Pt-B bond. The combination of these analyses conclusively demonstrates that the Pt is loaded on borophene, enhancing the adhesive energy between nanoparticles and the B/C support while suppressing aggregation and sintering of Pt nanoparticles at elevated temperatures.

(4) Fig. 2 has been updated.

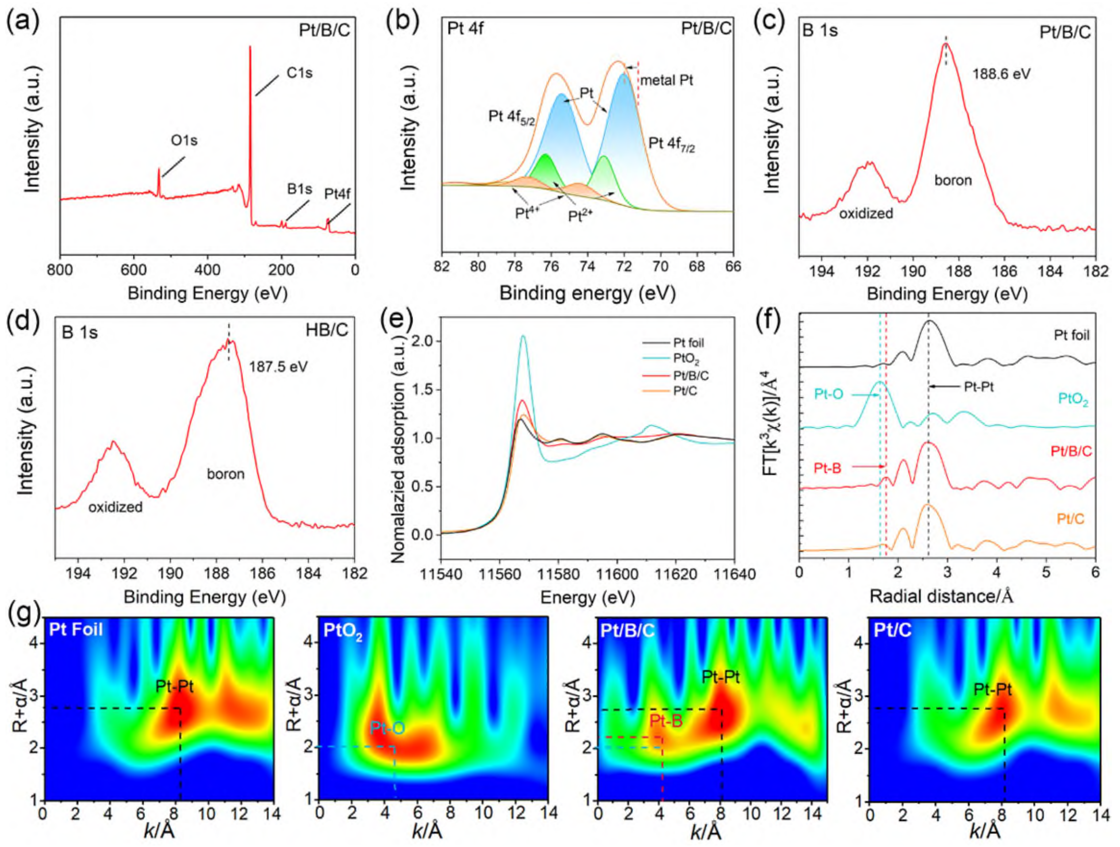

Fig.2 | Interaction of Pt-B analyzed in the experiment.(a) survey spetra XPS of Pt/B/C. (b) Pt 4f XPS of Pt/B/C. (c) B 1s XPS of Pt/B/C. (d) B 1s XPS of HB/C. (e) Pt L_3-edge XANES spectra of Pt/B/C, along with PtO_2, Pt foil and commercial Pt/C. (f) Pt L_3-edge EXAFS spectra of Pt/B/C, with PtO_2, Pt foil and commercial Pt/C. (g) WT-EXAFS of the Pt L_3-edge signal of the Pt foil, PtO_2, Pt/B/C and commercial Pt/C.

Modification in the Supplementary Information:

(1) **Figure R2-2** was listed in revised **Supplementary Information** as **Supplementary Figure 4.**

(2) **Table R2-1** was listed in revised **Supplementary Information** as **Supplementary Table 1.**

(4) **Table R2-2** was listed in revised **Supplementary Information** as **Supplementary Table 2.**

2. There will be lattice mismatching between B and the support. And this mismatching will likely affect the electronic and geometric structure of Pt and B. It might be

beneficial to discuss the implications of this. Such explanation is missing in the DFT model.

Response: Thanks for the comment about the relationship between B and support. We admit that there indeed have a relationship between B and C, which was also reported in previous article (The bonding energy between the graphene and borophene layers at the equilibrium position was reported higher than that between the graphene layers (-0.035 eV/atom vs. -0.02 eV/atom) *Tribology Letters* **69**, 44 (2021)). However, what we pay attention is the interaction of Pt-B *vs*. Pt-C, which provide an effort to stabilize Pt. Through calculating the escape energy of the atom in Pt_{13} which is furthest from the substrate (graphene or borophene), we found the escape energy of individual atoms from Pt_{13} cluster on borophene (4.68 eV) was obviously larger than that on graphene (3.16 eV). Compared to the interaction of Pt-B *vs*. Pt-C, the interaction of B-C show a negligible effect.

3. In the XPS data, the binding energy of Pt 4f has increased, which typically signifies a decrease in oxygen adsorption strength due to a d-band downshift. However, the authors explain this trend in reverse. The transfer of electrons from Pt to B implies a decrease in activity due to an upshift of the d band. An explanation is needed for this.

Response: Thanks for the comment about the XPS of Pt/B. The binding energy of Pt 4f increases indeed in our XPS data which will decrease the activity as mentioned by the reviewer. Such a decrease in activity was generally attributed to the upshifts of the d-band center.

However, in our current work, Pt/B is just used as an intermediate for further preparation of intermetallic alloys. What we discussed more is the ORR performance of these PtM intermetallics rather than Pt/B. The PtM/B catalysts show highly enhanced ORR catalytic performances mainly due to alloying Pt with various metal elements and forming intermetallics. Therefore, compared with the effect of B-C electron transfer on the activity, the alloy composition, the intermetallic structure, and the small particle size

all have more significant contributions to the improvement of their performance.

4. The changes in Pt's electronic structure induced by B are likely to result in changes in activity and durability. I recommend adding DFT results for this and discussing it alongside physicochemical analyses, including XPS and EXAFS.

Response:

We agree with the reviewer that Pt's electronic structure could be induced by B, and then result in changes in activity and durability. As shown in **Figure R2-4 and R2-5**, an obvious change of Pt's electronic was observed in the XPS and XAS of Pt/B/C compared to Pt foil, and the PtM/B/C alloys show a better activity and durability than commercial Pt/C in ORR test (Fig. 5). However, what we focus on is a hydrogenated borophene (HB)-triggered synthesis method for multielemental Pt intermetallic compounds (IMCs) and the sintering resistance mechanism of IMCs through a series characterization. As for the effect of Pt induced by B, we do not discuss more. In addition, for the system of PtM/B/C, it is quite difficult to build a calculation model because of the complexity of the system, such as larger particles (4-5 nm), core-shell structure and multielement. The aim of our current work is to advance an anchoring strategy to suppress the growth of PtM intermetallic nanoparticles during the high-temperature annealing process. Nevertheless, as mentioned by the reviewer, it is also an important issue of Pt's and PtM's electronic structure that induced by B to result in changes in activity and durability. In our further study, we will focus on this issue.

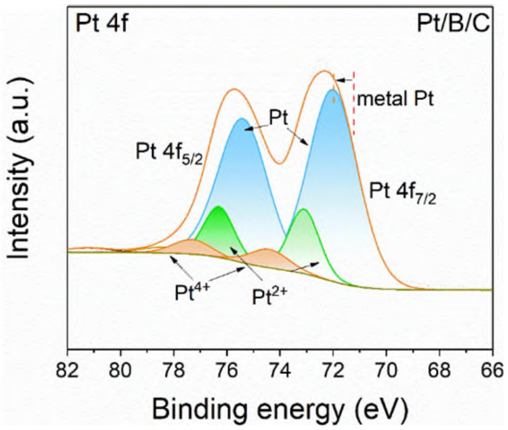

Figure R2-4. Pt 4f XPS of Pt/B/C

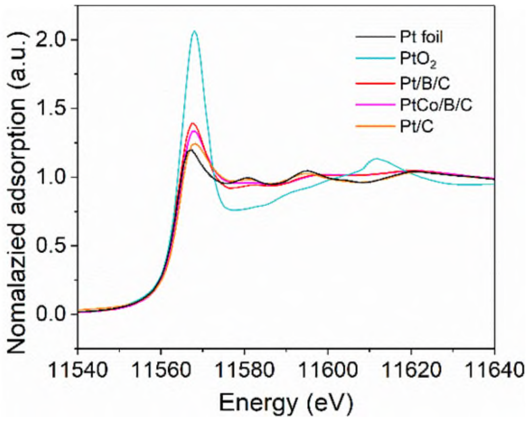

Figure R 2-5 . Pt L_3-edge XANES spectra of Pt/B/C, PtCo/B/C along with PtO_2, Pt foil and commercial Pt/C.

5. The double layer of the catalyst with the PtB module appears very wide. Materials with a wide double layer often exhibit low electrochemical reactivity. It is necessary to discuss whether the presence of B could potentially act as a resistance to activity (excluding changes in the catalyst's electronic structure) and whether it possesses adequate electrical conductivity.

Response: We are grateful for the reviewer’s comment on the detail about the double layer of the catalyst with the Pt/B/C module. Compared to commercial Pt/C, our Pt IMCs catalysts synthesized with the Pt/B/C module show indeed a wider double layer which may affect performance to some extent due to the existence of B. Even so, our catalysts still perform better than commercial Pt/C since many factors affect their

performance. Though B may not possess adequate electrical conductivity, it plays an important role in the high-temperature resistance to sintering, which induces a relatively smaller size of PtM intermetallic nanoparticles and a higher mass activity.

6. Writing out the full name of IMCs when it first appears would be helpful for the readers to understand.

Response: Thanks reviewer's comment on details and we have revised it. In addition, we also checked the full manuscript again.

7.The Pt/C ORR curve appears to be underestimated compared to previous reports. Verification is necessary. Using an Ag/AgCl electrode as a reference electrode may not be appropriate. Cl contamination can interfere with the ORR reaction of Pt, leading to potential underestimation. Additionally, it is recommended to specify whether the data has been corrected for IR (Ohmic) drop in the experimental section.

Response: Thanks for the suggestion from the reviewer. According to the suggestion, we have retested the commercial Pt/C and modified the data, as shown in Figure 5 and SI. All the data has been corrected for IR (Ohmic) drop and we illustrate this in the experimental section.

Using an Ag/AgCl electrode as a reference electrode is based on a series of previously published articles. (*Joule, 2019, 3, 124-135; Nature Energy, 2021, 6, 614–623; Science, 2019,366,850-856; Nature Catalysis, 2022, 5,503–512; Adv. Energy Mater. 2023, 13, 2204257; Adv. Mater. 2023, 2302067; J. Am. Chem. Soc. 2022, 144, 19106–19114; Science, 2016, 354, 1414-1419; Angew. Chem. Int. Ed.2023,62, e2023044*).

In addition, we replaced the electrolyte immediately after testing each electrode. So logically, the Cl^- would have very little effect. According to the reviewer's comment, we matched a salt bridge (Luggin capillary) for the AgCl electrode as a reference electrode to reduce Cl^- contamination and finally obtained similar results. Therefore,

the effect from the Cl^- contamination should be within a reasonable range in our cases.

Correspondingly, the above experimental details are also described in the Supplementary Information.

Modification in the revised manuscript:

(1) line 491

All the data has been corrected with IR (Ohmic) drop. In addition, we replaced the electrolyte immediately after testing each electrode to avoid being contaminated.

(3) Figure 5 has been updated.

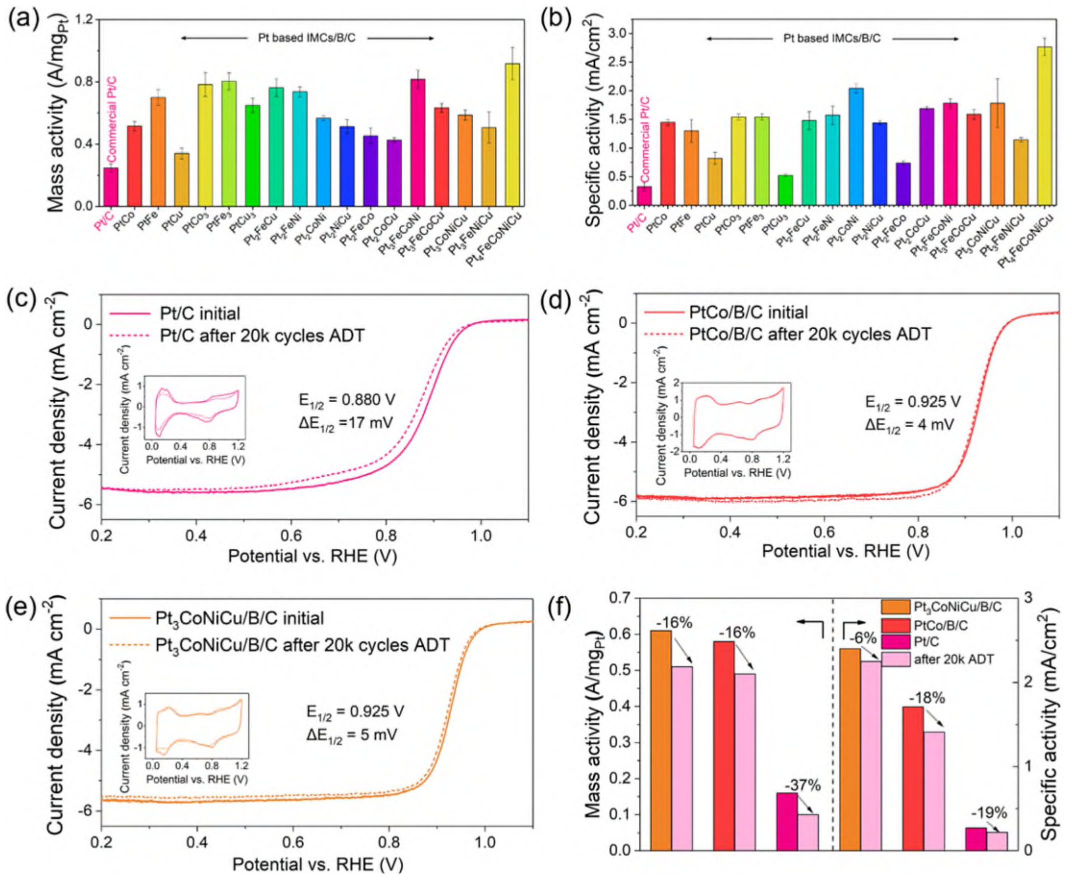

Modification in the Supplementary Information:

(1) Supplementary Figure 30 has been updated.

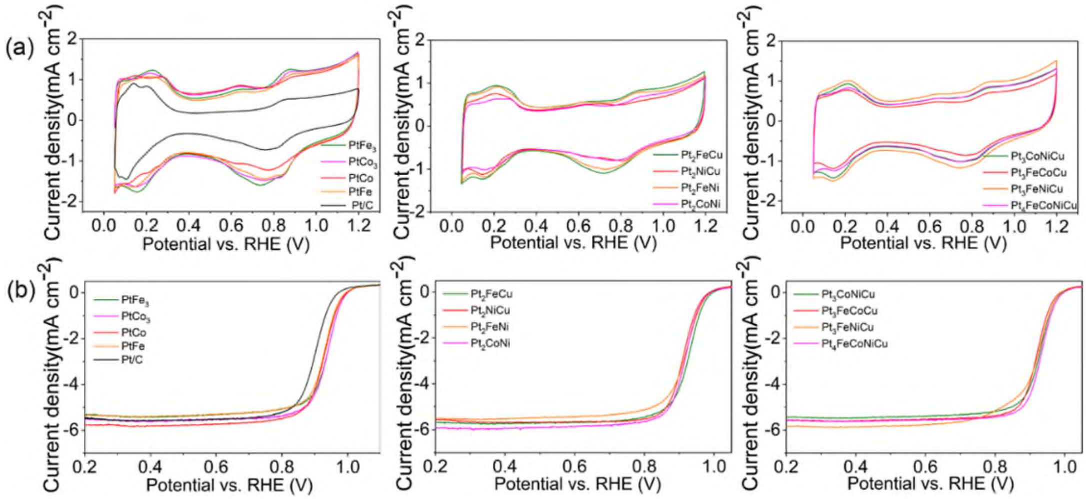

Supplementary Figure 30. (a) CV curves and (b) ORR polarization curves of Pt-based IMCs/B/C

Reviewer #3

Zeng et al. proposed to use borophene-stabilized strategy to synthesize Pt@B module catalyst, which can be further dispersed on various carbon and metal oxide-based supports. To show the application of this material, they studied electrocatalytic ORR as a demonstration. The emphasis of this work is the stability of materials. Here are questions regarding the stability of materials and the local structure of Pt@B.

Response: We thank the reviewer for carefully reading our manuscript and appreciating our work on the borophene-stabilized strategy with high versatility. We sincerely appreciate the reviewer for the valuable comments which have made a tremendous improvement to our manuscript.

Comment 1. *Based on Fig. 1 and Fig. S2, in Pt@B, the average size of Pt is about 2 nm. But after depositing Pt@B to other supports, the Pt size changes depending on the type of supports and the weight loading of Pt@B. Why would that happen if Pt has a strong interaction with B? Does that mean that after the addition of substrates, Pt could migrate to substrates?*

Response: We are grateful for the reviewer's comment on the particle size change towards support. This and the following comments may come from the confusion about the preparation of Pt/B/C catalysts and we are sorry for causing the misunderstanding.

The Pt/B was synthesized by directly adding an aqueous solution of H_2PtCl_4 into the methanol suspension of HB, and then the Pt^{4+} ions can be reduced to Pt^0 to form Pt nanoparticles which are in-situ loaded on the borophene sheet, as shown in **Figure 1b** (in the original manuscript). Meanwhile, the preparation process of Pt/B/support was shown in **Figure R3-1**. First, we mixed HB with support (such as carbon black, KB) and dispersed them in methanol by thoroughly stirring to form the HB/C compound suspension because of the strong adsorption characteristics of KB. Then, the precursor salt of Pt dissolved in methanol was added to the above suspension and stirred for three days. Therefore, the Pt ions were reduced by HB/C complex and in-situ loaded on B/C

substrate to form the Pt/B/C catalysts, rather than mixing the Pt/B with carbon.

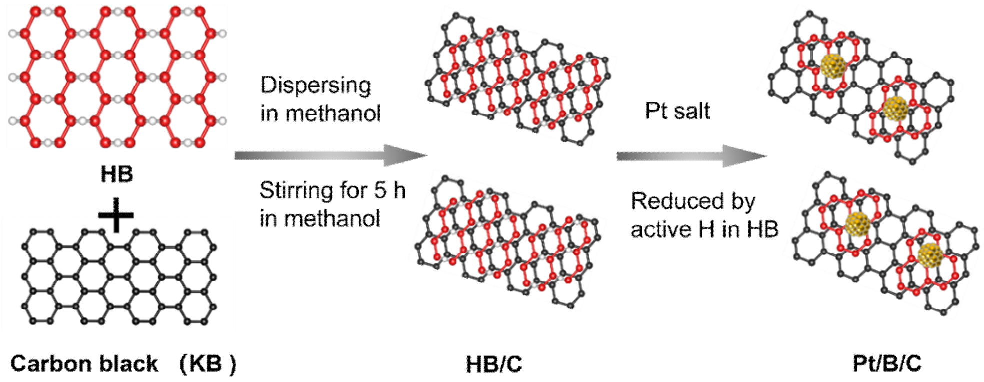

Figure R3-1. Schematic diagram of the synthesis process.

With the above experimental process in mind, we can understand the size change of the Pt nanoparticle depending on the type of supports and the weight loading. When the surface area of the support is not enough to disperse the Pt sufficiently, their sizes would increase due to the agglomeration of Pt cluster. We test the Brunauer Emmett Teller (BET) of different supports: The ketjen carbon black we used in our work has a surface area of ~1400 m^2/g, while the metal oxide has only ~ 250 m^2/g. Therefore, the size of Pt nanoparticles becomes larger with the decrease of the surface area of supports or increase of Pt loading. In addition, from both the experimental study and the DFT result, we have proved our catalysts are very stable after formation. Therefore, the size change of Pt comes from having different substrates and loading, not because Pt self-migrated after being loaded onto the substrates.

Comment 2. *Do you have evidence showing that Pt remains interacting with B after the addition of substrates?*

Response: We appreciate the reviewer's comment about if Pt remains interacting with B after the addition of substrates. As discussed above, from the perspective of the preparation process **(Figure R3-1)**, Pt was added to the HB/C support to be in situ reduced by the HB to form Pt/B/C catalysts, not Pt/B adding to C substrate. Again, we

are sorry to cause this misunderstanding of the preparation of Pt/B/C catalysts.

As for the evidence, first, the EDS mapping show that the B is mainly distributed in regions with Pt in Pt/B/C (**Figure R3-2**). Secondly, the Pt 4f XPS of Pt/B/C show a positive shift bonding energy compared to metal Pt, demonstating the interaction between B and Pt (**Figure R3-3**). Thirdly, the XANES of Pt/B/C show a different white line peak intensity of Pt compared to Pt foil and Pt/C, which is consistent with XPS. In addition, the EXAFS spectra at Pt L_3-edge of Pt/B/C indicate an apparent peak in 1.78 Å which attribute to Pt-B distance (**Figure R3-4)**. Finally, our DFT calculation results show that the interaction between Pt NPs and B layer is much stronger than that on carbon (graphene) as shown in **Supplementary Fig. 14**.

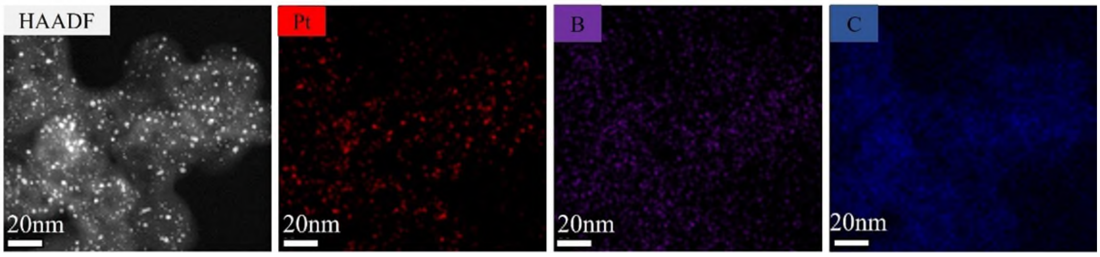

Figure R3-2. EDS-mapping of Pt/B/C.

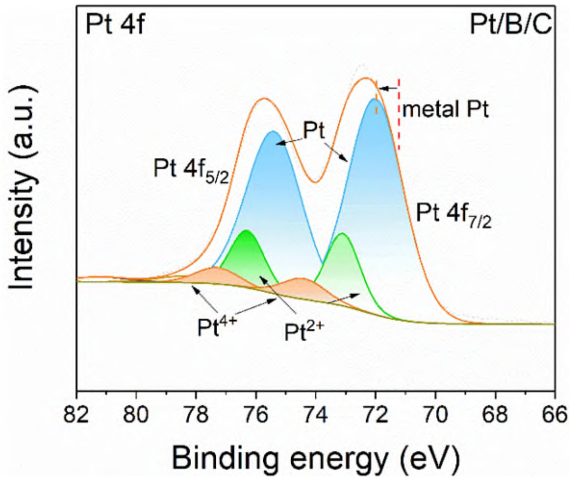

Figure R3-3. Pt 4f XPS of Pt/B/C.

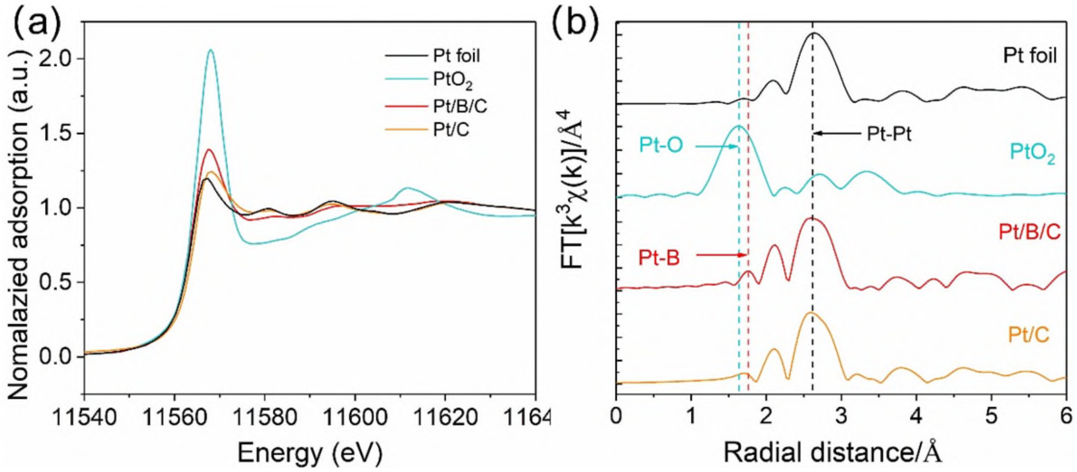

Figure R3-4. (a-b) XANES and EXAFS spectra at Pt L_3-edge for for Pt foil, PtO_2, Pt/B/C, commercial Pt/C in R space.

Therefore, we believe that Pt still remains interacting with B when it forms the Pt/B/C catalyst. Correspondingly, we have made some modifications in the revised manuscript.

Modification in the revised manuscript:

(1) line 118

In the experiment, we conducted a two-step synthesis procedure to fabricate the catalyst, as illustrated in Figure 1a. Firstly, the as-prepared HB was mixed with activated carbon to form HB/C support. Subsequently, a solution containing H_2PtCl_6 was introduced, where the Pt^{4+} ions was then reduced by the bridging hydrogen atoms in HB, along with the split of hexagonal boron network, due to the breakup of the terminal B-H bond. The following growth of Pt nanoparticles was on the basis of the *in-situ* formed Pt clusters that has been already anchored on the surface of C through B layer to form Pt/B/C.

***Comment 3.** This work seems emphasizing the concept of Pt@B module. Was this type of idea proposed before?*

Response: There is some literature proposing Pt on graphene (*J. Am. Chem. Soc. 2016, 138, 18, 5753–5756; Energy Environ. Sci., 2019,12, 1000-1007*), but no one has

proposed Pt on borophene. That's one of the things we found innovative about this article.

From the reviewer’s comments, the Pt@B module statement in the previous manuscript may cause the readers to be confused about the sample preparation steps. We also noticed that the naming of Pt@B might cause misunderstanding about the structure of the as-prepared samples because A@B usually means a core-shell structure. Therefore, we have replaced Pt@B with Pt/B, and the Pt@B/C was revised to the Pt/B/C module catalysts, because a series of subsequent production of PtM/B/C were based on the Pt/B/C.

Comment 4.*TEM is a local technique and based on the shapes of the particles, I feel it is hard to estimate the particle size. What is the size of Pt based on XAS data?*

Response: Thanks for the reviewer’s suggestions. As we know, TEM is an intuitive approach to estimating particle shape and size, and this technique is widely used in many articles. Here are some examples (*Angew. Chem. Int. Ed. 2023, 62, e202302134; J. Am. Chem. Soc. 2020, 142, 33, 14190–14200; Joule, 2019, 3, 124-135; Energy Environ. Sci. 2022, 15, 278-286; J. Am. Chem. Soc. 2018, 140, 2926−2932; Angew. Chem. Int. Ed. 2020, 59, 7857 – 7863; Adv. Mater. 2023, 35, 2207995)*. In addition, the results are based on a large number of particles and multiple images to ensure accuracy.

The size information obtained from the EXAFS analysis is an empirical method that relies on a large number of databases of particle size and coordination number of the same ligand atomic system, the implementation process is also very complex as well. (J.T. Miller, A.J. Kropf, Y. Zha, J.R. J. Catal.240, 2, 222-234 (2006)) In fact, in our country, machine time for synchrotron radiation is precious, and we don't have enough resources to conduct these experiments.Therefore, we measured the particle size by increasing the TEM field of view and multiple measurements to increase detecting accuracy, as shown in Figure3c-f.

Comment 5. *Lines 156-159, “As shown in Supplementary Figs.4, the TEM images of*

Pt@B/C acquired from 25℃ to 1000 ℃ and corresponding size analysis show the well-maintained dispersion and limited growth of nanoparticles." What are the results from the corresponding size analysis? The size distributions of Pt after different temperatures are not shown in Fig. S4.

Response: We are grateful for the reviewer's comment on the detail about TEM and we have added the corresponding size distribution.

Modification in the Supporting Information:

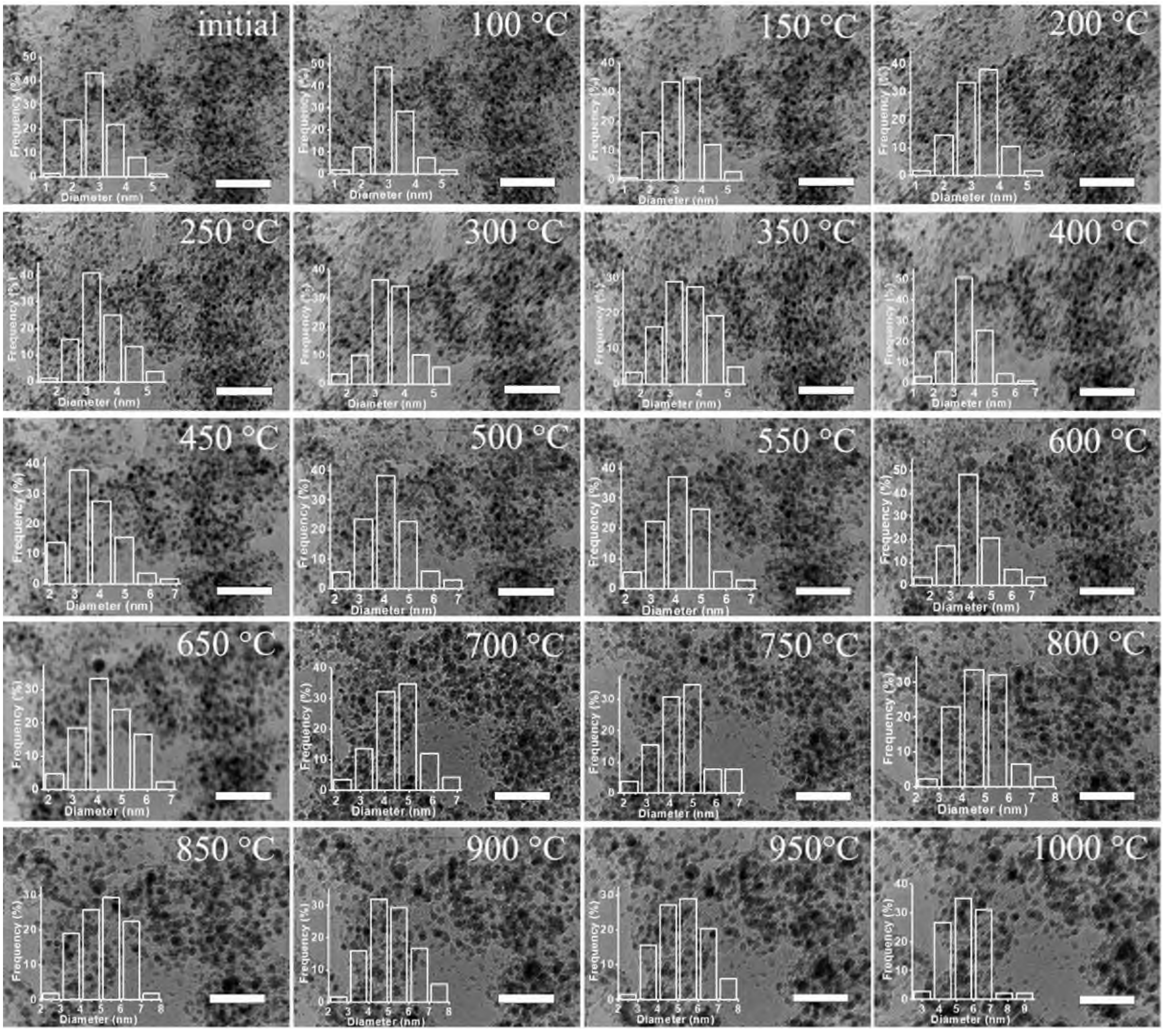

Supplementary Figure 6. In-situ TEM images of Pt/B/KB from room temperature to 1000 ℃. The loading amount of Pt is 60 wt.% and the scale bar is 40 nm.

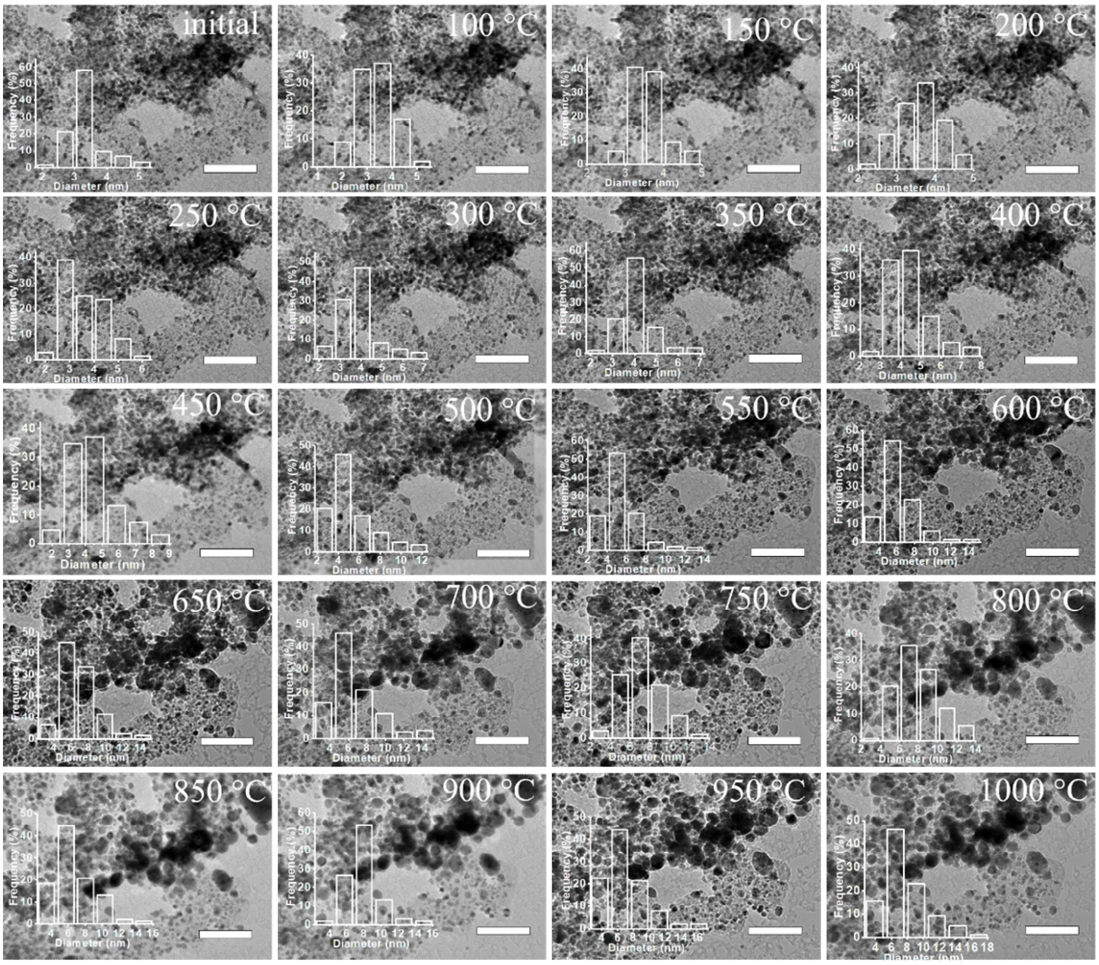

Supplementary Figure 8. In-situ TEM images of commercial Pt/C from room temperature to 1000 ℃. The loading amount of Pt is 60 wt.% and the scale bar is 50 nm.

Comment 6. *I think for catalysts, their stability under reactive conditions is the big challenge. I would suggest the authors testing the stability of Pt@B under temperature dependent reducing conditions.*

Response: Thanks for the comment about catalyst stability in reducing conditions. We have performed an ex-situ sintering experiment at a reducing atmosphere of H_2/Ar mixture gas up to 600 ℃, as shown in Figure 2a (original manuscript), and we continued to raise the annealing temperature to 1000 ℃ (**Figure R3-5**). Generally,

increasing the loading amount will shorten the particle spacing which accelerates the sintering of metal nanoparticles especially at such high temperatures. According to the TEM images, we can see that the 60 wt.% Pt/B/C sample only underwent a slight Oswald ripening at 1000 ℃, while the commercial Pt/C is heavily sintered at 600 ℃. This experiment also demonstrates that the presence of B creates a strong anchoring effect between Pt and C support even in reducing conditions.

Correspondingly, we have also made some modifications in the revised manuscript.

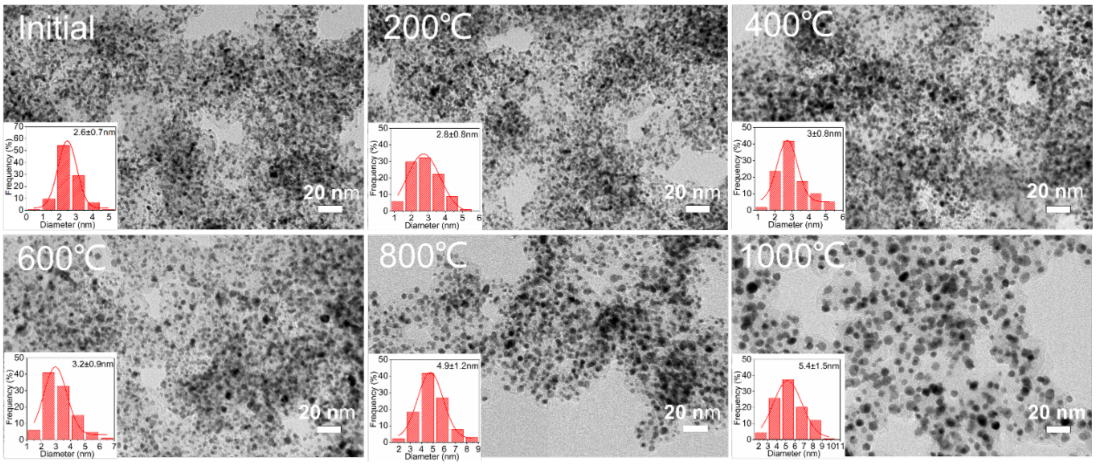

Figure R3-5. Ex-situ TEM images of Pt/B/C from room temperature to 1000℃. The holding time for each temperature stage is 2 hours.

Modification in the revised manuscript:

(1) line 169

To avoid the influence of transmission electron beam on the morphology of the catalyst during the in-situ observation and evaluate the stability of Pt/B/C under reducing conditions, the catalyst was subjected to annealing at different temperatures for 2 hours in a tube furnace under a 5% H_2/Ar atmosphere. The resulting morphology was characterized by TEM, and the corresponding images are presented in Supplementary Fig. 10.

Modification in the Supporting Information:

(1) **Figure R3-5** was listed in revised **Supplementary Information** as **Supplementary Figure 10**.

Comment 7. *According to XPS (fig. 2d), Pt^{2+} and Pt^{0} coexist. What is Pt^{2+} species? Can you see this species from XAS data?*

Response: We truly appreciate the reviewer's comment about XPS. Generally, XPS and XAS data of the same element are consistent and the non-zero valence state in XPS should be represented in XAS (XANES or EXAFS). According to our XPS, Pt^{2+} exists in the Pt/B sample, which may be attributed to Pt-B or Pt-O. We further analyzed the data by using IFEFFIT software (Athena) to correct the background signal, calibrate the energy scale and normalize the intensity of the shock wave. The refitted spectra are shown in **Figure R3-6**. From the spectra, we can see that the peak at 1.68 Å in Pt/B is attributed to Pt-O compared to PtO_2. In addition, the data were also determined by multiple-shell fitting in R space with the application of k^3 and k^1 weightings in the Fourier transformations by software (Artemis) (**Figure R3-7**). The structural parameters extracted from EXAFS fitting at Pt L_3-edge in R space show that Pt/B consists of two scattering paths (Pt-Pt and Pt-O) (**Table 3-1**). So, we attribute the Pt^{2+} in Pt/B to Pt-O. While in the sample of Pt/B/C, the Pt-B bond can be observed, and the details are discussed in the response to Comments 8 and 12.

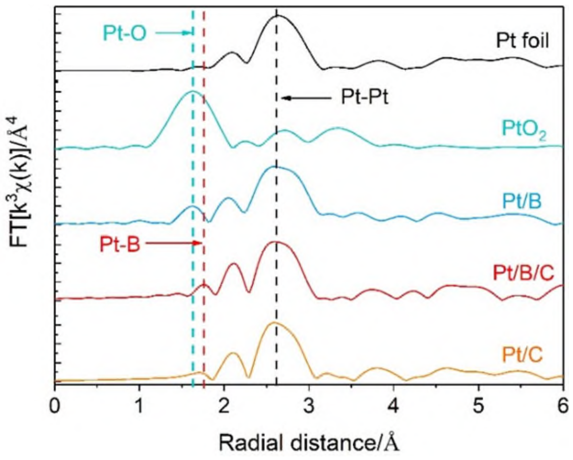

Figure R3-6. EXAFS spectra at Pt L3-edge for Pt foil, PtO_2, Pt/B, Pt/B/C, commercial Pt/C in R space.

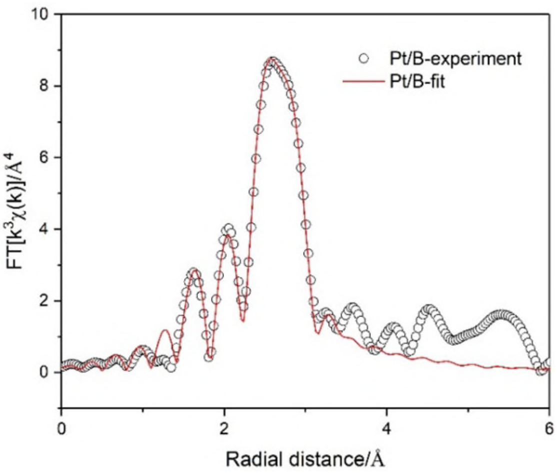

Figure R3-7. EXAFS fitting spectra at Pt L_3-edge of Pt/B.

Table R3-1. Structural parameters extracted from EXAFS fitting at Pt L_3-edge of Pt foil, PtO_2, Pt/B/C, Pt/B in R space.

Sample	Path	CN	R(Å)	σ^2*10^{-3}(Å)	ΔE_0(eV)	R-factor
Pt foil	Pt-Pt	12.00	2.76	-	-	-
PtO_2	Pt-O	6	2.02	-	-	-
Pt/B	Pt-Pt	6.17±0.47	2.76±0.00	1.31±0.32	8.82±0.80	0.003
	Pt-O	0.71±0.24	2.03±0.02	3.23±0.22	13.49±0.92	
Pt/B/C	Pt-Pt	4.71±0.39	2.75±0.02	6.52±0.62	9.05±1.03	0.010
	Pt-B	1.23±0.11	2.22±0.05	5.90±0.75	-11.38±1.75	

*CN, coordination number; R, distance between absorber and backscatter atoms; σ^2, change value of Debye-Waller factor relative to the reference compound; ΔE_0, inner potential correction between the sample and the reference compound. Error bounds (accuracies) of the structural parameters are estimated to be: CN ±20%; R, ±2%; $\Delta\sigma^2$, ±20%; and ΔE_0, ±20%.

Comment 8. *Lines 222-223, "……scattering peak of Pt@B at ~2.45 Å, which is smaller than Pt foil, can be assigned to the contribution of Pt-Pt/Pt-B bonds." I don't think it*

can be assigned to Pt-B bonds. Cannot be that long.

Response: We truly appreciate the reviewer's comment on XAS. In the original manuscript, some peaks in the EXAFS spectra were incorrectly attributed due to the lack of referenceable standard samples. In the revised version, we reanalyzed the original XAS data. Before analysis, by consulting the Crystallography Open Database (COD) developed by the University of Cambridgeit (www.crystallography.net), the slight difference in lengths of Pt-O (2.02 Å), Pt-B (2.19 Å) bond was found. So, we raised the Fourier transform weight in FT-EXAFS spectrum from k^2 in original manuscript to k^3 latest revised manuscript, which can increase the resolution to distinguish Pt-O and Pt-B. From the EXAFS spectrum of Pt/B/C, an apparent peak in 1.78 Å indicating the Pt-B bond and no sign of Pt-O occurs compared to Pt/C and PtO_2 (**Figure R3-6)**. In addition, the structural parameters extracted from EXAFS fitting at Pt L_3-edge in R space further confirms the existence of Pt-B bond with bond length of 2.22 Å (**Figure R3-8, Table 3-1**) in the Pt/B/C sample, different from Pt-O (2.02 Å) bond. As noted by the reviewer, the bond length of Pt-B is indeed not that long.

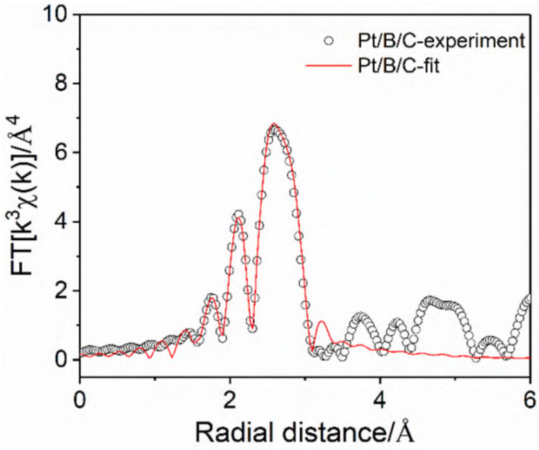

Figure R3-8. EXAFS fitting spectra at Pt L_3-edge of Pt/B/C.

Comment 9. *Lines 223-224, "Due to heteroatomic interactions in Pt@B, a shortened Pt-Pt distance is displayed compared to the standard Pt foil." Do not understand this sentence.*

Response: Thanks for the comment. Our statement may cause reader to be confused. According to reviewer's suggestion, we have revised this part.

Modification in the revised manuscript:

Line 234

Fourier transform of EXAFS data in *R* space is shown in Fig. 2f. Compared to Pt-O bond (located at 1.63 Å) in PtO_2, the apparent peak located at 1.78 Å can be assigned to Pt-B scattering. Subsequent fitting results further confirmed the existence of Pt-B bond in Pt/B/C with a bond length of 2.22 Å (Supplementary Fig. 17 and Table 2), consistent with the computation model after relaxation in DFT calculation (Supplementary Fig. 14). Furthermore, the corresponding wavelet transformed (WT)-EXAFS analysis was performed, which is a precise approach to distinguish the backscattering atoms even though they overlap substantially in R space. As shown in Fig. 2g, the Pt/B/C sample exhibits two maximums on y axis, one is located at *ca.* 2.8 Å which is ascribed to the Pt-Pt coordination, consistent with Pt foil reference, and the other ($R+\alpha$: *ca.* 2.2 Å) which is different from Pt-O ($R+\alpha$: *ca.* 2 Å) in PtO_2 reference can be assigned to the Pt-B bond. The combination of these analyses conclusively demonstrates that the Pt is loaded on borophene, enhancing the adhesive energy between nanoparticles and the B/C support while suppressing aggregation and sintering of Pt nanoparticles at elevated temperatures.

Comment 10. *Please show y scales in Fig 2g, h. Based on Fig. 2g, the intensity of oscillation of Pt@B is weaker than that of Pt foil but the peak intensity of Pt@B in R space appears larger.*

Response: We sincerely appreciate the reviewer's comment about the detail in the k-R space. Because there is no intensity normalization of the data in the previous manuscript, herein according to the reviewer's suggestion, we refitted the data by using IFEFFIT software (Athena) to correct the background signal, calibrate the energy scale and

normalize the intensity of shock wave. For comparison, we adjusted the y scales to keep each graph with a similar size as shown in **Figure R3-6.**

Correspondingly, such a figure was also modified in the revised manuscript.

Comment 11. *Inconsistency of R space spectra and WT-EXAFS maps. In WT-EXAFS, somehow Pt-B/O contribution shifts to higher R. As said,* Pt^{2+} *was observed from XPS. Can the low R contribution due to Pt-O?*

Response: We sincerely thank the reviewer's comment. As mentioned by the reviewer, Pt^{2+} was indeed observed in our XPS which means that Pt has lost some of its electrons to B or O. The R may be different in Pt-O between Pt^{2+} and Pt^{4+} in WT-EXAFS maps. However, we can't distinguish it since PtO_2 is generally used as a reference sample. As mentioned in our response to **Comment 8**, the structural parameters extracted from EXAFS fitting at Pt L_3-edge in R space show that Pt/B consists of two scattering paths (Pt-Pt and Pt-O). So, the intensity maxima in WT-EXAFS of Pt/B is attributed to Pt-O.

Comment 12. *Can the authors distinguish Pt-O and Pt-B bond? What are their bond distances? What is the Pt-Pt coordination number? What is the size of Pt based on EXAFS analysis?*

Response: Here are four questions, which we will answer one by one. If the question overlaps with the previous responses, we will mark the serial number of previous comment.

Distinguish Pt-O and Pt-B bond: **Comment 8 and 11**

Pt-O and Pt-B bond distances: **Comment 8**

Pt-Pt coordination numbe: **Comment 7, Table R3-1**

Size of Pt based on EXAFS analysis: **Comment 4**

Comment 13. *Lines 270-271, "……which can be assigned to the mix of Pt-Co, Pt-B, Pt-O coordination (Fig. 3i), and the…." Where is this O coming from?*

Response: We truly thank the reviewer's comment about the wavelet analysis of PtCo/B/C and we revised this section carefully. This is a clerical error and we have corrected it to Pt-Pt.

Based on comments 7-13, we have made major revision in XAS. Since the high temperature annealing experiments and subsequent alloys are based on Pt/B/C and the appearance of Pt/B is associated with Pt/B/C rather than appearing alone, we used Pt/B/C instead of Pt/B to study the Pt-B bond. In addition, commercial Pt/C was also analyzed for comparison.

Modification in the revised manuscript:

(1) Line 234

Fourier transform of EXAFS data in *R* space is shown in Fig. 2f. Compared to Pt-O bond (located at 1.63 Å) in PtO_2, the apparent peak located at 1.78 Å can be assigned to Pt-B scattering. Subsequent fitting results further confirmed the existence of Pt-B bond in Pt/B/C with a bond length of 2.22 Å (Supplementary Fig. 17 and Table 2), consistent with the computation model after relaxation in DFT calculation (Supplementary Fig. 14). Furthermore, the corresponding wavelet transformed (WT)-EXAFS analysis was performed, which is a precise approach to distinguish the backscattering atoms even though they overlap substantially in R space. As shown in Fig. 2g, the Pt/B/C sample exhibits two maximums on y axis, one is located at *ca.* 2.8 Å which is ascribed to the Pt-Pt coordination, consistent with Pt foil reference, and the other ($R+\alpha$: *ca.* 2.2 Å) which is different from Pt-O ($R+\alpha$: *ca.* 2 Å) in PtO_2 reference can be assigned to the Pt-B bond. The combination of these analyses conclusively demonstrates that the Pt is loaded on borophene, enhancing the adhesive energy between nanoparticles and the B/C support while suppressing aggregation and sintering of Pt nanoparticles at elevated temperatures.

(2) line 281

This was also confirmed by Pt L_3-edge EXAFS results, which indicated the presence of Pt-B bonds in PtCo/B/C samples annealed at 1000℃ in a 5% H_2/Ar atmosphere, demonstrating the robustness of the Pt-B bond (Fig. 3h). Furthermore, the Pt EXAFS analysis confirmed lattice contraction in PtCo/B/C. This compressive strain is known to enhance activity of Pt through tailoring the electronic state of Pt surface through a downshift of the *d*-band center[48]. The fitting results of the EXAFS revealed three scattering paths in PtCo/B/C: Pt-Pt, Pt-Co, and Pt-B (Supplementary Fig. 29 and Table 2). Notably, the Pt-Pt bond length in Pt/B/C (2.70 Å) was found to be shorter than that of Pt foil (2.76 Å), due to the incorporation of smaller Co atoms into the Pt crystal lattice. Moreover, the WT-EXAFS of the Pt L_3-edge signal of PtCo/B/C indicates their intensity maximum consists of three intensity maxima (Fig. 3i) which is consistent with fitting results.

(3) Fig.2 has been updated.

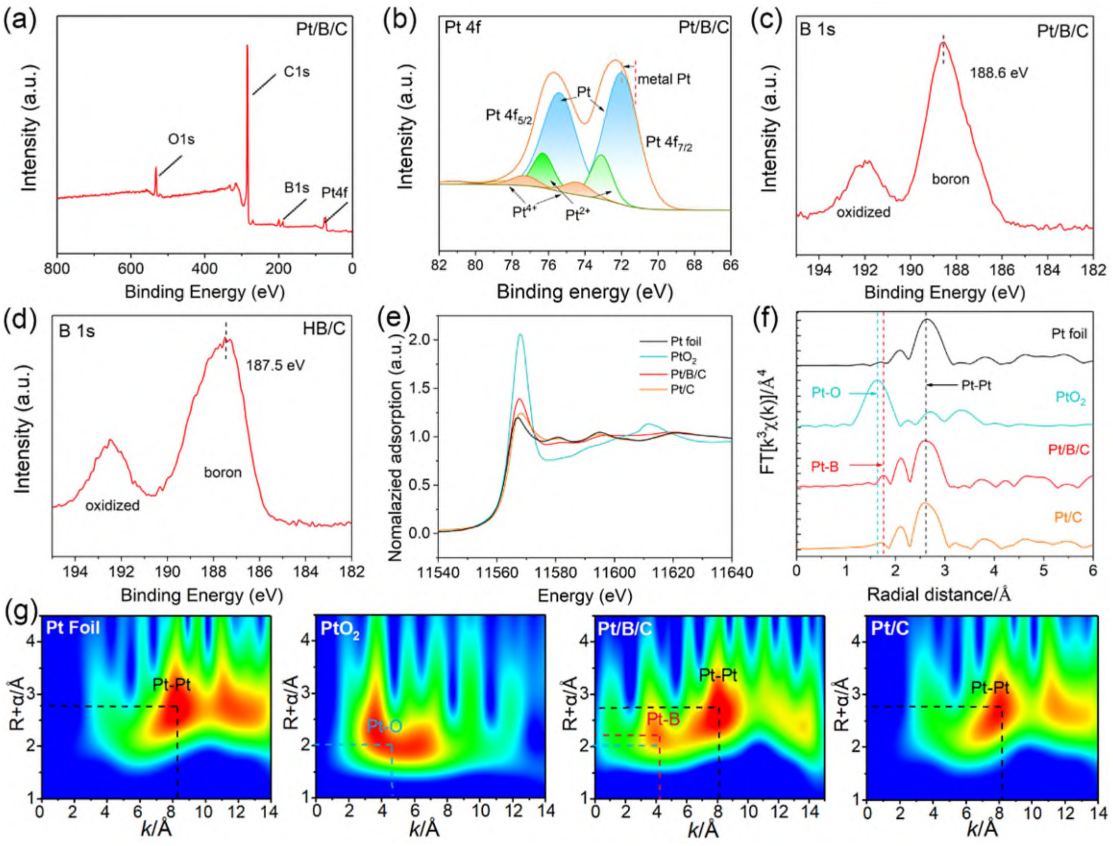

Fig.2 | Interaction of Pt-B analyzed in the experiment. (a) survey spetra XPS of

Pt/B/C. (b) Pt 4f XPS of Pt/B/C. (c) B 1s XPS of Pt/B/C. (d) B 1s XPS of HB/C. (e) Pt L_3-edge XANES spectra of Pt/B/C, along with PtO_2, Pt foil and commercial Pt/C. (f) Pt L_3-edge EXAFS spectra of Pt/B/C, with PtO_2, Pt foil and commercial Pt/C. (g) WT-EXAFS of the Pt L_3-edge signal of the Pt foil, PtO_2, Pt/B/C and commercial Pt/C.

(4) Fig.3 has been updated.

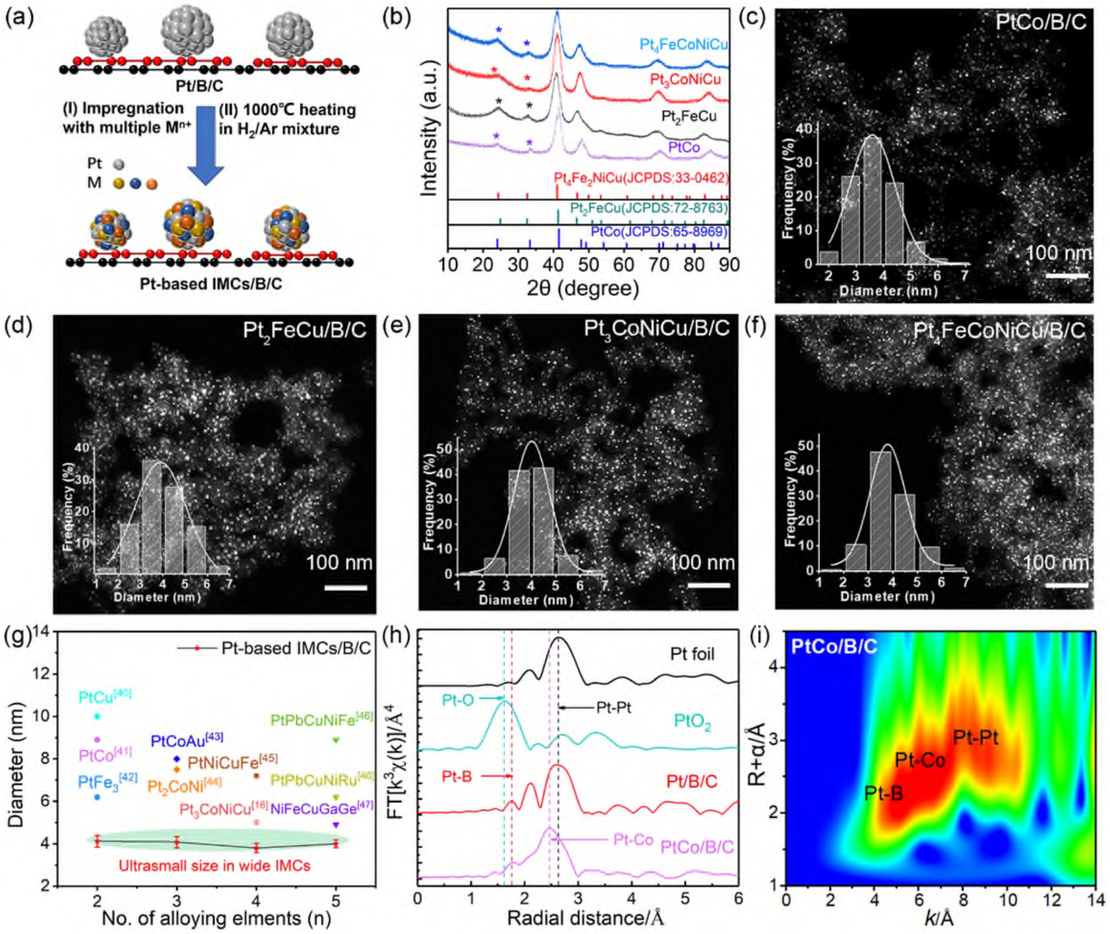

Fig. 3 | Synthesis of ultrafine Pt-based IMCs based on Pt/B/C (a) Schematic illustration of the process of Pt-based multielement intermetallic catalysts. (b) Powder XRD patterns of Pt-based intermetallic alloy. (c-f) HAADF-STEM images and size distribution of intermetallic alloy PtCo/B/C, Pt_2FeCu/B/C, Pt_3CoNiCu/B/C, Pt_4FeCoNiCu/B/C. (g) Comparison of intermetallic alloy size between our work with the state-of-art in the literature. (h) Pt L_3-edge EXAFS spectra of PtCo/B/C, along with Pt/B/C, PtO_2 and Pt foil in *R* space. (i) WT-EXAFS of the Pt L_3-edge signal of PtCo/B/C.

Modification in the Supplementary Information:

(1)

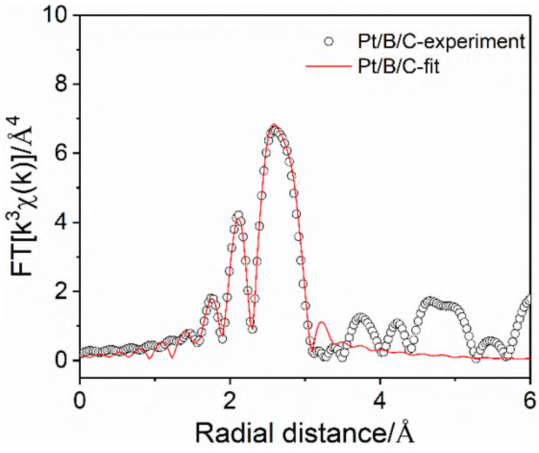

Supplementary Figure 17. EXAFS fitting spectra at Pt L_3-edge of Pt/B/C.

(2)

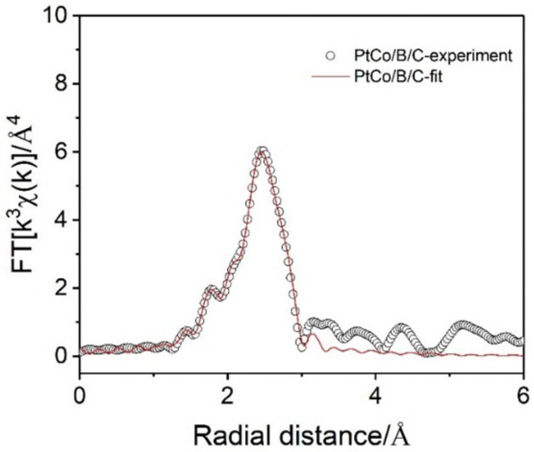

Supplementary Figure 29. EXAFS fitting spectra at Pt L_3-edge of PtCo/B/C.

(3) **Supplementary Table 2.** Structural parameters extracted from EXAFS fitting at Pt L_3-edge of Pt foil, PtO_2, Pt/B/C, PtCo/B/C in R space.

Sample	Path	CN	R(Å)	σ^2*10^{-3}(Å)	ΔE_0(eV)	R-factor
Pt foil	Pt-Pt	12.00	2.76	-	-	-
PtO_2	Pt-O	6	2.02	-	-	-
Pt/B/C	Pt-Pt	4.71±0.39	2.75±0.02	6.52±0.62	9.05±1.03	0.010
	Pt-B	1.23±0.11	2.22±0.05	5.90±0.75	-11.38±1.75	
PtCo/B/C	Pt-Pt	4.82±0.49	2.70±0.00	8.76±0.50	5.95±0.42	0.008
	Pt-Co	2.57±0.57	2.67±0.02	9.63±0.79	9.64±0.77	
	Pt-B	1.12±0.15	2.22±0.03	7.38±0.37	4.42±0.35	

*CN, coordination number; R, distance between absorber and backscatter atoms; σ^2, change value of Debye-Waller factor relative to the reference compound; ΔE_0, inner potential correction between the sample and the reference compound. Error bounds (accuracies) of the structural parameters are estimated to be: CN ±20%; R, ±2%; $\Delta\sigma^2$, ±20%; and ΔE_0, ±20%.

REVIEWER COMMENTS

Reviewer #1 (Remarks to the Author):

The authors have substantially addressed all my comments and I support publication of the manuscript in its current form.

Reviewer #2 (Remarks to the Author):

Manuscript ID: NCOMMS-23-12450-A
Title: Hydrogenated borophene enabled synthesis of ultrafine multielement catalysts

Comments)
The article by Zeng et al. has improved in quality and depth following the initial revision, the reviewer is no doubt its contribution. Given this, I believe the piece is fitting for publication in Nature Communication. However, based on your latest response, several new concerns have come up. Before final approval, the authors must address the critical issues raised below in the subsequent, finalized version of the article. Please see the details below.

1. The authors removed the TEM image of Pt/B from the original draft without explanation. This image can provide evidence that there is no 2D borophene shape present after the synthesis of Pt@B module. I could not find even in the supporting information.
2. In the rebuttal letter, the authors explained the existence of Pt/(BC) bonding by controlling the order in which the Pt precursors were added, supporting this with EAS analysis. However, in a prior work by the same group [Appl. Surf. Sci. 604 (2022) 154466], boron was doped with carbon using H3BO3 as a boron source and reductant. The paper outlines a procedure where H3BO3 and metal precursors were initially mixed, followed by the addition of carbon. Nevertheless, the authors claimed this resulted in the synthesis of Pt/(B-doped-C). Like in this case, can it be inferred from the experimental sequence that boron is positioned between Pt/C? There are existing reports on boron being doped or intercalated into metal lattices, and due to the lack of direct evidence of a 2D structure in this case, it is considered that the possibility of boron being located in the Pt lattice should not be ruled out. You can refer that literatures highlight various instances of boron doping with platinum group metals or in an interstitial configuration [ACS Catal. 2022, 12, 15, 8848-8856] [Nat. Comm. (2014) 5:5787].
3. In the modified Figure 2-(d), an oxidized peak is observed around 192~193 eV. This contrasts with the previous research [J. Am. Chem. Soc. 2017, 139, 13761−13769] [Nat. Comm. (2019) 10:4880], which depicted one predominant B 1s peak at around 187 eV. Does this additional peak suggest potential carbon-induced oxidation of HB? Or could the substance really be HB? Unlike many studies that synthesized HB carried out various analysis (such as FT-IR, XPS, UV-vis, etc.), the analysis applied to the synthesized HB in this manuscript might have been insufficient.
4. B/Pt/C was synthesized and compared with Pt/B/C. Could you verify the proper incorporation of boron, at least through ICP analysis?

Reviewer #3 (Remarks to the Author):

The questions have been addressed.

Manuscript ID: NCOMMS-23-12450-A

Title: Response to Reviewer Comments on the Paper " Hydrogenated borophene enabled synthesis of ultrafine multielement catalysts "

Response Letter to Reviewers

(*Black italic: Editor's remarks*; Blue type: Our response; Red type: Our revision)

Point-by-Point Response to Reviewers' Comments

Reviewer #1:

The authors have substantially addressed all my comments and I support publication of the manuscript in its current form.

Response: We sincerely appreciate the reviewer's recognition of the revised manuscript and the article has been improved because of your valuable comments.

Reviewer #2:

The article by Zeng et al. has improved in quality and depth following the initial revision, the reviewer is no doubt its contribution. Given this, I believe the piece is fitting for publication in Nature Communication. However, based on your latest response, several new concerns have come up. Before final approval, the authors must address the critical issues raised below in the subsequent, finalized version of the article. Please see the details below.

Response: We are very grateful to the reviewer for the recognition and support for the publication of our paper. Below we addressed your additional suggestions point-by-point.

Comment 1. *The authors removed the TEM image of Pt/B from the original draft without explanation. This image can provide evidence that there is no 2D borophene shape present after the synthesis of Pt@B module. I could not find even in the supporting information.*

Response:

Thank you for the suggestion. We would like to acknowledge that Pt/B (Pt@B in the initial manuscript) is indeed an intermediate product formed during the synthesis of PtM/B/C. Unfortunately, the inclusion of certain characterizations of Pt/B led to some misunderstandings among the reviewers. To address this issue, we have decided to remove all characterizations related to Pt/B from the manuscript. However, based on the reviewer's suggestion, we recognize the importance of preserving the TEM image

of Pt/B. Therefore, we have included the image of Pt/B as supplementary Fig. 3 in the supplementary information section.

Modification in the revised manuscript:

(1) line 118

In the experiment, we conducted a two-step synthesis procedure to fabricate the catalyst, as illustrated in Figure 1a. Firstly, the as-prepared HB was mixed with activated carbon to form HB/C support. Subsequently, a solution containing H_2PtCl_6 was introduced, where the Pt^{4+} ions was then reduced by the bridging hydrogen atoms in HB, along with the split of hexagonal boron network, due to the breakup of the terminal B-H bond (supplementary Fig. 3).

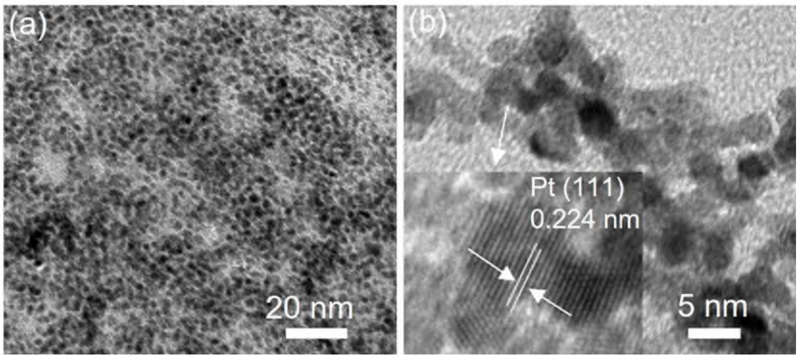

Supplementary Figure 3. (a-b) TEM image and high-resolution TEM of Pt/B. Synthesis method: 10 mL HB solution and 266 mg $H_2PtCl_6\cdot 6H_2O$ dissolved in 200 ml methanol were mixed. After being stirred for three days, the Pt/B was obtained.

Comment 2. *In the rebuttal letter, the authors explained the existence of Pt/(BC) bonding by controlling the order in which the Pt precursors were added, supporting this with EAS analysis. However, in a prior work by the same group [Appl. Surf. Sci. 604 (2022) 154466], boron was doped with carbon using H3BO3 as a boron source and reductant. The paper outlines a procedure where H3BO3 and metal precursors were initially mixed, followed by the addition of carbon. Nevertheless, the authors claimed this resulted in the synthesis of Pt/(B-doped-C). Like in this case, can it be inferred from the experimental sequence that boron is positioned between Pt/C? There are existing reports on boron being doped or intercalated into metal lattices, and due to the lack of direct evidence of a 2D structure in this case, it is considered that the possibility of boron being located in the Pt lattice should not be ruled out. You can refer that literatures highlight various instances of boron doping with platinum group metals or in an interstitial configuration [ACS Catal. 2022, 12, 15, 8848-8856] [Nat. Comm. (2014) 5:5787].*

Response:

Thank you for your suggestion. (1) In our previous study (Appl. Surf. Sci. 604 (2022) 154466), we synthesized Pt NPs dispersed on boron-doped carbon, denoted as Pt/(B-

C), by pyrolyzing a mixture of $H_2PtCl_6{\cdot}6H_2O$, H_3BO_3, and Vulcan XC-72 carbon. In that case, the platinum precursor was reduced using H_2/Ar instead of H_3BO_3. H_3BO_3 was used as a dopant at 600 °C to incorporate boron into the carbon, forming a Pt/(B-doped-C) catalyst. The initial mixing of H_3BO_3 and metal precursors, followed by the addition of carbon, was done to ensure an evenly mixed precursor before annealing. However, in our current work, we employed HB as a reductant that was first mixed with the support material, such as carbon, to form HB/C. Subsequently, the Pt^{4+} ions were reduced by the bridging hydrogen atoms in HB. The subsequent growth of Pt nanoparticles occurred based on the in-situ formed Pt clusters that had already been anchored on the surface of carbon through a boron layer, resulting in the formation of Pt/B/C at room temperature. Therefore, we infer that boron is positioned between Pt and the carbon support. In response to the reviewer's suggestion, we thoroughly examined two relevant literature sources on boron doping. These studies provided evidence of B-doping after high temperature treatment through TEM and XRD analyses. The comparative high-resolution STEM images of typical metal nanoparticles revealed Pt (111) lattice spacings of 0.227 nm for Pt/C (commercial) and 0.231 nm for Pt-B/C, indicating a lattice expansion in Pt-B/C. The corresponding XRD peaks for Pt-B/C shifted to lower angles, suggesting a slight lattice expansion due to interstitial B-doping in the (ACS Catal. 2022, 12, 15, 8848-8856). Motivated by these findings, we conducted XRD and TEM analyses of Pt/B/C with or without high temperature treatment in our study. As shown in Figure R1, both the TEM results (the Pt (111) lattice spacing for Pt/B/C is measured to be 0.224 nm, smaller than that of Pt/C (commercial)) and XRD data (the corresponding peaks for Pt/B/C did not shift to lower angles even after annealing Pt/B/C at 1000 °C for an hour in an H_2/Ar atmosphere) demonstrate that there is no lattice expansion in Pt. This indicates that boron is not located within the Pt lattice.

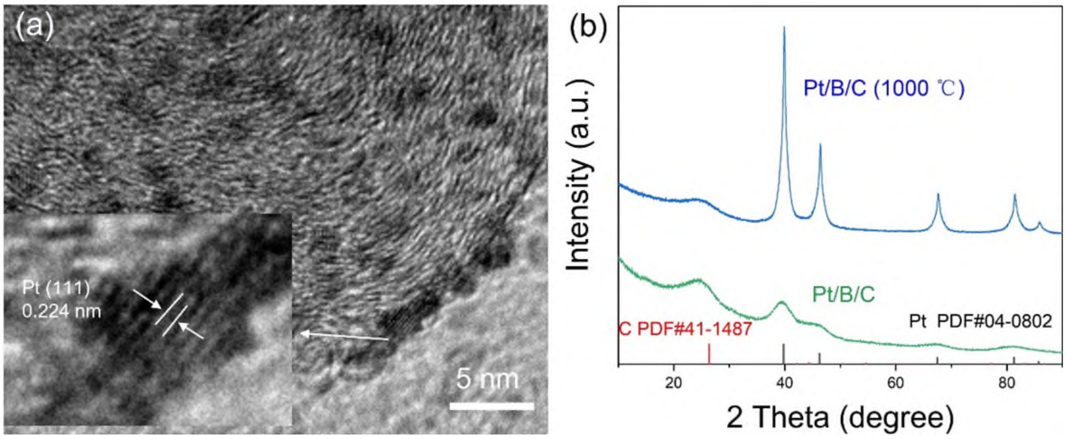

Figure R1. (a) TEM and (b) XRD of Pt/B/C.

Comment 3. *In the modified Figure 2-(d), an oxidized peak is observed around 192~193 eV. This contrasts with the previous research [J. Am. Chem. Soc. 2017, 139, 13761−13769] [Nat. Comm. (2019) 10:4880], which depicted one predominant B 1s peak at around 187 eV. Does this additional peak suggest potential carbon-induced*

oxidation of HB? Or could the substance really be HB? Unlike many studies that synthesized HB carried out various analysis (such as FT-IR, XPS, UV-vis, etc.), the analysis applied to the synthesized HB in this manuscript might have been insufficient.

Response:

Firstly, the preparation of HB follows the methods described in a previous literature source [*J. Am. Chem. Soc. 2017, 139, 13761−13769*]. The literature explains that the byproduct $B(OH)_3$ is formed through the hydrolysis reaction of HB sheets with water absorbed in the ion-exchange resin. Regarding the higher oxidation peak observed in our experiment compared to the literature, it may be attributed to the water content present in the materials (MgB_2, ion exchange resin, of course, this is also closely related to the purity of MgB_2 that we used), as well as the fact that the subsequent experimental processes were conducted under ambient conditions, leading to surface oxidation. Consequently, the peak at ~193 eV appears higher. However, it is important to note that the dominant peak in the HB/C sample still corresponds to the negatively charged boron species in HB. Furthermore, in our previous work [*ref. 21, J. Mater. Chem. A, 2020, 8, 18856*], we utilized FT-IR and EELS techniques to characterize HB, and these characterization results are well consistent with those mentioned in the aforementioned JACS study, as shown in Fig.R2. Therefore, considering the high consistency of the preparation method, we did not conduct an in-depth characterization of the structure of HB in this study.

To enhance reader comprehension, we have also included the *ref. 21* in the experimental section.

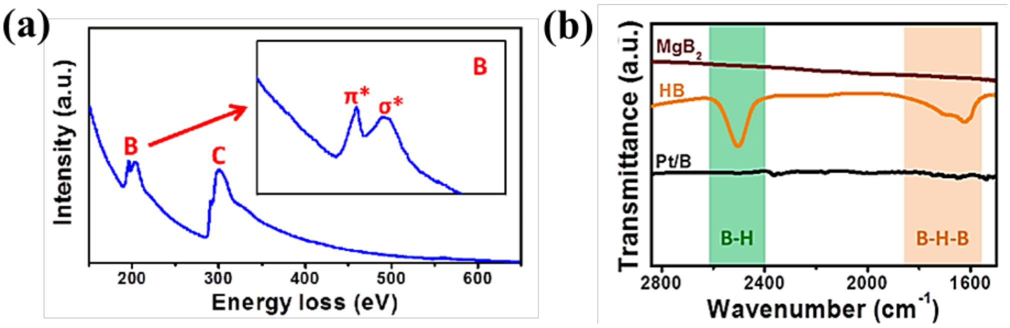

Figure R2. The characterizations of HB in our provious work (*ref. 21),* (a) EELS spectrum of the HB sheets and (b) FT-IR of Pt/B, MgB_2 and HB.

Comment 4. *B/Pt/C was synthesized and compared with Pt/B/C. Could you verify the proper incorporation of boron, at least through ICP analysis?*

Response:

Firstly, in the synthesis of B/Pt/C, HB was added to Pt/C (commercial) and allowed to dry, ensuring the incorporation of the B source in the sample. Due to the limitations of ICP in accurately measuring non-metallic light elements, we employed XPS for the characterization of boron. The XPS analysis revealed a relatively low concentration of

B on the Pt surface. However, despite its low abundance, it still remarkably suppressed the catalytic activity of the commercial Pt/C catalyst. Moreover, it should be noted that the boron species on the Pt surface are susceptible to oxidation.

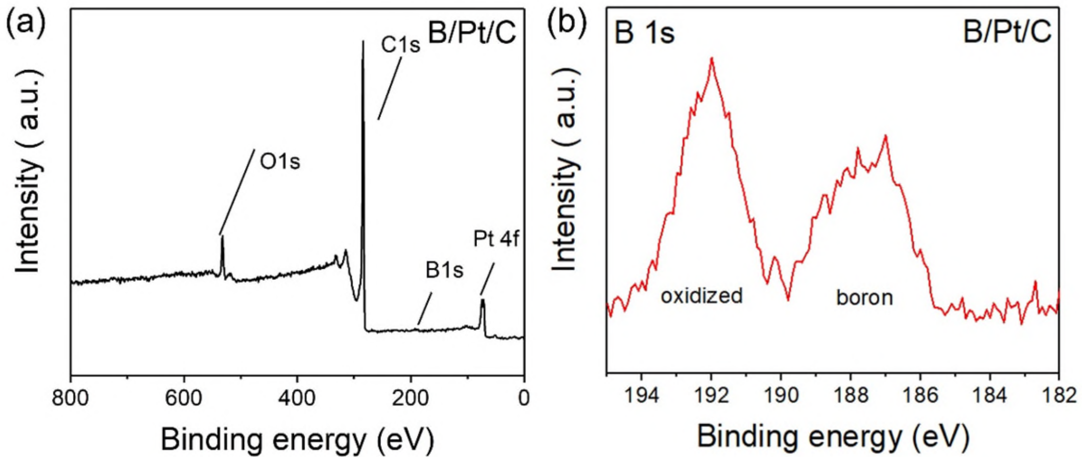

Figure R3. (a) survey spetra XPS of B/Pt/C, (b) B 1s XPS of B/Pt/C.

Modification in the revised manuscript:

(1) Figure R3 was listed in **Supplementary Figure 5** (c) and (d). The caption of such figures were revised correspondingly.

Reviewer #3:

The questions have been addressed.

Response: We sincerely appreciate the reviewer's recognition of the revised manuscript and the article has been improved because of your valuable comments.

REVIEWERS' COMMENTS

Reviewer #2 (Remarks to the Author):

The authors have substantially addressed all my comments and I support publication of the manuscript in its current form.
